# Tumor-Educated Platelet Extracellular Vesicles: Proteomic Profiling and Crosstalk with Colorectal Cancer Cells

**DOI:** 10.3390/cancers15020350

**Published:** 2023-01-05

**Authors:** Annalisa Contursi, Rosa Fullone, Paulina Szklanna-Koszalinska, Simone Marcone, Paola Lanuti, Francesco Taus, Alessandra Meneguzzi, Giulia Turri, Melania Dovizio, Annalisa Bruno, Corrado Pedrazzani, Stefania Tacconelli, Marco Marchisio, Patrizia Ballerini, Pietro Minuz, Patricia Maguire, Paola Patrignani

**Affiliations:** 1 Center for Advanced Studies and Technology (CAST), 66100 Chieti, Italy; 2 Department of Neuroscience, Imaging and Clinical Sciences, “G. d’Annunzio” University, 66100 Chieti, Italy; 3 Conway SPHERE Research Group Ireland, UCD Conway Institute, University College Dublin, D04 C1P1 Dublin, Ireland; 4 Trinity Translational Medicine Institute, Trinity College Dublin, D02 PN40 Dublin, Ireland; 5 Department of Medicine and Aging Sciences, “G. d’Annunzio” University, 66100 Chieti, Italy; 6 Department of Medicine, University of Verona, 37129 Verona, Italy; 7 Department of Surgical Sciences, Dentistry, Gynecology and Pediatrics, Division of General and Hepatobiliary Surgery, University of Verona, 37129 Verona, Italy; 8 Department of Innovative Technologies in Medicine and Dentistry, “G. d’Annunzio” University, 66100 Chieti, Italy

**Keywords:** epithelial-mesenchymal transition, cyclooxygenase-2, thromboxane A_2_, platelet-derived extracellular vesicles, colorectal cancer

## Abstract

**Simple Summary:**

The most life-threatening events in colorectal cancer (CRC) are metastasis and thrombosis. Platelets can play a role in these outcomes via the release of medium-sized extracellular vesicles (mEVs). Thus, we aimed to study the EVs released from activated platelets of CRC patients and healthy controls (HS) for their size composition, protein content, and the capacity to influence the expression of genes involved in malignancy and the synthesis of a prothrombotic lipid mediator such as thromboxane (TX)A_2_. Our findings show that the protein content of thrombin-stimulated mEVs is modulated in CRC. Its evaluation may represent a noninvasive tool to discriminate patients from healthy subjects. Moreover, our findings show that characterizing the regulation of the expression of promalignant genes and prothrombotic phenotypes in cancer cells by the crosstalk with platelet mEVs could provide prognostic information on cancer patients, which could help in developing an appropriate anticancer strategy.

**Abstract:**

Background: Platelet–cancer cell interactions modulate tumor metastasis and thrombosis in cancer. Platelet-derived extracellular vesicles (EVs) can contribute to these outcomes. Methods: We characterized the medium-sized EVs (mEVs) released by thrombin-stimulated platelets of colorectal cancer (CRC) patients and healthy subjects (HS) on the capacity to induce epithelial-mesenchymal transition (EMT)-related genes and cyclooxygenase (COX)-2(*PTGS2*), and thromboxane (TX)B_2_ production in cocultures with four colorectal cancer cell lines. Platelet-derived mEVs were assessed for their size distribution and proteomics signature. Results: The mEV population released from thrombin-activated platelets of CRC patients had a different size distribution vs. HS. Platelet-derived mEVs from CRC patients, but not from HS, upregulated EMT marker genes, such as *TWIST1* and *VIM,* and downregulated *CDH1. PTGS2* was also upregulated. In cocultures of platelet-derived mEVs with cancer cells, TXB_2_ generation was enhanced. The proteomics profile of mEVs released from activated platelets of CRC patients revealed that 119 proteins were downregulated and 89 upregulated vs. HS. Conclusions: We show that mEVs released from thrombin-activated platelets of CRC patients have distinct features (size distribution and proteomics cargo) vs. HS and promote prometastatic and prothrombotic phenotypes in cancer cells. The analysis of platelet-derived mEVs from CRC patients could provide valuable information for developing an appropriate treatment plan.

## 1. Introduction

Cardiovascular disease (CVD) and colorectal cancer (CRC) are among the leading causes of death in Europe [1]. Indirect evidence suggests that mechanisms involved in developing atherothrombosis are shared in CRC [2,3]. The finding of an anticancer effect (mainly against CRC) of the antiplatelet drug low-dose aspirin, recommended for the secondary prevention of atherothrombosis, sustains the contribution of platelet activation to CVD and CRC [2,3].

Platelets can participate in the early phase of tumorigenesis by inducing an inflammatory tumor microenvironment [4]. Platelets can interact with cancer cells causing phenotypic changes in cancer cells, including the induction of epithelial-mesenchymal transition (EMT)-related gene expression and the cyclooxygenase (COX)-2-signaling pathway [5,6]. COX-2-dependent prostanoids play numerous roles in tumor development and metastasis [7,8]. On the other hand, cancer cells can amplify platelet activation, in part by enhancing the synthesis of thromboxane (TX)A_2_ [9], which plays an important role in cancer development [10,11]. These numerous events triggered by the crosstalk between platelets and cancer cells contribute to tumor metastasis [5,7,9].

Another mechanism used by platelets in cancer development, including CRC, can involve the release of extracellular vesicles (EVs), which circulate in plasma. EVs are particles naturally released from the cells; they are delimited by a lipid bilayer and cannot replicate, i.e., they do not contain a functional nucleus [12]. EVs vary in size and biogenesis. Because of the difficulty in defining the sub-cellular origin of an EV, a size-based EV nomenclature has been recommended. Small EVs (sEVs) and medium EVs (mEVs, also known as microparticles, MP), with ranges defined, for instance, respectively, <100 nm and ≥100–1000 nm. EVs include apoptotic bodies typically 1–5 μm in diameter [13].

An increased level of total plasma platelet-derived mEVs has been observed in various types of malignancies, such as gastric, ovarian, breast, and lung cancer [14,15,16,17]. However, the association of platelet-derived mEV count with CRC is controversial [18,19]. High platelet-derived EV counts correlate with tumor aggressiveness and poor clinical outcome [20]. 

Since platelets actively endocytose plasma constituents, the characterization of EVs (size, count, and molecular phenotype) in plasma and those released by activated platelet (named platelet releasate) can be of diagnostic relevance to disease conditions, including CRC [21,22]. 

EVs are recognized as conveyors of intercellular communication through the transmission of biological signals [21]. Platelet-derived mEVs increase the adhesion of tumor cells to endothelial cells and fibrinogen by transferring to the tumor cell surface of various platelet-derived integrins, such as CD41 [23]. They play a role in angiogenesis and the metastatic spread of cancers such as lung cancer [23]. Platelet EVs regulate the proliferation, survival, and adhesion of human normal and malignant hematopoietic cells [23].

We have reported that platelet-derived mEVs of obese women show higher heterogeneity in size and contain different levels of proteins relevant to thrombosis and tumorigenesis [24]. EMT and endothelial-mesenchymal transition (EndMT) marker genes were induced in human colorectal cancer cells and cardiac microvascular endothelial cells (HCMEC), respectively, by the coculture with mEVs from obese individuals [24]. COX-2 was also upregulated in HCMEC by mEVs released from the platelets of obese individuals. Therefore, platelet-derived mEVs can play a role in the enhanced risk of thrombosis and cancer in obesity.

Here, we aimed to characterize platelet-derived mEVs of CRC patients vs. healthy subjects (HS) matched for sex and age on the capacity to induce EMT-related gene expression and COX-2 and TXA_2_ generation in the coculture with four human colon cancer cell lines (with different Duke stages). The platelet-derived EVs from CRC and HS were also characterized for their size distribution and proteomic signatures. 

## 2. Material and Methods

### 2.1. Study Design and Subjects

Sixteen patients with CRC and 16 HS matched for sex and age were enrolled at the University of Verona, Department of Surgical Sciences, Division of General and Hepatobiliary Surgery, Policlinico “G.B. Rossi”, Verona (Italy), and signed informed consent for the study was obtained. The study was conducted following the Declaration of Helsinki, and the Ethics Committee of Policlinico “G.B. Rossi”, Verona (Italy) approved it (clinicaltrials.gov, registration number 73033 on 13 November 2018). The individuals were included in the study if the following criteria were satisfied: (i) age > 18 years; (ii) established diagnosis of CRC according to current diagnostic criteria. The exclusion criteria were the following: (i) individual with a family history of CVD or a personal history of thyroid or pituitary disease, (ii) anorexia or bulimia, (iii) smoking; (iv) pregnant women or who have given birth in the previous 6 months; (v) individuals being treated with aspirin and/or other nonsteroidal anti-inflammatory drugs (NSAIDs) during the month before enrollment. After signing the informed consent, all individuals underwent blood collection to prepare plasma and washed platelets. The clinical features of CRC patients and healthy subjects are shown in Table 1. The age and body mass index (BMI) were not significantly different in the two groups. Nine out of sixteen were females, and only one in both groups had hypertension. 

### 2.2. Preparation of Plasma and Platelet-Derived mEVs

Whole blood samples were collected in citrated plastic tubes (3.8%), and apyrase (Sigma Aldrich, Milan, Italy) (0.4 U/mL) was added; then, it was centrifuged for 20 min at 180× *g* at room temperature (RT; 20–24 °C) without brake to obtain platelet-rich plasma (PRP). The PRP was collected and centrifuged at 700× *g* at RT without brake. The supernatant was centrifuged twice at 2500× *g* for 15 min at RT, avoiding the application of the centrifuge brake, and plasma was snap-frozen in liquid nitrogen and stored at −80 °C. It was then analyzed using flow cytometry to enumerate mEVs. The platelet pellet was gently resuspended in the wash buffer pH 6.5 (103 mmol/L NaCl, 5 mmol/L KCl, 1 mmol/L MgCl_2_, 5 mmol/L glucose, 36 mmol/L citric acid [25]) and centrifuged for 10 min at 700× *g* at RT without brake; platelet pellet was resuspended and washed again as reported above. Then, the platelet pellet was resuspended in HEPES 1 buffer, pH 7.4 (5 mmol/L HEPES, 137 mmol/L NaCl, 2 mmol/L KCl, 1 mmol/L MgCl_2_, 12 mmol/L NaHCO_3_, 0.3 mmol/L NaH_2_PO_4_, and 5.5 mmol/L glucose) [25] and washed platelets were counted and resuspend to obtain 3 × 10^8^ cells/mL. The platelet suspension was incubated with CaCl_2_ (2 mM) and thrombin (1U/mL) for 30 min at 37 °C in the water bath. The tubes were put on ice; protease inhibitors (#A32953, Thermo Fisher Scientific, Milan, Italy), phosphatase inhibitors (#49906845001, Sigma Aldrich, Milan, Italy), and PMSF (phenylmethylsulfonyl fluoride; a serine protease) were added. Then, it was centrifuged for 10 min at 4 °C at 300× *g* without brake, and the supernatant was centrifuged for 15 min at 2000× *g* at 4 °C to eliminate the platelets. Finally, the supernatant was centrifuged at 20,000× *g* for 40 min at 4 °C to pellet mEVs that were snap-frozen in liquid nitrogen and stored at −80 °C. 

### 2.3. Analysis of Plasma, Washed Platelets, and Platelet-Derived mEVs by Flow Cytometry

Plasma aliquots (100 μL), washed platelets (100 μL), and EV pellets (100 μL) were resuspended in Annexin buffer (BD Biosciences, Milan, Italy) with a mix of reagents containing lipophilic cationic dye (LCD, Thermo-Fisher), Phalloidin (Sigma-Aldrich), CD41-PerCP-Cy5.5 (BD Biosciences), AnnexV-V500 (BD Biosciences), and identified and counted by flow cytometry (FACSVerse, BD Biosciences). Phalloidin negative events (of total EVs or LCD positive EVs) were analyzed for their CD41 expression, which recognizes the platelet glycoprotein IIb (ITGA2B); CD41^+^ events were then assessed for their AnnexinV positivity [26]. Plasma was also evaluated with PerCP/Cyanine5.5 anti-human CD31 antibody [CD31 is a 130–140 kD type I transmembrane glycoprotein known as platelet endothelial cell adhesion molecule-1 (PECAM-1) mainly from endothelial cells]. Washed platelet suspension was also characterized for the contamination of leukocytes [positivity to cell-permeant SYTO 16 green-fluorescent nucleic acid stain (Thermo Fisher Scientific) and CD45 (detected by monoclonal antibodies from BD Biosciences)] and erythrocytes (positivity to CD235a detected by monoclonal antibodies from BD Biosciences). Flow cytometry assessments were performed as previously reported [24,26,27]. EV pellets were characterized for forward and side scatter (FSC and SSC, respectively) using Megamix-Plus beads (Biocytex, Marseille, France) following the manufacturer’s instructions. The rosetta calibration system (Exometry, Amsterdam, The Netherlands) measured EV diameters. It establishes the scatter-to-diameter relationship for the side scatter detector of the FACSVerse flow cytometer. In detail, the rosetta calibration beads provided by the kit (Exometry) were run according to the manufacturer’s recommendations, using the same setting applied for EV detection. The relationship between EVs and the diameter was automatically obtained by an algorithm based on Mie theory and assuming a particle refractive index core of 1.40 for EVs, as already reported [26,27,28,29]. Height (H) signals and logarithmic or bi-exponential modes were selected for all parameters. Instrument performances and data reproducibility were monitored by the Cytometer Setup and Tracking Module (BD Biosciences, San Jose, CA, USA). Data were analyzed using FACSuite v 1.0.6.5230 (BD Biosciences) and FlowJo v 10.0.7 (TreeStar, now Becton, Dickinson, and Company, Ashland, OR, USA) software.

### 2.4. Cocultures of Cancer Cells with Platelet-Derived mEVs

Protein concentration of platelet-derived EV preparations was assessed using the Bradford assay (Bio-Rad, Milan, Italy), and 100 μg were used for the coculture experiments with colon cancer cells. The EV concentration was selected based on previous studies [30]. The human colon carcinoma cell lines HT29, HCA-7 Colony 29, HTC116, and Caco2, were purchased from ATCC (Manassas, VA, USA) and cultured following the manufacturer’s instructions. Cells (0.5 × 10^6^) were incubated for 4 or 24 h with mEVs (100 μg) generated from thrombin-activated platelets of CRC and HS. mEVs were assessed for the capacity to cause changes in the expression of marker genes of EMT [*CDH1* (protein name, E-cadherin), *VIM* (protein name, vimentin), and *TWIST1* (protein name, Twist-1)] and *PTGS2* (protein name, COX-2) in colon cancer cells using qPCR [6,9]. In addition, conditioned media were collected, centrifuged to discard cell debris, and the supernatant was stored at −80 °C until assayed for TXB_2_ (the stable, nonenzymatic, hydrolysis product of TXA_2_) by a specific immunoassay [6,31].

### 2.5. mRNA Analysis

Total RNA was extracted from cancer cells treated or not with mEV using Pure link RNA Mini kit (Life Technologies, Milan, Italy) to assess mRNA levels of E-cadherin, Vimentin, Twist, and COX-2 and normalized with GAPDH levels using a 7900HT Real-Time PCR system (Applied Biosystems, Foster City, CA, USA). After removing genomic DNA through a DNAse kit (Life Technology), 1 μg of total RNA was transcribed using iScript cDNA synthesis kit (BioRad, Hercules, CA, USA). One hundred ng of cDNA were used for the reaction mixture. The amplification of *CDH1*, VIM, *TWIST1*, *PTGS2*, and *GAPDH* was performed using TaqMan gene expression assays (Hs01023894, Hs00185584, Hs01675818, Hs00153133, and Hs99999905, respectively) (Applied Biosystems) according to the manufacturer’s instructions using a 7900HT Real-Time PCR system (Applied Biosystems). Gene expression assays were performed by relative quantification with comparative cycle threshold (Ct) using ABI Prism, SDS 2.4 software (Applied Biosystems).

### 2.6. Assessment of Platelet-Derived mEV Proteins by Liquid Chromatography-Mass Spectrometry (LC-MS/MS)

The preparation of samples was previously reported [32]. Bradford assay was used to evaluate the concentration of proteins (Bio-Rad). Acetone (95%; 4:1 acetone: sample volume) was used to precipitate 30 μg of proteins during overnight incubation. As previously described, the proteins were analyzed using LC-MS/MS [32]. We resuspended dried protein pellets using urea (8 M), Tris–HCl (25 mM) (pH 8.2, at 37 °C) in agitation. Dithiothreitol (DTT, 5 mM) was used to reduce disulfide bonds and protect with iodoacetamide (15 mM). Proteins were digested with Lys-C (1:100; Promega, Madison, WI, USA) and trypsin (1:100; Promega). ZipTipC18 pipette tips (Millipore, Billerica, MA, USA) were used for purifying the peptides that, once resuspended (1% formic acid), were analyzed (approximately 5 μg) by LC-MS/MS [ultiMate 3000 HPLC-hybrid quadrupole-orbitrap mass spectrometer (Q Exactive, Thermo Fisher Scientific, Waltham, MA USA)]. A 40 min linear LC gradient (acetonitrile from 2 to 33%), a C18 reverse phase chromatography column (particle size, 2.4 μm, pore size, 300 Å, C18 material, length, 120 mm, ID: 75 μm; Dr. Maisch GmbH, Ammerbuch-Entringen, Germany) and a flow rate of 250 nL/min were used to separate the peptides. 

As previously reported, an automatic data-dependent acquisition mode (DDA, shotgun) was used to acquire the MS data [32]. Raw MS files were analyzed by MaxQuant (MQ) version 1.5.0.30. A human FASTA (August 2016) was used for searching MS/MS spectra. The false discovery rate (FDR) set to 0.01 for peptide/protein identification was used in the MQ analysis. LFQ intensities were defined by comparing the signal intensity’s AUC (area under the curve) for any given peptide. Perseus software (version 1.6.0.7) was used to analyze proteomic data. To generate protein–protein interaction networks, STRING was also used. The KEGG pathway enrichment analysis tool in the PANTHER classification system was also applied to these networks. The DAVID (v2022q2) was also used to understand the biological meaning behind the changes of proteins between platelet-derived mEVs from CRC patients and HS.

### 2.7. Statistical Analysis

All data are reported as mean ± SD unless otherwise stated. GraphPad Prism Software (version 9.00 for macOS; GraphPad, San Diego, CA, USA) was used for statistical analyses. Two independent group means were compared by the Student’s t-test (two-tailed). One-way or two-way ANOVA (followed by Tukey or Sidak’s multiple comparison tests) compared more than two independent group means. *p*-values < 0.05 were considered statistically significant. A sample size of 10 individuals (matched for sex and age) in each group was considered appropriate for assessing changes in targeted gene expression in colon cancer cell lines by incubating with mEVs, based on previous data of platelet-HT29 cells cocultures [33]. For the proteomics characterization of mEVs from CRC patients vs. HS, a sample size of six individuals in each group was used based on previous studies [24].

## 3. Results

### 3.1. Individuals Enrolled in the Study

We enrolled 16 patients with CRC matched for sex and age with 16 HS (Table 1). The individuals did not present risk factors for CVD. Only one individual in each group presented hypertension. The patients with CRC were at initial diagnosis of nonmetastatic colorectal adenocarcinomas differently located (Table 1) and presented stage 1 (n = 2), 2a (n = 6), 2b (n = 1), 3a (n = 1), 3b (n = 4), and 3c (n = 2).

### 3.2. Count of Plasma mEVs and Generation of mEVs from Platelets of CRC Patients and HS

**Figure 1:**
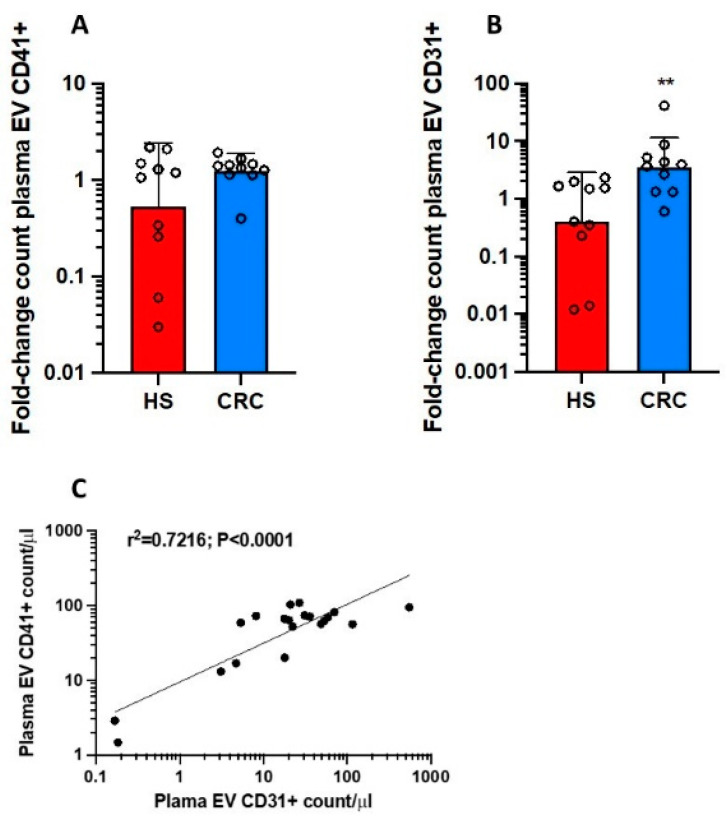
Changes in plasma count of medium-sized EV CD41^+^ or CD31^+^ collected from colorectal cancer (CRC) patients vs. healthy controls (HS). Plasma circulating mEVs positive for CD41 (platelet-derived) (**A**) or CD31 (vascular endothelial cell-derived) (**B**) were counted by flow-cytometry analysis, and fold-change in CRC patients vs. HS was assessed; plasma EVs were analyzed from 10 individuals with CRC and 10 healthy controls matched for sex and age; the values were not normally distributed and were transformed to logarithms, and all values are shown as scatter dot plots with mean + SD and analyzed by a two-tailed Student’s *t*-test, ** *p <* 0.01 vs. HS. (**C**) The log-transformed plasma count of mEV CD31^+^ and CD41^+^ fitted a linear regression model.

In 10 CRC patients and their HS matched for age (58.40 ± 12.29 and 57.40 ± 12.17 years, respectively, *p* = 0.857) and sex (females, 60%), we analyzed plasma mEVs using flow cytometry that revealed a nonsignificant change in the count of CD41^+^ mEVs (Figure 1A) while that of CD31^+^ mEVs was significantly enhanced in CRC patients vs. HS (Figure 1B). We found a relationship between CD31^+^ mEV count and mEV CD41^+^ count in the cohort of all individuals (Figure 1C). These findings may suggest vascular dysfunction in CRC patients associated with platelet activation.

**Figure 2:**
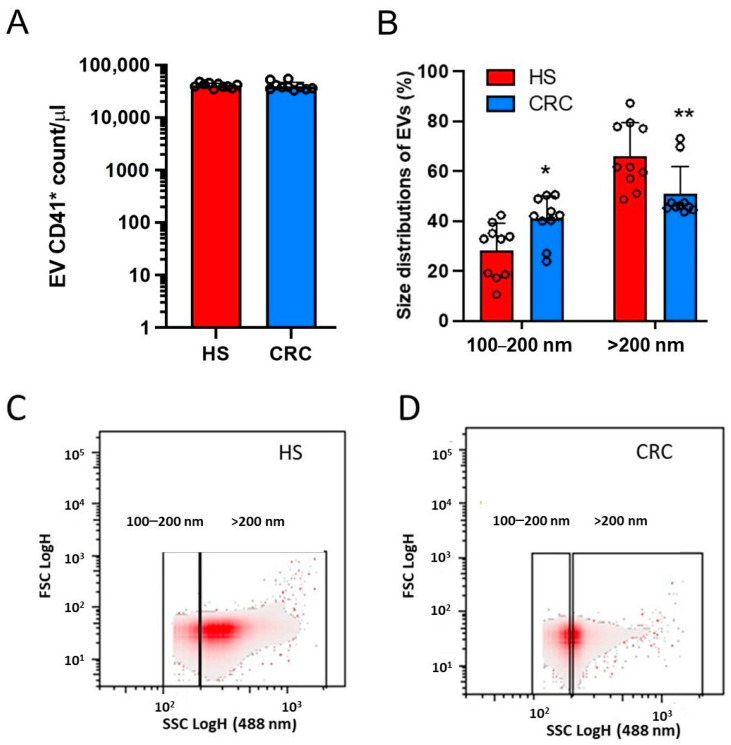
Count and features of mEVs released from thrombin-stimulated platelets of HS and CRC patients. Medium-sized EVs were obtained from washed platelets incubated with CaCl_2_ (2 mM) and thrombin (1U/mL) for 30 min at 37 °C in the water bath and then by centrifugation at 20,000× *g* for 40 min at 4 °C. (**A**) The count of mEV CD41^+^ in HS and CRC patients is shown and performed by flow cytometry (FACSVerse, BD Biosciences), as reported in the material and methods; the values were transformed to logarithms, and all values are shown as scatter dot plots with mean + SD. (**B**) The size distribution of EV suspensions obtained from thrombin-activated platelets of HS and CRC patients is reported as % EVs with a diameter of 100–200 nm and >200 nm; all values are shown as scatter dot plots with mean + SD and analyzed by two-way ANOVA and Sidak multiple comparison test; * *p <* 0.05, ** *p <* 0.01 vs. HS. (**C**,**D**) Density plots of forward scatter height (FSC-H) vs. side scatter height (SSC-H) of a typical EV suspension from HS and CRC patients, respectively; the rectangular describes the diameter range (nm) of EVs based on SSC (488 nm). FSC detects scatter along the path of the laser, and SSC measures scatter at a ninety-degree angle relative to the laser.

Washed platelets were isolated from the whole blood of the 10 CRC patients and matched HS. The cellular suspension contained > 98% of platelets [24]. Thrombin-stimulated washed platelets released mEVs that were collected. The count of platelet-derived mEVs, assessed by cytofluorimeter, was not significantly different in CRC and HS (Figure 2A) (40,328 ± 7473 vs. 40,975 ± 4266 count/μL, respectively, *p* = 0.8149). Using a cytofluorimeter, we studied the biophysical light scatter properties of mEVs obtained in the two groups. The density plot of side scatter (SSC) vs. forward scatter (FSC) of mEV suspension analysis showed that the size distribution was significantly different in CRC vs. HS (Figure 2B). A higher % of EVs with 100–200 nm diameter and a lower % of mEVs with >200 nm diameter were found in CRC vs. HS (Figure 2B–D).

### 3.3. Effects of Platelet-Derived mEVs on Gene Expression Markers of EMT in Colorectal Cancer Cells

EVs of both groups were studied for the capacity to alter the expression profile of marker genes of EMT in colorectal cancer cell lines (HCA7, HT29, and HCT116) derived from a primary adenocarcinoma of different Dukes stages (B, C, and D, respectively). We also studied the effects on the Caco2 cell line, originally derived from a colon carcinoma, but the Duke stage is unknown. The four colorectal cancer cell lines show differences in the genetic profile (Table S1). HCA7 cells are moderately differentiated [34], while HCT116 cells have a high metastatic potential [35]. HCT116 cells contain a hemizygous mutation in *MLH1* (i.e., a tumor suppressor gene involved in DNA mismatch repair), resulting in a truncated, non-functional protein [36]. 

**Figure 3:**
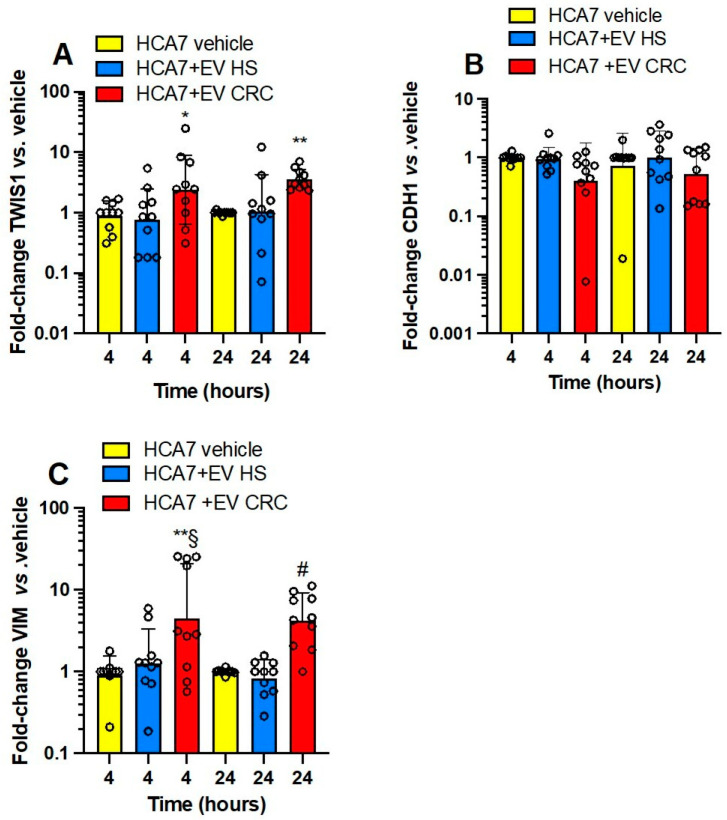
Effects of platelet-derived mEVs on the expression of gene markers of EMT in cocultures with the human colonic adenocarcinoma cell line HCA7. Cancer cells (0.5 × 10^6^) were incubated for 4 or 24 h with CRC or HS EVs (100 μg), and gene expression of *TWIST1* (**A**), *CDH1* (protein name E-cadherin) (**B**), and *VIM* (protein name vimentin) (**C**) was evaluated by qPCR and normalized to those of GAPDH and reported as fold-change vs. the gene expression detected in the cocultures of mEVs from HS with the cancer cells. The values were not normally distributed and were transformed to logarithms, and all values are shown as scatter dot plots with mean + SD, n = 10, and analyzed by one-way ANOVA followed by Tukey’s multiple comparisons test. (**A**) *TWIST1*, 4 h, * *p <* 0.05 vs. EV HS, 24 h, ** *p <* 0.01 vs. vehicle and EV HS; (**C**) *VIM*, 4 h ** *p <* 0.01 vs. vehicle, § *p <* 0.05 vs. EV HS, 24 h, # *p <* 0.01 vs. vehicle and EV HS.

In Figure 3A–C, the effects of platelet-derived mEVs from CRC and HS on the expression of the well-known EMT markers, *TWIST1* (twist family bHLH transcription factor 1), *CDH1* (E-cadherin), and *VIM* (vimentin) [37] in HCA7 cells are shown. Platelet-derived mEVs from CRC patients incubated with HCA7 cells for 4 and 24 h significantly induced *TWIST1* (Figure 3A) and *VIM* (Figure 3C), while *CDH1* was unaffected (Figure 3B). mEVs from HS did not significantly affect EMT gene markers.

**Figure 4:**
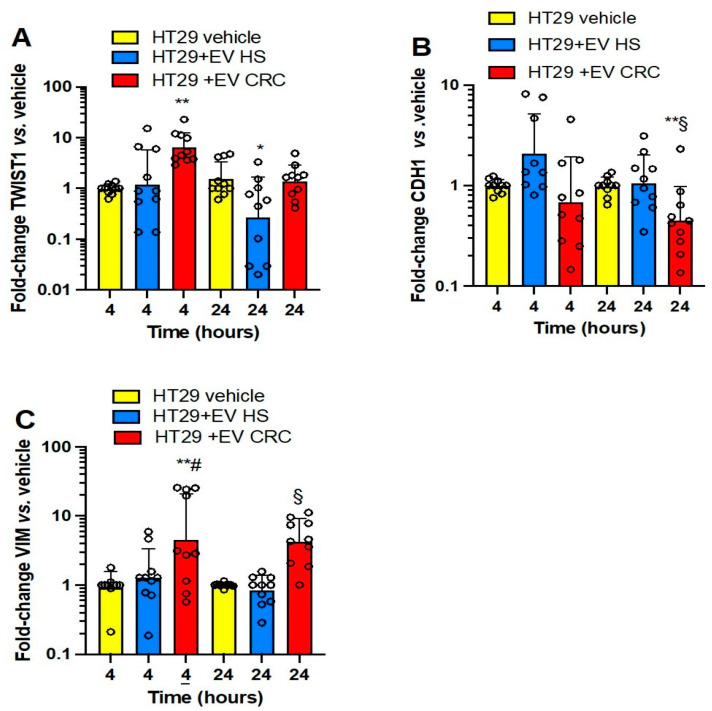
Effects of platelet-derived mEVs on the expression of gene markers of EMT in cocultures with the human colorectal adenocarcinoma cell line HT29. Cancer cells (0.5 × 10^6^) were incubated for 4 or 24 h with CRC or HS EVs (100 μg), and the gene expression of *TWIST1* (**A**), *CDH1* (protein name E-cadherin) (**B**), and *VIM* (protein name vimentin) (**C**) was evaluated by qPCR and normalized to those of *GAPDH* and reported as fold-change vs. the gene expression detected in the cocultures of mEV from HS with the cancer cells. The values were not normally distributed and were transformed to logarithms, and all values are shown as scatter dot plots with mean + SD, n = 10, and analyzed by one-way ANOVA followed by Tukey’s multiple comparisons test. (**A**) *TWIST1*, 4 h,** *p <* 0.01 vs. vehicle, 24 h, * *p <* 0.05 vs. vehicle and EV CRC; (B) *CDH1*, 24 h, ** *p <* 0.01 vs. EV HS, § *p <* 0.05 vs. vehicle; (**C**) *VIM*, 4 h, ** *p <* 0.01 vs. vehicle, # *p <* 0.05, 24 h, § *p <* 0.01 vs. vehicle and EV HS.

In HT29 cells (Figure 4A–C), platelet-derived mEVs from CRC significantly induced *TWIST1* (at 4 h) (Figure 4A) and *VIM* (at 4 and 24 h) (Figure 4C) vs. cancer cells cultured alone. Moreover, the downregulation of *CDH1* was detected at 24 h of incubation of cancer cells with mEV from CRC patients vs. mEV from HS and HT29 cells cultured alone (Figure 4B); mEVs from HS did not significantly affect EMT gene markers.

**Figure 5:**
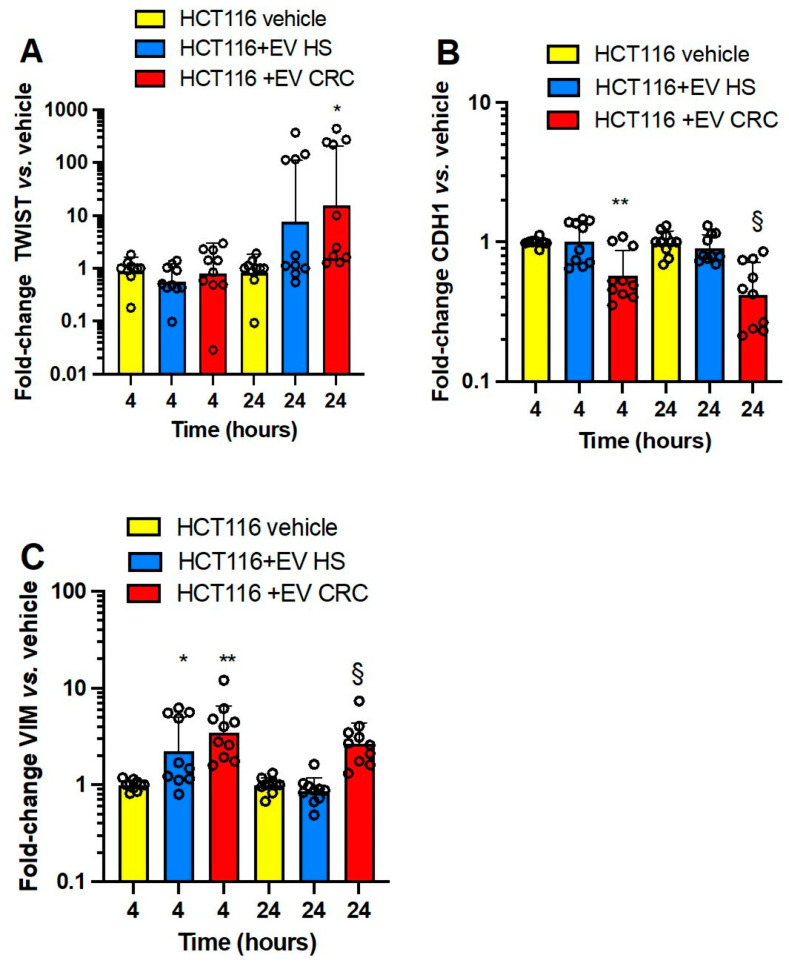
Effects of platelet-derived mEVs on the expression of gene markers of EMT in cocultures with the human colorectal carcinoma cell line HCT116. Cancer cells (0.5 × 10^6^) were incubated for 4 or 24 h with CRC or HS EVs (100 μg), and gene expression of *TWIST1* (**A**), *CDH1* (protein name E-cadherin) (**B**), and *VIM* (protein name vimentin) (**C**) was evaluated by qPCR and normalized to those of *GAPDH* and reported as fold-change vs. the gene expression detected in the cocultures of mEV from HS with the cancer cells. The values were not normally distributed and were transformed to logarithms, and all values are shown as scatter dot plots with mean + SD, n = 10, and analyzed by one-way ANOVA followed by Tukey’s multiple comparisons test. (**A**) *TWIST*, 24 h, * *p <* 0.05 vs. vehicle; (**B**) *CDH1* 4 h, ** *p <* 0.01 vs. vehicle and EV HS, 24 h, § *p <* 0.01 vs. vehicle and EC HS; (**C**) *VIM*, 4 h, * *p <* 0.05 vs. vehicle, ** *p <* 0.01 vs. vehicle, 24 h, § *p <* 0.01 vs. vehicle and EV HS.

Platelet-derived mEVs from CRC patients incubated with HCT116 cells significantly induced *TWIST1* (at 24 h) (Figure 5A) and *VIM* (at 4 and 24 h) (Figure 5C), and *CDH1* was significantly downregulated (at 4 and 24 h) (Figure 5B). 

**Figure 6:**
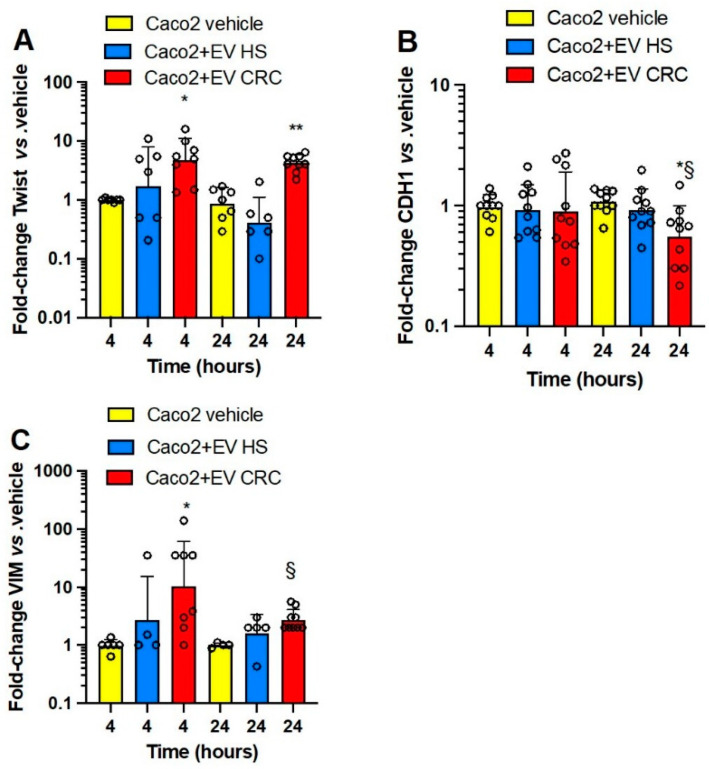
Effects of platelet-derived mEVs on the expression of gene markers of EMT in cocultures with the human colon adenocarcinoma cell line Caco2. Cancer cells (0.5 × 10^6^) were incubated for 4 or 24 h with CRC or HS EVs (100 μg), and the gene expression of *TWIST1* (**A**), *CDH1* (protein name E-cadherin) (**B**), and *VIM* (protein name vimentin) (**C**) was evaluated by qPCR and normalized to those of *GAPDH* and reported as fold-change vs. the gene expression detected in the cocultures of mEV from HS with the cancer cells. The values were not normally distributed and were transformed to logarithms, and all values are shown as scatter dot plots with mean + SD, n = 4–9, and analyzed by one-way ANOVA followed by Tukey’s multiple comparisons test. (**A**) *TWIST1*, 4 h, * *p <* 0.05 vs. vehicle, 24 h ** *p <* 0.01 vs. vehicle and EV HS; (**B**) *CDH1*, 24 h, * *p <* 0.05 vs. EV HS, § *p <* 0.01 vs. vehicle; (**C**) *VIM*, 4 h, * *p <* 0.05 vs. vehicle, 24 h, § *p <* 0.01 vs. vehicle.

In Caco2 cells (Figure 6A–C), platelet-derived mEVs from CRC-induced *TWIST1* (at 4 and 24 h) (Figure 6A) and *VIM* (at 4 and 24 h) (Figure 6C) while downregulating *CDH1* (at 24 h) (Figure 6B) vs. cancer cells cultured alone.

Altogether these results show that platelet-derived mEVs from CRC patients altered EMT marker gene expression in four human colorectal cancer cell lines. Differently from the results on the other three cancer cell lines, the *CDH1* expression of HCA7 cells was not significantly affected by mEVs from CRC patients. The effects of platelet-derived mEVs from HS were overall not statistically significant. 

### 3.4. Effects of Platelet-Derived mEVs on the Expression of PTGS2 and TXB_2_ Generation by Colorectal Cancer Cells

**Figure 7:**
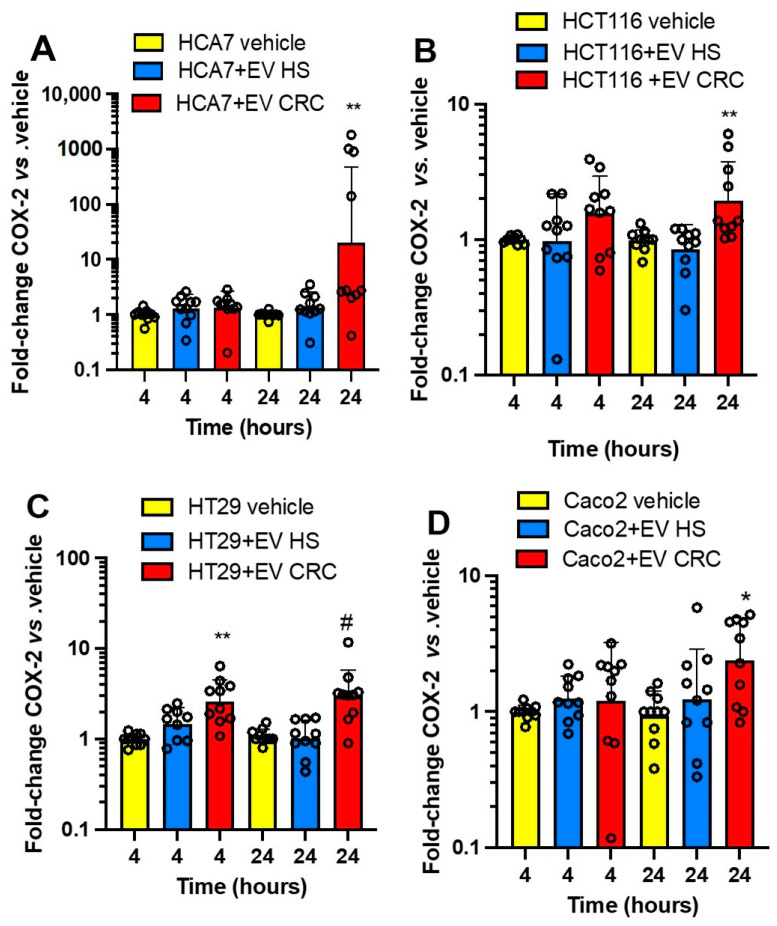
Effects of platelet-derived mEVs on the expression of COX-2 (*PTGS2*) in cocultures with cancer cells. Cancer cells (0.5 × 10^6^), (**A**) (HCA7 cells), (**B**) (HCT116 cells), (**C**) (HT29 cells), and (**D**) (Caco2 cells) were incubated for 4 and 24 h with EVs from CRC or HS individuals (100 μg), and the gene expression of *PTGS2* was evaluated by qPCR and normalized to that of *GAPDH* and reported as fold-change vs. the gene expression detected in the cocultures of mEV from HS with the cancer cells. The values were not normally distributed and were transformed to logarithms, and all values are shown as scatter dot plots with mean + SD, n = 10, and analyzed by one-way ANOVA followed by Tukey’s multiple comparisons test. (**A**) 24 h, ** *p <* 0.01 vs. vehicle and EV HS. (**B**) 24 h, ** *p <* 0.01 vs. vehicle and EV HS. (**C**) 4 h,** *p <* 0.01 vs. vehicle and EV HS; 24 h, # *p <* 0.01 vs. vehicle and EV HS. (**D**) 24 h, * *p <* 0.01 vs. vehicle and EV HC.

We aimed to verify the impact of the interaction of platelet-derived mEVs of CRC patients and HS on *PTGS2* expression in the four colorectal cancer cell lines (Figure 7A–D). At baseline, HCA7 cells showed a higher expression of *PTGS2* than that detected in the other four cancer cell lines, which presented comparable low gene expression (from 0.01 to 2% of that found in HCA7 cells) (Figure S1). The coculture of the four cancer cell lines with platelet-derived mEVs of CRC patients significantly induced *PTGS2*, mainly at 24 h (Figure 7A–D); in contrast, mEVs of HS did not significantly affect *PTGS2* gene expression.

**Figure 8:**
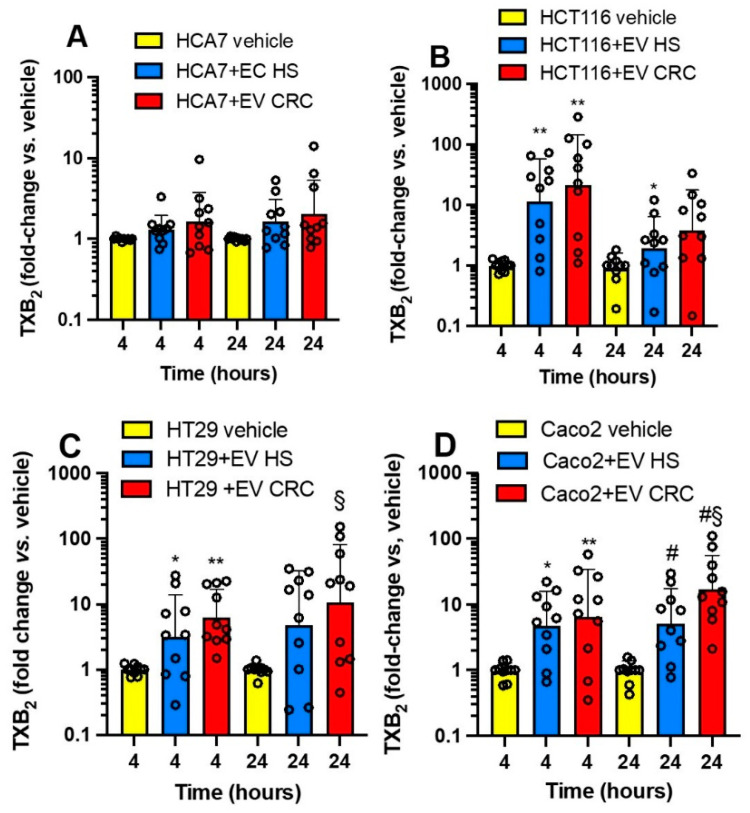
Effects of platelet-derived mEVs on TXB_2_ generation in cocultures with cancer cells. Cancer cells (0.5 × 10^6^), (**A**) (HCA7 cells), (**B**) (HCT116 cells), (**C**) (HT29 cells), (**D**) (Caco2 cells) were incubated for 4 and 24 h with EVs from CRC or HS individuals (100 μg), and TXB_2_ levels were assessed by immunoassay and reported as fold-change vs. values detected in the conditioned medium of cancer cells cultured alone. The values were not normally distributed and were transformed to logarithms, and all values are shown as scatter dot plots with mean + SD, n = 10, and analyzed by one-way ANOVA followed by Tukey’s multiple comparisons test. (**B**) 4 h, ** *p <* 0.01 vs. vehicle; 24 h, * *p <* 0.05 vs. vehicle. (**C**) 4 h, * *p <* 0.05, ** *p <* 0.01 vs. vehicle; 24 h, § *p <* 0.01 vs. vehicle. (**D**) 4 h, * *p <* 0.05, ** *p <* 0.01 vs. vehicle; 24 h, # *p <* 0.01 vs. vehicle, § *p <* 0.05 vs. EV HS.

Due to the important role of TXA_2_ in platelet function and cancer development [10,11], we assessed the generation of this prostanoid in CRC cells cultured alone vs. the cocultures with platelet-derived mEVs (Figure 8A–D). At baseline, HCA7 cells released significantly higher levels of TXB_2_ than the other cell lines (Figure S2). The other cell lines generated comparable TXB_2_ levels, from 1.5 to 2.5% of those detected in the conditioned medium of HCA7 cells. The coculture of HCA7 cells with platelet-derived EVs of CRC patients and HS did not influence TXB_2_ levels found at baseline (vehicle) (Figure 8A). In contrast, platelet-derived mEVs of CRC and HS enhanced the levels of TXB_2_ detected in the conditioned medium of the other cancer cell lines (Figure 8B–D). A difference in TXB_2_ generation by platelet-derived EVs of CRC vs. HS was only found in the cocultures with Caco2 at 24 h (Figure 8D). Platelet-derived mEVs from CRC patients and HS cultured alone for up to 24 h did not release detectable concentrations of TXB_2_.

Altogether, our results show that CRC cell lines have distinct abilities to generate TXA_2_ from endogenous arachidonic acid (AA) stores. HCA7 cells release the highest TXB_2_ levels at baseline, associated with the highest expression of *PTGS2* than the other colorectal cancer cell types. The incubation with mEVs from CRC or HS with HCA7 cells did not affect TXB_2_ generation. In contrast, platelet-derived mEVs from CRC patients and HS enhance TXB_2_ generation when cocultured with the other cancer cell lines. 

### 3.5. Proteomics Characterization of Platelet-Derived mEVs Obtained from CRC Patients and HS

We characterized the proteomics profile of mEVs released from thrombin-activated washed platelets of six CRC patients and six HS matched for age (72.00 ± 6.33 vs. 65.33 ± 7.82 years; *p* = 0.1354) and sex (females, 50%). Thus, proteins from mEVs were digested and analyzed by LC-MS/MS [24]. We identified 1429 proteins in platelet-derived mEVs (Table S2). 

**Figure 9:**
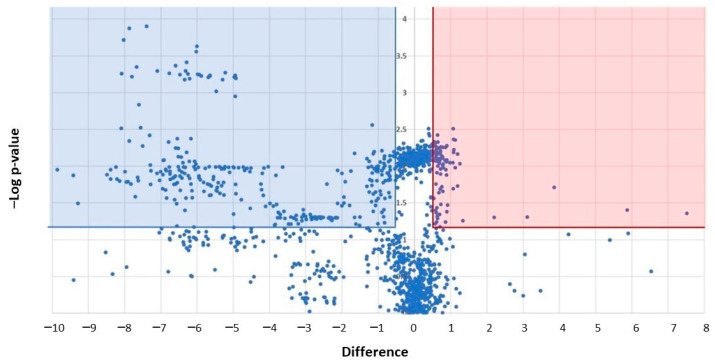
LC-MS/MS analysis of platelet-derived EVs obtained from CRC patients, and healthy controls revealed 208 significantly modulated proteins. Platelet-derived medium-size EVs were obtained from thrombin-stimulated washed platelets from six CRC patients and six healthy controls, matched for sex and age. MaxQuant software (version 1.5.0.30) was used to quantify EV proteins (after lysis, digestion, and purification) that LC-MS/MS identified. The 1429 proteins identified in the data set are shown in the Volcano plot. The log10 (*p*-value) and the “difference” values (CRC-controls) are reported on the *y*-axis and *x*-axis, respectively. The 208 significantly modulated proteins are shown: the proteins downregulated in CRC (blue square) and the upregulated in CRC (red square) *vs.* controls (*p* < 0.05, FDR 0.05, Difference > 0.4).

The colored dots of the Volcano plot show the 208 significantly modulated proteins between platelet-derived mEVs of CRC patients vs. HS (Figure 9). The blue squares represent the proteins downregulated in CRC, and the red squares are the proteins upregulated in CRC compared to HS (*p <* 0.05, FDR 0.05, Difference > 0.4). 

**Figure 10:**
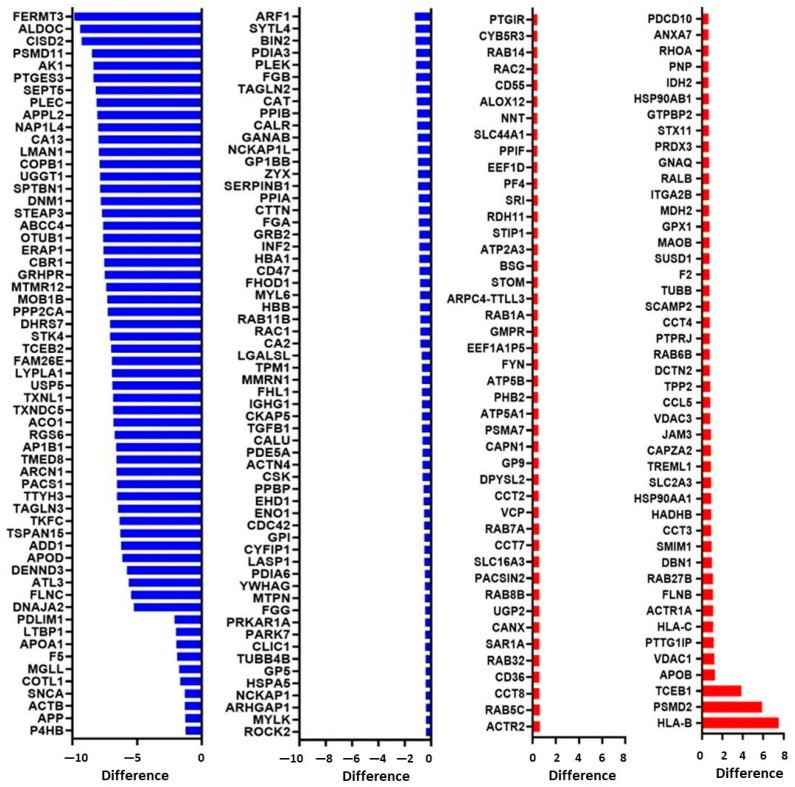
LC-MS/MS analysis of platelet-derived EVs obtained from CRC patients, and healthy controls revealed 208 significantly modulated proteins. The different levels of the 208 significantly modulated proteins are reported. The blue bars represent the 119 decreased proteins in CRC, and the red bars show the 89 significantly increased proteins in CRC compared to the control.

The list of the 208 proteins that were significantly modulated is shown in Figure 10. The blue bars represent the 119 downregulated proteins in platelet-derived mEVs of CRC patients vs. HS; the red bars are the 89 significantly upregulated proteins in CRC compared to HS (Figure 10). 

The list of names of modulated proteins is reported in Table S3. Thus, the most significantly altered proteins between platelet-derived mEVs of CRC patients compared to HS were decreased, while two proteins were highly increased: HLA-B class I and PSMD2. The human leukocyte antigen (HLA) system or complex is a group of related proteins encoded by the major histocompatibility complex (MHC) gene. PSMD2 is the proteasome 26S Subunit, Non-ATPase 2. 

**Figure 11:**
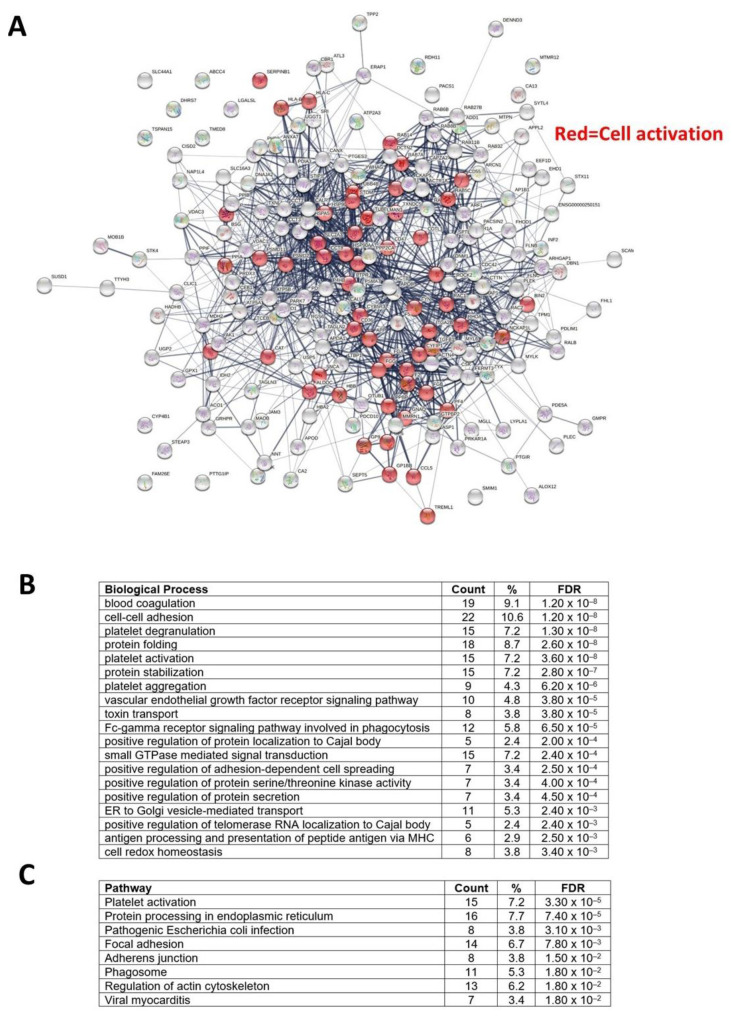
Network and pathway analysis of the significantly modulated proteins in platelet-derived EVs from CRC compared to controls (HS). (**A**) The proteins interaction network of the 208 identified proteins (showed as nodes in the network) modulated in CRC compared to controls (using STRING v11) is reported; proteins associated with “cell activation” are highlighted in red. (**B**) Biological processes of the significantly modulated proteins were obtained using DAVID Bioinformatics Resources. (**C**) Significantly modulated proteins were submitted to DAVID for Gene Ontology analysis, and the results for KEGG pathway analysis are reported.

The network analysis of the 208 proteins significantly modulated in CRC compared to HS demonstrated a strong protein network; the platelet activation network is highlighted in red (Figure 11A). Pathway analysis of the significantly modulated proteins was performed using DAVID Bioinformatics Resources. The most highly enriched biological processes are associated with coagulation and platelet activation (Figure 11B), and the top-ranked (by FDR) KEGG pathways involved platelet activation (Figure 11C). 

## 4. Discussion

Platelet-derived EVs circulate in plasma in physiological conditions and are mainly derived from megakaryocytes [22,38]. However, enhanced platelet-derived EVs have been detected in clinical conditions associated with platelet activation. CD41^+^ EVs can be released from activated platelets upon loss of cytoskeleton-membrane adhesion [39]. Platelet-derived EVs have emerged as a cellular system for cell–cell communication [21,22]. Platelet EVs act as a transport and delivery system for bioactive molecules, participating in hemostasis and thrombosis, inflammation, malignancy, infection, angiogenesis, and immunity [22]. Although many cell types can release EVs in blood circulation, platelet-derived EVs are particularly interesting because platelets can uptake and store many molecules (including growth and angiogenic factors and microRNAs) from plasma [40]. Thus, disease-educated platelets can release EVs with altered cargo compared to healthy conditions, impacting the development of important diseases, including cancer [21,41]. Moreover, the assessment of platelet-derived EV content has the promise of being a biomarker tool for the diagnosis of cancer and monitoring disease progression to metastasis [41]. 

We need more information on the biology of platelet-derived EVs in cancer, notably their ability to influence cancer cells by promoting pro-metastatic and thrombogenic phenotypes. This knowledge helps develop novel strategies for preventing cancer from spreading, representing the critical aspect of the death associated with cancer development. Here, we have characterized platelet-derived EVs obtained from nonmetastatic CRC patients at initial diagnosis for their capacity to reprogram the expression of EMT-related genes of four different human colorectal cancer cell lines characterized by distinct genetic features and metastatic potential. 

The acquisition of a mesenchymal-like phenotype by colorectal cancer cells occurs via EMT and can be triggered by autocrine and/or paracrine factors. EMT has been recognized as a relevant phenomenon in metastasis development by promoting cancer cell intravasation and extravasation [37]. EMT is associated with disruption of cell–cell adhesion and loss of E-cadherin. E-cadherin is a tumor suppressor that belongs to a family of calcium-dependent cell adhesion molecules. Various signaling molecules and transcription factors regulate the expression of E-cadherin [42]. Cellular E-cadherin loss occurs by multiple mechanisms, such as mutations, gene transcription repression by different transcription factors, including Twist1 and Slug, and gene promoter methylation [42].

Twist1 is a basic helix–loop–helix (bHLH) transcription factor significantly contributing to tumor metastasis, tumor initiation, and primary tumor growth. Overexpression of *TWIST1* induces EMT [43]. Another gene upregulated in EMT is *VIM*. Vimentin mediates cytoskeleton architecture in the mechanical homeostatic state and maintains focal adhesion maturation. Enhanced vimentin contributes to cytoskeleton organization and focal adhesion stability, thus allowing cancer cells to resist various stresses generated by the tumor microenvironment and promoting the increase of malignancy [44]. However, vimentin also regulates the expression of EMT-related genes [44]. We showed that platelet-derived EVs from CRC patients induced *TWIST1* and *VIM* in all the cancer cell lines studied, while platelet-derived EVs from HS did not. *CDH1* was not affected in HCA7 cells by platelet-derived EVs from CRC patients and HS. In contrast, HCT116, HT29, and Caco2 cells were associated with the downregulation of *CDH1* by the coculture with CRC platelet-derived EVs. Interestingly, HCT116 cells with high metastatic potential [35] showed downregulation of *CDH1* also at 4 h of incubation. These findings suggest that CRC platelet-derived mEVs impact colorectal cancer cells differently depending on their genotype/phenotype. Beta–catenin–Tcf complex has been reported to play a role in the induction of transcription factors, including *ZEB1* [45], *SNAI1* [46], and *TWIST1*, which can repress *CDH1* expression [47]. Differently from the other cell lines, HCA7 cells show normal APC and β-catenin [48]. Thus, it can be hypothesized that CRC platelet-derived mEVs influence EMT-related genes by activating the β-catenin–Tcf pathway. This effect could be more relevant in the presence of constitutive activation of β-catenin/Tcf-mediated transcription associated with *APC* and *CTNNB1* (gene name of β-catenin) mutations. 

We have previously found that the incubation of HT29 cells with platelets is associated with a time-dependent β-catenin translocation into the nucleus [49]. This effect was accompanied by the release from platelets of Wnt3a, a known activator of the β-catenin pathway [49]. Indeed, Steele et al. [50] found that Wnt3a is secreted from thrombin receptor agonist peptide-6 (TRAP)-activated platelets. Interestingly, Dervin et al. [51] characterized the proteome of human platelet exosomes and revealed that a population of these vesicles carry active WNT glycoproteins on their surface that can modulate WNT signaling activity in both endothelial and monocytic cells. However, we did not detect WNT proteins in mEVs released from thrombin-activated platelets of both CRC patients and HS. 

We have previously shown that the interaction of platelets with HT29 cells is associated with COX-2 induction and EMT-related genes and that selective inhibition of COX-2 activity mitigates EMT gene expression [6]. COX-2 expression requires a direct interaction between platelets and HT29 cells. However, platelet-soluble proteins, such as platelet-derived growth factor (PDGF), lead to mRNA stabilization of COX-2 via sodium–hydrogen exchanger (NHE)–PI3K (phosphoinositide 3-kinase)/protein kinase C (PKC)δ-dependent nucleo-cytoplasmic translocation of the mRNA-stabilizing protein HuR [6]. Thus, we verified whether platelet-derived mEVs, obtained from CRC patients and HS, induced COX-2 in the four colorectal cancer cell lines. By assessing the mRNA levels of *PTGS2* at 4 and 24 h, we verified the transcriptional vs. posttranscriptional regulation of COX-2 gene expression by mEVs. We found that platelet-derived EVs from CRC patients induced *PTGS2* in all four cancer cell lines at 24 h of cocultures, suggesting the contribution of a posttranscriptional mechanism. However, in HT29 cells, *PTGS2* was significantly induced at both 4 and 24 h by mEVs of CRC patients. Thus, EVs could also induce *PTGS2* via a transcription mechanism. Platelet-derived mEVs have been previously shown to induce de novo expression of COX-2, but not COX-1, in endothelial cells [52]. In the human monocytoid (U-937) cell, Barry et al. [30] have shown that platelet-derived mEVs induce COX-2 expression and prostanoid formation via mEV AA, which activates the membrane-linked signaling cascade (PKC, p42/p44 MAPK, p38 kinase, JNK1, c-Jun, and Elk-1). A specific study should address whether this pathway is involved in COX-2 induction by mEVs found in the present study. 

mEVs from HS did not significantly induce COX-2 in all four cancer cell lines. We have previously found that platelets from healthy donors induce COX-2 in HT29 cells [6]. The contribution of the direct platelet–cancer cell interaction in COX-2 overexpression can explain these different findings.

In various human malignancies, including colon cancer, the overexpression of COX-2 is associated with enhanced biosynthesis of PGE_2_, further sustained by the downregulation of hydroxyprostaglandin dehydrogenase 15-(NAD) (HPGD; also known as 15-PGDH) involved in the metabolism of PGE_2_ to less biological active products [7,8]. PGE_2_ promotes cancer by directly activating tumor epithelial cells, leading to proliferation, survival, migration, and invasion [7]. Moreover, PGE_2_ contributes to developing a proinflammatory tumor microenvironment, thus promoting tumor angiogenesis and the cancer cell immune escape [7].

We have previously shown that the induction of a mesenchymal-like phenotype in HT29 cells by the interaction with platelets translates into platelet activation and enhanced biosynthesis of TXA_2_ in vivo in NSG mice associated with lung metastasis [9]. TXA_2_ is a potent platelet activator and vasoconstrictor which plays a role in cancer development [10,11]. It is noteworthy that cancer-associated thrombosis is a major cause of mortality in cancer patients [53]. Thus, we explored the influence of platelet-derived mEVs on the biosynthesis of TXB_2_ in the coculture with colorectal cancer cells. Proteomics analysis of mEVs showed that platelet-derived mEVs contained COX-1 (prostaglandin G/H synthase-1) and the downstream TX-A synthase (Table S2); their levels were comparable in mEVs from CRC patients and HS. However, we did not find TXB_2_ production by mEVs of HS and CRC cultured alone. HCA7 cells generated higher TXB_2_ levels at baseline than the other cancer cell lines. These TXB_2_ levels generated by HCA7 cells were not affected by the interaction with mEVs. In contrast, a significant increase of TXB_2_ was detected in the conditioned medium of the other cancer cell lines (with low capacity to generate TXB_2_ at baseline) at 4 and 24 h of coculture with mEVs from CRC patients and HS. As found in endothelial cells by Pfister [54], cancer cells could provide AA to mEVs for generating TXB_2_. Alternatively, platelet mEVs could be uptaken by cancer cells and transfer COX-1 and TX-A synthase to cancer cells, thus enhancing TXA_2_ generation. Contursi et al. [33] have recently shown that in the coculture of platelets and HT29 cells, platelet-derived mEVs can transfer platelet-type 12-lipoxygenase(LOX) (protein name: arachidonate 12-lipoxygenase, 12S-type; gene name: *ALOX12*) to cancer cells that acquire the capacity to generate 12S-hydroxyeicosatetraenoic acid (HETE) mainly being esterified in membrane phospholipids. 

Platelet-derived mEVs from CRC and HS induce different signaling pathways in cancer cells, possibly due to differences in the molecule content. Thus, we compared the proteomics profile of mEVs released from activated platelets of CRC patients and HS. We found that 208 proteins were significantly modulated in platelet-derived EVs from CRC patients vs. HS, with 119 decreased and 89 increased. Two proteins were highly expressed in platelet-derived mEVs of CRC patients vs. HS, i.e., HLA-B class I and PSMD2. The human leukocyte antigen (HLA) system or complex is a group of related proteins encoded by the MHC gene. It has been reported that HLA I expression is a valuable parameter in identifying young platelets [55]. Young platelets are more reactive than the older population and are enhanced in response to the high turnover of the circulating platelet pool [56]. Thus, our finding of enhanced HLA I levels in platelet-derived mEVs of CRC patients vs. HS suggests increased platelet activation in CRC patients vs. HS. 

PSMD2 is the proteasome 26S Subunit, Non-ATPase 2. The ubiquitin-proteasome system (UPS) plays a crucial role in maintaining cellular protein homeostasis by degrading non-functional self-, foreign-, or short-lived regulatory proteins [57]. Peptide fragments released by platelet proteasomes can bind to MHC class I, making it likely that platelets can activate epitope-specific cytotoxic T lymphocytes (CTLs) [57]. Thus, enhanced levels of HLA-B class I and PSMD2 detected in platelet-derived mEVs of CRC patients suggest platelet-derived mEVs’ role as critical modulators of the immune response in CRC. Notably, the downregulation of proteins in CRC mEVs vs. HS was associated with enhanced PSMD2.

We have found enhanced protein levels of platelet-type 12-LOX (Figure 10 and Table S3) in platelet-derived mEVs from CRC patients vs. HS. This protein catalyzes the biosynthesis of 12S-HETE, known to contribute to the invasion and metastasis of tumors by different mechanisms, including regulating EMT-related gene expression [33,58]. Moreover, we have previously found that circulating mEVs collected from patients with colorectal adenomas/adenocarcinomas contain 12-LOX protein [33]. 12-LOX expressed in platelet-rich plasma (PRP) is a promising diagnostic and prognostic prostate cancer biomarker [59,60]. 

Using the DAVID bioinformatics resource system, we explored the biological meaning behind the downregulation of the 119 proteins in mEVs from CRC patients vs. HS by assessing information generated by the KEGG-pathway database. The more relevant pathways downregulated were focal adhesion, regulation of actin cytoskeleton, and tight junction (Figure S3A–C). It is known that EV generation requires cytoskeletal re-organization in the origin cell [61]. Several studies have reported the role of the proteolysis of cytoskeletal proteins by calpain in remodeling the submembranous cytoskeleton involved in platelet microvesiculation [39]. Lower mEV content of cytoskeletal proteins may be related to enhanced platelet activation and proteolytic activity in CRC platelets. Although we did not find a significantly higher number of circulating mEVs CD41^+^ in CRC patients vs. HS, a higher number of CD31^+^ mEVs was detected, which was linearly correlated with mEVs CD41^+^ count. These findings suggest vascular dysfunction in CRC patients associated with platelet activation. The bioinformatics data also support it; in platelet-derived mEVs of CRC vs. HS, platelet activation is one of the most relevant pathways upregulated. The cluster of proteins upregulated in this pathway are reported in Figure S4. 

Changes in the protein content of EVs from CRC patients vs. HS were associated with a different size (diameter) distribution of EVs of the two groups. Interestingly, EVs generated from thrombin-activated platelets of CRC patients were constituted by a higher % of 100–200 nm vesicles and a lower % of >200 nm vesicles than HS. It has been reported that diverse sizes of platelet-derived EVs contain different protein components [62]. Thus, further studies are necessary to clarify the determinants involved in the different features of platelet-derived EVs associated with CRC.

## 5. Conclusions

Our findings suggest that the proteome of thrombin-stimulated mEVs is modulated in CRC, and its evaluation may represent a noninvasive tool to discriminate patients from healthy subjects. More than the circulating number is the phenotype of the EVs to provide information on patient clinical features helpful in cancer diagnosis. Moreover, we show that the characterization of the expression of promalignant genes and prothrombotic phenotypes in cancer cells by the crosstalk with platelet mEVs could provide prognostic information on cancer development on an individual basis, which could help in developing an appropriate treatment plan.

## Figures and Tables

**Table 1:** Demographic and clinical features of colorectal cancer (CRC) patients and healthy controls (HS).

	HS	CRC
Number, n	16	16
Sex, F, n	9	9
Age, years, mean ± SD	60.38 ± 11.18	63.50 ± 12.29
BMI, mean ± SD	22.61 ± 3.00	24.29 ± 5.24
Hypertension, n	1	1
Diagnosis		
Adenocarcinoma ascending colon, n	-	5
Adenocarcinoma descending colon, n	-	5
Adenocarcinoma rectosigmoid junction, n	-	1
Adenocarcinoma rectum, n	-	4
Adenocarcinoma trasverse colon, n	-	1

## Data Availability

The raw data supporting the conclusion of this article will be made available by the authors, without undue reservation. The mass spectrometry proteomics data have been deposited to the ProteomeXchange Consortium via the PRIDE [65] partner repository with the dataset identifier PXD038674.

## Supplementary Materials

The following supporting information can be downloaded at: https://www.mdpi.com/article/10.3390/cancers15020350/s1. Figure S1: Gene expression of PTGS2 in different cancer cell lines vs. GAPDH at baseline, by qPCR. Figure S2: Baseline TXB_2_ generated by cancer cells at 4 and 24 h of culture. Figure S3: Downregulated pathways in mEVs from CRC patients vs. HS. Figure S4: Upregulated pathways in mEVs from CRC patients vs. HS. Table S1: Name of the different cell lines used in this study, the Duke’s stage, the genetic profile and COX-isozyme expression [63,64]. Table S2: List of identified proteins in platelet-derived mEVs. Table S3. List of modulated proteins in mEVs of CRC patients vs. HS.
