# Supplementary material for: Tumor-Educated Platelet Extracellular Vesicles: Proteomic Profiling and Crosstalk with Colorectal Cancer Cells
Source: Cancers (Basel). 2023 Jan 5;15(2):350. doi: 10.3390/cancers15020350 (PMC9856452; doi:10.3390/cancers15020350)
Supplement: Supplementary file 1 [file cancers-15-00350-s001.zip › cancers-2125101-supplementary.pdf]

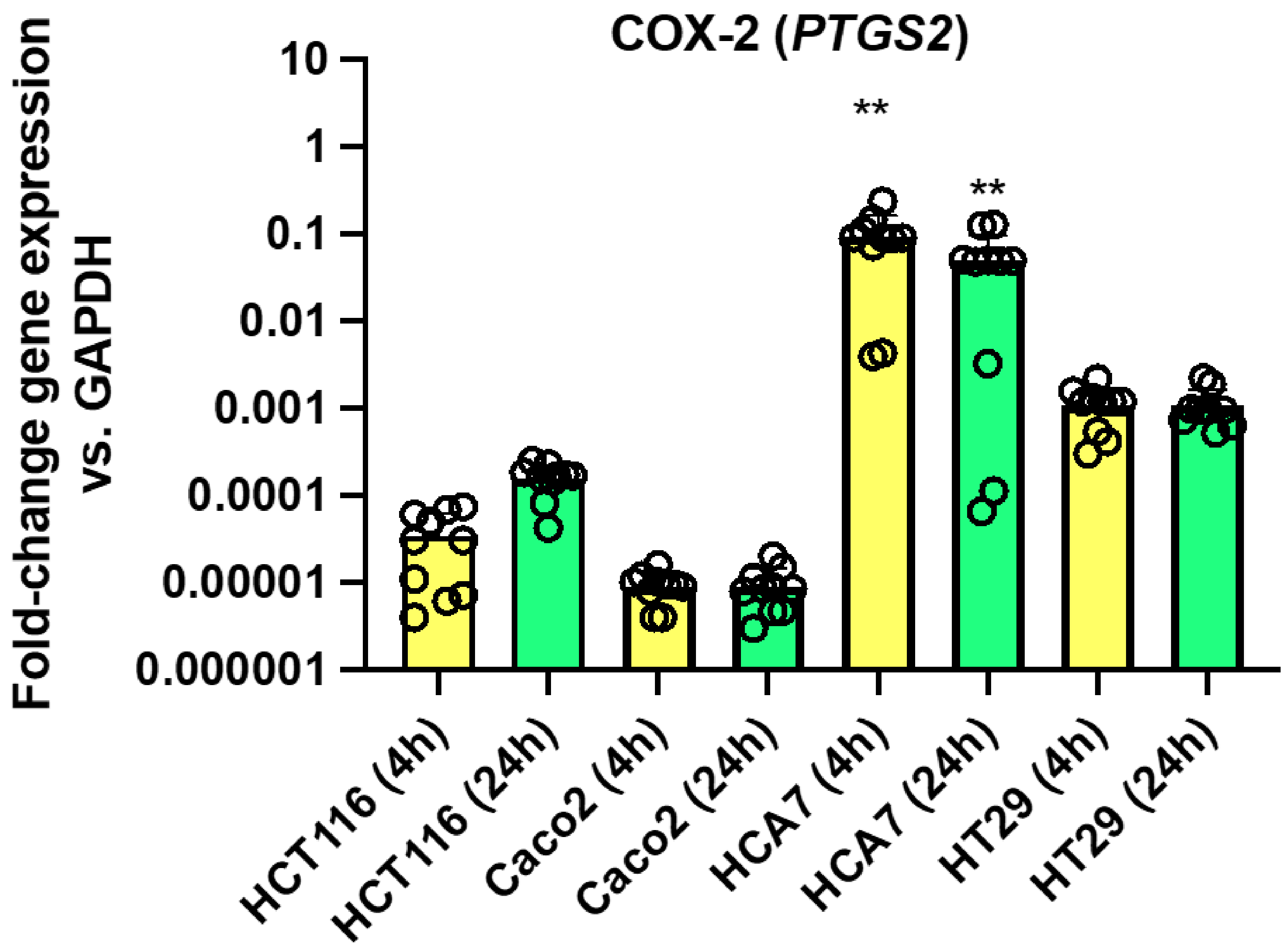

Figure S1. Gene expression of PTGS2 in different cancer cell lines *vs.* GAPDH at baseline, by qPCR. The values were not normally distributed and were transformed into logarithms. All values are shown as scatter dot plots with mean+SD, n=10, and analyzed by one-way ANOVA followed by Tukey's multiple comparisons test. **P<0.01 vs. all other conditions.

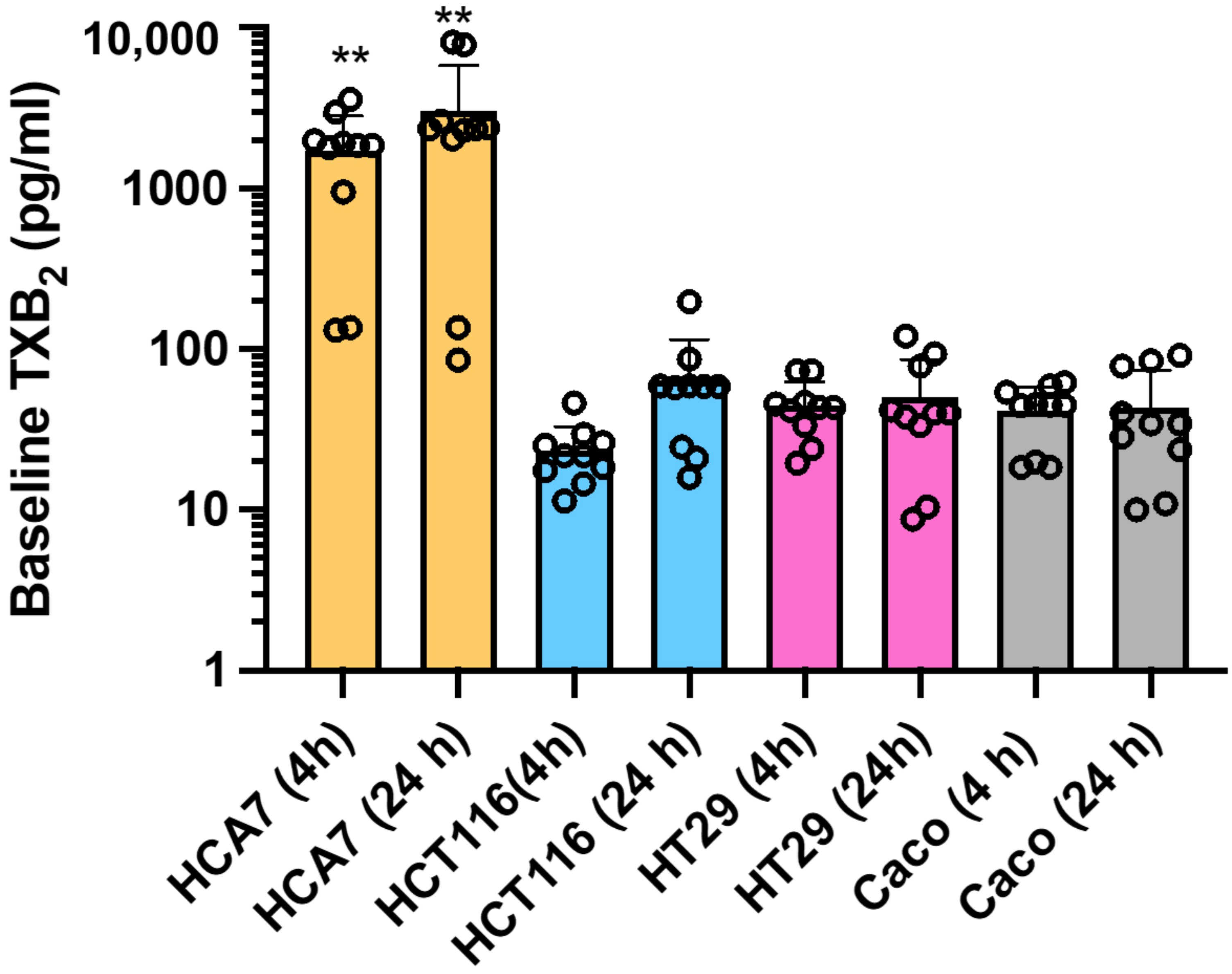

Figure S2. Baseline TXB_2 generated by cancer cells at 4 and 24 h of culture. Cancer cells (0.5×10^6) were cultured alone for 4 or 24 h and TXB_2 levels were assessed by immunoassay. The values were not normally distributed and were transformed into logarithms. All values are shown as scatter dot plots with mean+SD, n=10, and analyzed by one-way ANOVA followed by Tukey's multiple comparisons test. **P<0.01 vs. all other conditions.

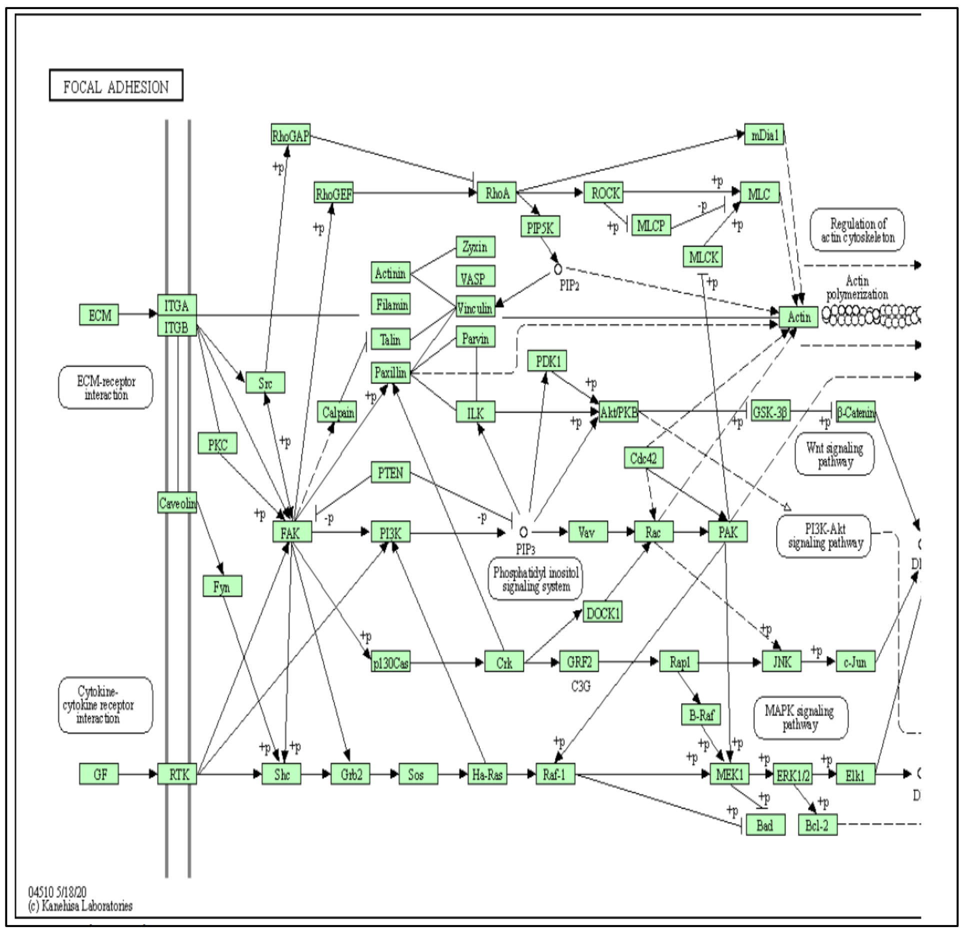

- Rac family small GTPase 1(RAC1)
- Rho associated coiled-coil containing protein kinase 2(ROCK2)
- actinin alpha 4(ACTN4)
- actin beta(ACTB)
- cell division cycle 42(CDC42)
- filamin C(FLNC)
- growth factor receptor bound protein 2(GRB2)
- myosin light chain kinase(MYLK)
- zyxin(ZYX)

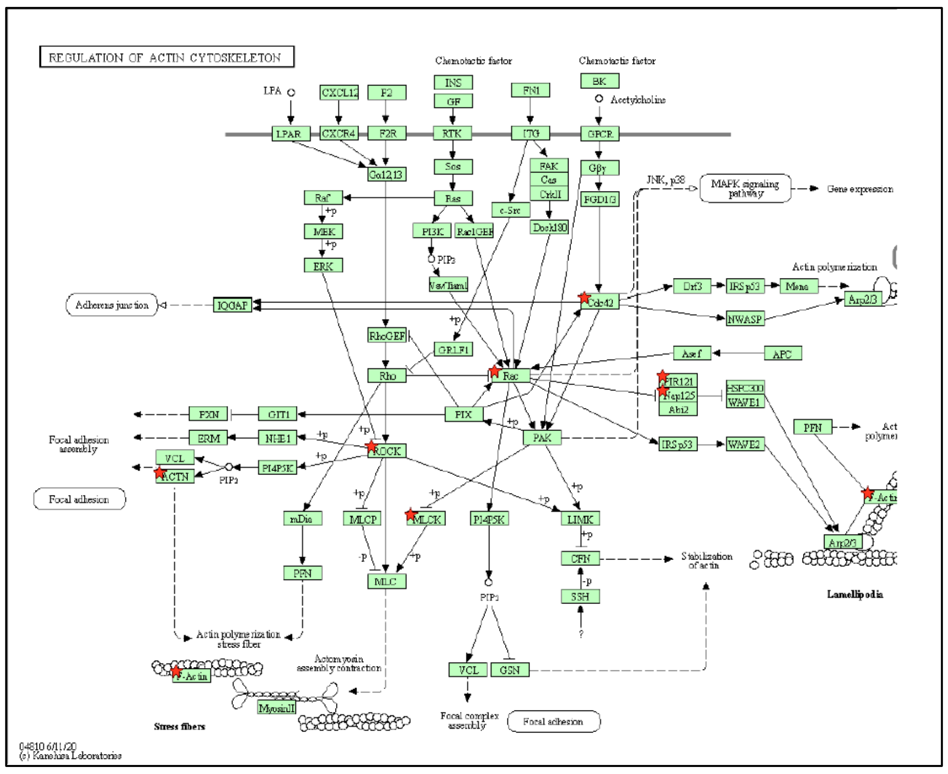

- NCK associated protein 1 like(NCKAP1L)
- NCK associated protein 1(NCKAP1)
- Rac family small GTPase 1(RAC1)
- Rho associated coiled-coil containing protein kinase 2(ROCK2)
- actin beta(ACTB)
- actinin alpha 4(ACTN4)
- cell division cycle 42(CDC42)
- cytoplasmic FMR1 interacting protein 1(CYFIP1)
- myosin light chain kinase(MYLK)

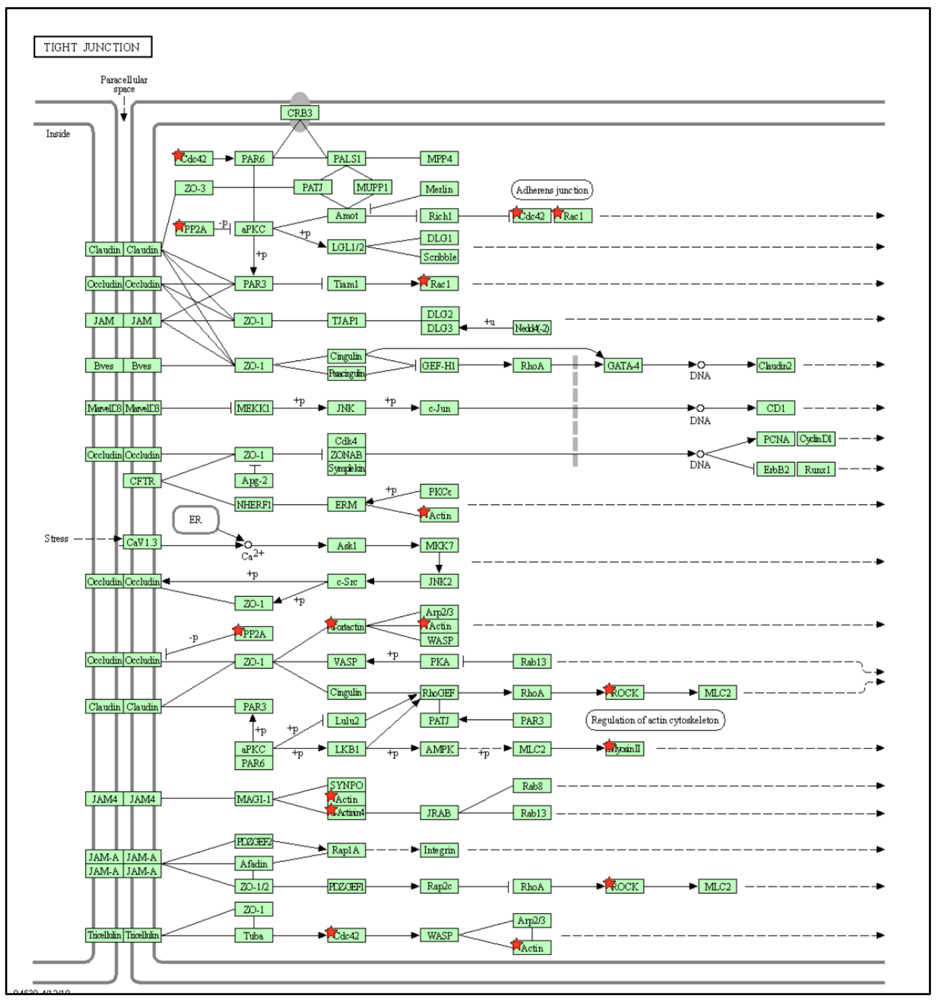

- Rac family small GTPase 1(RAC1)
- Rho associated coiled-coil containing protein kinase 2(ROCK2)
- actin beta(ACTB)
- actinin alpha 4(ACTN4)
- cell division cycle 42(CDC42)
- cortactin(CTTN)
- myosin light chain 6(MYL6)
- protein phosphatase 2 catalytic subunit alpha(PPP2CA)

Figure S3. Downregulated pathways in mEVs from CRC patients vs. HS

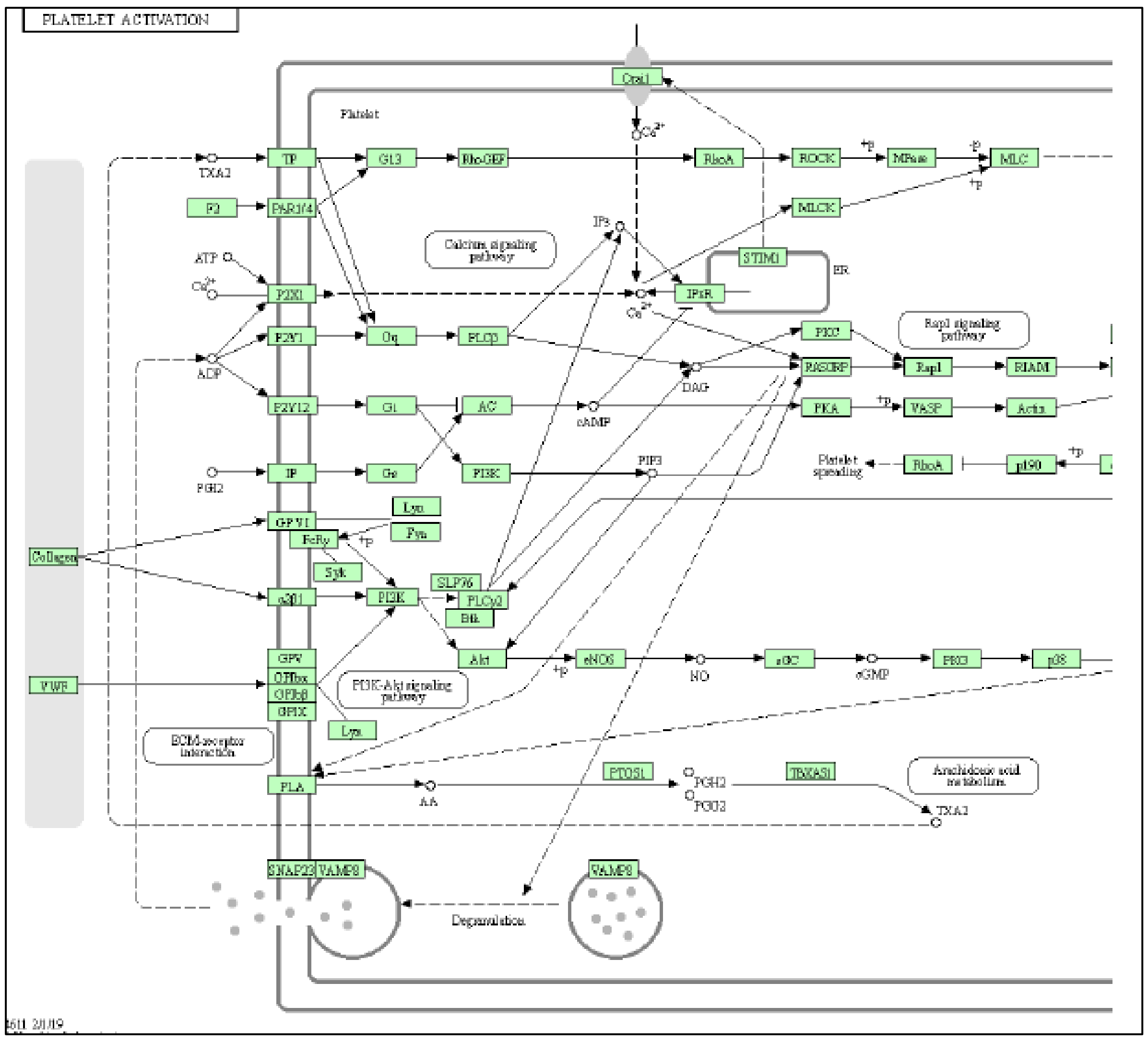

- FYN proto-oncogene, Src family tyrosine kinase(FYN))
- G protein subunit alpha q(GNAQ)
- coagulation factor II, thrombin(F2)
- glycoprotein IX platelet(GP9),
- integrin subunit alpha 2b(ITGA2B),
- prostaglandin I2 receptor(PTGIR)
- Arachidonate 12-lipoxygenase, 12S-type (ALOX12)
- ras homolog family member A(RHOA)

Figure S4. Upregulated pathways in mEVs from CRC patients vs. HS

Table S1. Name of the different cell lines used in this study, the Duke's stage, the genetic profile and COX-isozyme expression.

cell line	TGF-β receptors	PDGF receptors	p53	β-catenin	APC	MLH1	COX-2	COX-1	DUKE STAGE
HT29	mutated	√	mutated	not degradable	truncated	wt	√	√	C
HCA7	√	√	mutated	wt	wt	absent	√	nd	B
HCT116	mutated	√	wt	mutated	wt	mutated	nd	√	D
Caco2	√	√	mutated	mutated	mutated	wt	√	√	not known

COX-1 and COX-2 expression data from [64]. MLH1 data from [36, 65]

Table S2. List of identified proteins in platelet-derived mEVs

Gene names	Protein IDs	Protein names	Peptides	Sequence coverage [%]	Unique sequence coverage [%]	Mol. weight [kDa]	Q-value	Score
ZYX	Q15942	Zyxin	23	54,4	54,4	61,277	0	284,02
ZNF366	Q8N895	Zinc finger protein 366	1	1,3	1,3	85,107	1	-2
ZMPSTE24	O75844	CAAX prenyl protease 1 homolog	2	3,4	3,4	54,812	0,0073046	1,7797
ZFR	Q96KR1	Zinc finger RNA-binding protein	1	1,2	1,2	117,01	1	-2
ZFAND2B	B8ZZ56	AN1-type zinc finger protein 2B	2	17,2	17,2	16,233	0	3,1807
ZC3HAV1L	Q96H79	Zinc finger CCCH-type antiviral	1	4,3	4,3	32,962	0	2,9665
ZC3HAV1	Q7Z2W4	Zinc finger CCCH-type antiviral	2	3,4	3,4	101,43	0	3,3888
YWHAZ	P63104	14-3-3 protein zeta/delta	25	80,8	69	27,745	0	323,31
YWHAQ	P27348	14-3-3 protein theta	14	46,1	34,7	27,764	0	81,15
YWHAH	Q04917	14-3-3 protein eta	20	58,5	54,5	28,218	0	241,71
YWHAG	P61981	14-3-3 protein gamma	19	77,3	67,6	28,302	0	167,08
YWHAE	P62258	14-3-3 protein epsilon	23	75,3	72,2	29,174	0	200,35
YWHAB	P31946	14-3-3 protein beta/alpha	13	57,7	31,7	28,082	0	116,73
YKT6	O15498	Synaptobrevin homolog YKT6	2	15,2	15,2	22,417	0,0031008	2,3693
YES1	J3QRU1	Non-specific protein-tyrosine	13	22,8	6,9	61,386	0	10,76
YARS	P54577	Tyrosine--tRNA ligase,	15	35,2	35,2	59,143	0	25,818
XYLT2	Q9H1B5	Xylosyltransferase 2	5	7,3	7,3	96,766	0	9,6597
XPO7	E7ESC6	Exportin-7	6	5,9	5,9	124,07	0	7,223
XPO1	O14980	Exportin-1	6	6,5	6,5	123,38	0	8,6047
XPNPEP1	Q5T6H7	Xaa-Pro aminopeptidase 1	6	16,1	16,1	62,138	0	26,778
WIPF1	O43516	WAS/WASL-interacting protein family member 1	5	15,5	15,5	51,258	0	10,479
WDR44	H7BY83	WD repeat-containing protein 44	16	24,9	24,9	89,847	0	37,08
WDR1	O75083	WD repeat-containing protein 1	39	74,1	74,1	66,193	0	323,31
WASF3	Q9UPY6	Wiskott-Aldrich syndrome	3	7,8	7,8	55,292	0	3,2878
WASF2	Q9Y6W5	Wiskott-Aldrich syndrome	7	16,3	16,3	54,283	0	51,327
WASF1	Q92558	Wiskott-Aldrich syndrome	2	5,2	5,2	61,651	0,0031177	2,4225
WAS	P42768	Wiskott-Aldrich syndrome	10	27,1	27,1	52,912	0	33,876
WARS	G3V3Y5	Tryptophan--tRNA ligase,	3	18,1	18,1	25,798	0,0023566	2,4835

VWF	P04275	von Willebrand factor	17	8	8	309,26	0	46,216
VTN	P04004	Vitronectin	9	25,3	25,3	54,305	0	35,918
VTA1	A0A087WY55	Vacuolar protein sorting-associated protein VTA1 homolog	2	8,6	8,6	31,102	0	4,6264
VPS4B	O75351	Vacuolar protein sorting-	3	8,6	8,6	49,301	0	7,7714
VPS45	A0A087WU65	Vacuolar protein sorting-	1	3,1	3,1	50,736	0,0045317	2,1369
VPS35	Q96QK1	Vacuolar protein sorting-	8	12,2	12,2	91,706	0	18,728
VPS33B	F5H008	Vacuolar protein sorting-	1	2	2	67,605	0,0067064	1,9205
VPS29	F8VXU5	Vacuolar protein sorting-	1	4,7	4,7	23,98	0,0093057	1,6439
VPS28	E9PR04	Vacuolar protein sorting-associated protein 28 homolog	2	69,8	69,8	5,9968	0	5,3845
VPS26B	Q4G0F5	Vacuolar protein sorting-	2	6	6	39,154	0	3,4516
VPS13C	Q709C8	Vacuolar protein sorting-	3	1,1	1,1	422,39	0,0023678	2,5277
VIM	A0A1B0GTT5	Vimentin	2	17,4	12,8	16,425	0	40,64
VDAC3	Q9Y277	Voltage-dependent anion-selective channel protein 3	13	58,7	58,7	30,658	0	95,214
VDAC2	A0A0A0MR02	Voltage-dependent anion-selective channel protein 2	10	40,4	40,4	30,348	0	45,986
VDAC1	P21796	Voltage-dependent anion-selective channel protein 1	17	72,8	72,8	30,772	0	55,277
VCP	P55072	Transitional endoplasmic	40	58,2	58,2	89,321	0	259,59
VCL	P18206	Vinculin	93	74,5	74,5	123,8	0	323,31
VAV1	F5H5P4	Proto-oncogene vav	2	2,7	2,7	91,729	0	2,748
VAT1	Q99536	Synaptic vesicle membrane	2	6,4	6,4	41,92	0	3,866
VASP	P50552	Vasodilator-stimulated	20	49,5	49,5	39,829	0	170,29
VAPA	Q9P0L0	Vesicle-associated membrane protein-associated protein A	4	23,3	23,3	27,893	0	11,634
VAMP8	Q9BV40	Vesicle-associated membrane	4	32	32	11,438	0	9,1179
VAMP7	P51809	Vesicle-associated membrane	5	21,8	21,8	24,935	0	12,751
VAMP3	Q15836	Vesicle-associated membrane	3	53	53	11,309	0	10,851
UTS2	Q5H8X8	Urotensin-2	1	5,8	5,8	16,276	0,0052592	2,0905
USP5	P45974	Ubiquitin carboxyl-terminal	7	11,8	11,8	95,785	0	16,924
USP47	Q96K76	Ubiquitin carboxyl-terminal	1	1,2	1,2	157,31	0,0073529	1,8223

USP14	A6NJA2	Ubiquitin carboxyl-terminal	6	16,5	16,5	51,086	0	13,609
USO1	O60763	General vesicular transport factor	2	2	2	107,89	0,0067265	1,9506
UROD	P06132	Uroporphyrinogen decarboxylase	8	32,4	32,4	40,786	0	22,557
UQCRQ	O14949	Cytochrome b-c1 complex	2	25,6	25,6	9,9062	0	3,4055
UQCRH	P07919	Cytochrome b-c1 complex	2	28,6	28,6	10,739	0	4,2098
UQCRFS1	P47985	Cytochrome b-c1 complex subunit Rieske, mitochondrial	1	3,3	3,3	29,668	0	2,7335
UQCRC2	P22695	Cytochrome b-c1 complex	10	32	32	48,442	0	42,189
UQCRC1	P31930	Cytochrome b-c1 complex	12	39,8	39,8	52,645	0	38,871
UQCRB	P14927	Cytochrome b-c1 complex	3	36,9	36,9	13,53	0	6,3492
UNC45A	A0A1W2PNX8	Protein unc-45 homolog A	7	8,7	8,7	118,39	0	15,121
UNC13D	K7EQ37		12	40,4	3,4	30,68	0,0073584	1,8242
UNC13D	Q70J99	Protein unc-13 homolog D	37	38,6	29,5	123,28	0	188,9
UMPS	F2Z3P2	Uridine 5-monophosphate	1	21,1	21,1	6,1502	0,0073099	1,7806
UGP2	A0A087WYS1	UTP--glucose-1-phosphate	14	32,9	32,9	56,967	0	37,201
UGGT1	Q9NYU2	UDP-glucose:glycoprotein	11	8,1	8,1	177,19	0	14,47
UFC1	Q9Y3C8	Ubiquitin-fold modifier-	1	7,8	7,8	19,458	0	2,8629
UBR4	A0A0A0MSW0	E3 ubiquitin-protein ligase UBR4	2	1,2	1,2	225,01	0,00080257	2,6925
UBE2O	Q9C0C9	E2/E3 hybrid ubiquitin-protein	11	12,8	12,8	141,29	0	19,078
UBE2N	P61088	Ubiquitin-conjugating enzyme E2	5	39,5	27,6	17,138	0	10,671
UBE2L3	P68036	Ubiquitin-conjugating enzyme E2	4	41,6	41,6	17,861	0	13,815
UBE2D3	D6RAW0	Ubiquitin-conjugating enzyme E2	2	40,3	40,3	8,2133	0	3,8835
UBC	P0CG48	Polyubiquitin-C	2	32,8	21	77,038	0	44,478
UBASH3B	Q8TF42	Ubiquitin-associated and SH3 domain-containing protein B	21	39	39	72,695	0	128
UBA7	P41226	Ubiquitin-like modifier-activating	17	23,7	23,7	111,69	0	53,102
UBA6	A0AVT1	Ubiquitin-like modifier-activating	3	3,5	3,5	117,97	0	7,3564
UBA1	P22314	Ubiquitin-like modifier-activating	21	25,7	25,7	117,85	0	90,648
TYMP	P19971	Thymidine phosphorylase	15	43,2	43,2	49,955	0	46,774
TXNRD1	E9PIR7	Thioredoxin reductase 1,	2	5,4	5,4	53,166	0	3,7955
TXNL1	O43396	Thioredoxin-like protein 1	3	17	17	32,251	0	5,163
TXNDC5	Q8NBS9	Thioredoxin domain-containing	4	11,1	11,1	47,628	0	16,317
TXN	P10599	Thioredoxin	4	42,9	42,9	11,737	0	12,934

TWF2	Q6IBS0	Twinfilin-2	17	58,7	58,7	39,548	0	172,56
TUFM	P49411	Elongation factor Tu,	13	36,9	36,9	49,541	0	39,065
TUBB8	Q3ZCM7	Tubulin beta-8 chain	11	24,5	7,4	49,775	0	51,363
TUBB6	Q9BUF5	Tubulin beta-6 chain	12	30,5	4	49,857	0	27,777
TUBB4B	P68371	Tubulin beta-4B chain	28	75,7	2,7	49,83	0	323,31
TUBB4A	M0QYM7		6	69,4	8,8	17,763	1	-2
TUBB4A	P04350	Tubulin beta-4A chain	21	63,5	4,1	49,585	0	5,6491
TUBB2B	Q9BVA1	Tubulin beta-2B chain	22	57,3	0	49,953	1	-2
TUBB1	Q9H4B7	Tubulin beta-1 chain	31	87,6	79,2	50,326	0	323,31
TUBB	Q5JP53	Tubulin beta chain	26	74,6	16,9	47,766	0	133,65
TUBAL3	A6NHL2	Tubulin alpha chain-like 3	5	9,6	3,8	49,908	0	15,199
TUBA8	C9J2C0	Tubulin alpha-8 chain	25	56,5	22,9	51,984	0	82,966
TUBA4A	P68366	Tubulin alpha-4A chain	31	65,2	19,4	49,924	0	45,597
TUBA1C	F5H5D3	Tubulin alpha-1C chain	31	60,5	7,9	57,73	0	45,084
TUBA1B	P68363	Tubulin alpha-1B chain	33	74,3	0	50,151	0	323,31
TUBA1A	Q71U36	Tubulin alpha-1A chain	33	74,3	0	50,135	0	28,191
TTYH3	Q9C0H2	Protein tweety homolog 3	3	5,9	5,9	57,544	0	10,213
TTR	A0A087WV45	Transthyretin	1	15,8	15,8	15,12	0	4,3455
TTN	Q8WZ42	Titin	5	0,2	0,2	3816	0,0038462	2,3227
TTC7B	H0YJS2	Tetratricopeptide repeat protein	1	3,7	3,7	29,278	0,0045732	2,229
TTC27	Q6P3X3	Tetratricopeptide repeat protein	2	2,4	2,4	96,631	0,0038344	2,2729
TST	Q16762	Thiosulfate sulfurtransferase	7	32	32	33,429	0	17,758
TSPAN9	B5MD23	Tetraspanin	3	10,7	10,7	29,559	0	29,33
TSPAN33	Q86UF1	Tetraspanin-33	5	16,6	16,6	31,538	0	56,986
TSPAN32	G3XAG6	Tetraspanin-32	3	22,6	22,6	28,44	0	12,284
TSPAN2	B1AKP2	Tetraspanin	1	6,4	6,4	20,564	0	2,8495
TSPAN15	H7C285	Tetraspanin-15	4	26,2	26,2	18,219	0	7,8638
TSPAN14	Q8NG11	Tetraspanin-14	5	20,7	20,7	30,69	0	10,276
TSN	Q15631	Translin	3	18,4	18,4	26,183	0	7,4706
TSG101	F5H442	Tumor susceptibility gene 101	4	13,4	13,4	40,917	0	5,5468
TRPC6	Q9Y210	Short transient receptor potential	8	9,5	9,5	106,33	0	11,671
TRIP10	W4VSQ9	Cdc42-interacting protein 4	3	6,6	6,6	67,73	0	4,0293
TRIO	F5H228	Triple functional domain protein	1	0,6	0,6	174,9	1	-2

TRIM58	Q8NG06	E3 ubiquitin-protein ligase	4	8	8	54,766	0	3,7478
TREML1	Q86YW5	Trem-like transcript 1 protein	7	41,2	41,2	32,678	0	50,188
TRAPPC3	A6NKE1	Trafficking protein particle	1	6,8	6,8	13,343	0,007278	1,762
TPT1	Q5W0H4	Translationally-controlled tumor	4	22,3	22,3	21,525	0	14,045
TPP2	Q5VZU9	Tripeptidyl-peptidase 2	14	13,5	13,5	139,76	0	22,392
TPP1	A0A2R8YGD1	Tripeptidyl-peptidase 1	6	23	23	50,889	0	44,363
TPM4	K7ENT6		31	75,7	13,8	28,492	0	11,767
TPM4	P67936	Tropomyosin alpha-4 chain	39	83,1	21,4	28,521	0	323,31
TPM3	A0A087WWU8		30	80,6	0	26,42	1	-2
TPM3	J3KN67		30	71,2	0	33,222	0	16,22
TPM2b	CON__Q3SX28	Tropomyosin beta chain	20	43,3	11,3	33,012	0	30,656
TPM1	H0YNC7		14	39,9	4,9	25,47	0	3,3254
TPM1	H7BYY1		22	58,1	19,8	28,747	0	65,416
TPM1	Q6ZN40	Tropomyosin alpha-1 chain	21	51,8	15	37,452	0	48,369
TPI1	P60174	Triosephosphate isomerase	19	78,7	78,7	30,791	0	234,21
TPD52L2	A0A087WZ51	Tumor protein D54	4	38,8	38,8	16,306	0	18,494
TOR4A	Q9NXH8	Torsin-4A	7	20,3	20,3	46,914	0	26,511
TOMM70A	O94826	Mitochondrial import receptor	2	4,6	4,6	67,454	0,0015898	2,6075
TOMM22	Q9NS69	Mitochondrial import receptor subunit TOM22 homolog	1	7,7	7,7	15,521	0,0067315	1,9862
TOM1L2	Q6ZVM7	TOM1-like protein 2	2	8,5	8,5	55,556	0	5,5766
TOLLIP	F2Z2Y8	Toll-interacting protein	2	12,7	12,7	23,28	0	5,6511
TNPO1	Q92973	Transportin-1	2	2,2	2,2	102,35	0	3,8321
TNIK	Q9UKE5	TRAF2 and NCK-interacting	9	9	9	154,94	0	19,61
TNFSF5	Q3L8U2	CD40 ligand	2	8,8	8,8	27,15	0	3,9573
TMX1	Q9H3N1	Thioredoxin-related	5	17,1	17,1	31,791	0	19,536
TMSB4X	P62328	Thymosin beta-4	9	88,6	88,6	5,0526	0	323,31
TMSB10	P63313	Thymosin beta-10	2	47,7	31,8	5,0256	0	4,9504
TMOD3	Q9NYL9	Tropomodulin-3	9	37,2	37,2	39,594	0	23,983
TMEM9B	Q9NQ34	Transmembrane protein 9B	1	14,6	14,6	22,531	0,0015835	2,5525
TMEM63A	O94886	CSC1-like protein 1	7	9	9	92,125	0	15,214
TMEM55A	Q8N4L2	Type 2 phosphatidylinositol 4,5-bisphosphate 4-phosphatase	3	10,9	10,9	28,081	0	5,3167

TMEM43	Q9BTV4	Transmembrane protein 43	1	2,2	2,2	44,875	0,0045283	2,1335
TMEM40	Q8WWA1	Transmembrane protein 40	2	10,7	10,7	25,495	0	6,3802
TMEM33	P57088	Transmembrane protein 33	2	8,9	8,9	27,978	0	3,5429
TMEM30A	Q9NV96	Cell cycle control protein 50A	7	21,6	21,6	40,683	0	22,307
TMEM189-UBE	I3L0A0	Ubiquitin-conjugating enzyme E2	4	10	10	42,208	0	6,5195
TMEM109	Q9BVC6	Transmembrane protein 109	2	5,3	5,3	26,21	0	5,7172
TMED9	Q9BVK6	Transmembrane emp24 domain-containing protein 9	3	15,7	15,7	27,277	0	5,7029
TMED8	Q6PL24	Protein TMED8	3	12,9	12,9	35,74	0	3,6913
TMED7-TICAM	A0A0A6YYA0	Transmembrane emp24 domain-containing protein 7	3	22,3	22,3	21,233	0	6,5812
TMED5	Q9Y3A6	Transmembrane emp24 domain-containing protein 5	1	5,2	5,2	26,005	0,0092527	1,5854
TMED2	F5GX39	Transmembrane emp24 domain-containing protein 2	2	19	19	13,631	0	18,661
TMED10	P49755	Transmembrane emp24 domain-containing protein 10	5	22,4	22,4	24,976	0	17,093
TMBIM1	C9JEN3	Protein lifeguard 3	2	14,2	14,2	11,535	0	12,447
TM9SF2	Q99805	Transmembrane 9 superfamily	8	13,6	13,6	75,775	0	22,321
TLN2	Q9Y4G6	Talin-2	28	7,4	1	271,61	0	7,2685
TLN1	Q9Y490	Talin-1	206	80,4	75,1	269,76	0	323,31
TLDC1	H3BUB0	TLD domain-containing protein 1	1	10,8	10,8	16,425	0	4,1214
TKT	P29401	Transketolase	14	29,1	29,1	67,877	0	50,806
TKFC	H0YCY6	Bifunctional ATP-dependent dihydroxyacetone kinase/FAD-	4	8,3	8,3	54,661	0	11,065
TJP2	A0A1B0GTW1	Tight junction protein ZO-2	22	21,9	21,9	140,73	0	79,454
TIMP3	P35625	Metalloproteinase inhibitor 3	4	20,9	20,9	24,145	0	20,264
TIMP1	Q5H9A7	Metalloproteinase inhibitor 1	5	45,5	45,5	16,057	0	11,989
THBS1	P07996	Thrombospondin-1	76	65,4	65,4	129,38	0	323,31
TGOLN2	O43493	Trans-Golgi network integral	2	5,4	5,4	51,018	0,0079365	1,6933
TGFB1I1	O43294	Transforming growth factor beta-1-induced transcript 1 protein	10	29,3	29,3	49,814	0	27,531
TGFB1	P01137	Transforming growth factor beta-	12	50	50	44,341	0	76,334

TFRC	G3V0E5	Transferrin receptor protein 1	6	10,5	10,5	75,963	0	6,6426
TFPI	P10646	Tissue factor pathway inhibitor	2	9,9	9,9	35,015	0	3,8077
TF	P02787	Serotransferrin	14	26,4	26,4	77,063	0	20,609
TESC	Q96BS2	Calcineurin B homologous protein	6	28,5	28,5	24,75	0	11,238
TEC	P42680	Tyrosine-protein kinase Tec	4	7,1	7,1	73,58	0	4,559
TCP1	P17987	T-complex protein 1 subunit	23	50,9	50,9	60,343	0	67,653
TCEB2	B8ZZU8	Transcription elongation factor B	3	36,3	36,3	12,527	0	8,8273
TCEB1	Q15369	Transcription elongation factor B	4	41,1	41,1	12,473	0	7,0291
TC2N	Q8N9U0	Tandem C2 domains nuclear	10	26,5	26,5	55,284	0	23,58
TBXAS1	Q53F23	Thromboxane-A synthase	20	41,4	41,4	60,649	0	87,056
TBXA2R	P21731	Thromboxane A2 receptor	4	10,2	10,2	37,431	0	5,6205
TBCB	K7EK42	Tubulin-folding cofactor B	2	9,4	9,4	21,296	0	2,8218
TBCA	E5RIW3	Tubulin-specific chaperone A	4	58,3	58,3	10,08	0	13,607
TBC1D32	A2A304	Protein broad-minded	1	8	8	24,855	1	-2
TBC1D13	Q9NVG8	TBC1 domain family member 13	2	4,8	4,8	46,553	0,00080386	2,7119
TARS	P26639	Threonine--tRNA ligase,	8	11,2	11,2	83,434	0	11,966
TAPBP	A0A0G2JKZ1	Tapasin	2	4,9	4,9	47,606	0,0073206	1,7939
TAOK3	Q9H2K8	Serine/threonine-protein kinase	7	8,7	7,2	105,4	0	8,6508
TAOK1	Q7L7X3	Serine/threonine-protein kinase	7	7,4	6,1	116,07	0	4,8249
TALDO1	P37837	Transaldolase	13	37,4	37,4	37,54	0	30,056
TAGLN3	Q9UI15	Transgelin-3	3	18,1	11,1	22,472	0	11,868
TAGLN2	P37802	Transgelin-2	19	81,4	74,4	22,391	0	323,31
SYTL4	Q96C24	Synaptotagmin-like protein 4	26	45,6	45,6	76,023	0	75,91
SYPL1	A0A0U1RQT9	Synaptophysin-like protein 1	1	8,1	8,1	14,967	0	6,7302
SYNCRIP	O60506	Heterogeneous nuclear	5	10	10	69,602	0	9,9595
SYK	P43405	Tyrosine-protein kinase SYK	17	36,7	36,7	72,065	0	48,86
SUSD1	H3BLV4	Sushi domain-containing protein	9	18,3	18,3	78,614	0	31,908
SURF4	Q5T8U5	Surfeit locus protein 4	2	11,8	11,8	21,127	0	3,7558
SUMO3	A8MUA9	Small ubiquitin-related modifier 4	1	8,9	8,9	15,317	0	3,568
SULT1A4	P0DMN0	Sulfotransferase 1A4	10	52,2	32,2	34,196	0	65,751
SULT1A1	P50225	Sulfotransferase 1A1	9	45,1	25,1	34,165	0	17,319
SUCLG1	P53597	Succinyl-CoA ligase [ADP/GDP-forming] subunit alpha,	1	4,6	4,6	36,249	0,0073801	1,8501

SUCLA2	A0A2R8YDQ9	Succinyl-CoA ligase [ADP-forming] subunit beta, mitochondrial	4	13,3	13,3	43,841	0	6,0562
STXBP5	Q5T5C0	Syntaxin-binding protein 5	9	11,6	11,6	127,57	0	11,256
STXBP3	O00186	Syntaxin-binding protein 3	10	18,9	18,9	67,764	0	24,197
STXBP2	Q15833	Syntaxin-binding protein 2	38	61,9	61,9	66,452	0	312,14
STX7	O15400	Syntaxin-7	8	32,2	32,2	29,815	0	17,551
STX4	Q12846	Syntaxin-4	6	25,9	25,9	34,18	0	11,311
STX16	B7ZBM4	Syntaxin-16	1	25	25	13,281	0	4,0806
STX12	B1AJQ6	Syntaxin-12	1	7	7	24,576	0	3,0231
STX11	O75558	Syntaxin-11	12	42,9	42,9	33,195	0	123,73
STT3B	Q8TCJ2	Dolichyl-diphosphooligosaccharide--	4	5,2	5,2	93,673	0	5,8895
STT3A	P46977	Dolichyl-diphosphooligosaccharide--	3	3,8	3,8	80,529	0	4,0105
STRAP	Q9Y3F4	Serine-threonine kinase receptor-associated protein	4	20,6	20,6	38,438	0	17,055
STON2	Q8WXE9	Stonin-2	4	3,9	3,9	101,16	0	4,9202
STOML2	Q9UJZ1	Stomatin-like protein 2,	2	9,3	9,3	38,534	0	5,5401
STOM	P27105	Erythrocyte band 7 integral	26	77,1	77,1	31,73	0	323,31
STK4	Q13043	Serine/threonine-protein kinase 4	4	9,4	7,6	55,63	0	8,6949
STK39	Q9UEW8	STE20/SPS1-related proline-alanine-rich protein kinase	1	2	2	59,473	0,0023603	2,5139
STK38	Q15208	Serine/threonine-protein kinase	3	8,8	8,8	54,19	0	5,6395
STK26	Q8NBY1	Serine/threonine-protein kinase	7	17,6	6,9	43,867	0	5,791
STK24	B4DR80	Serine/threonine-protein kinase	13	41	30,8	45,835	0	40,153
STIP1	P31948	Stress-induced-phosphoprotein 1	21	42,4	42,4	62,639	0	84,274
STIM1	G0XQ39	Stromal interaction molecule 1	11	18	18	88,645	0	24,933
STIL	H0Y702	SCL-interrupting locus protein	1	6,2	6,2	23,034	1	-2
STEAP3	Q658P3	Metalloreductase STEAP3	4	10,7	10,7	54,6	0	7,3591
STATH	P02808	Statherin	1	54,8	54,8	7,3044	0	8,4069
STAT5B	P51692	Signal transducer and activator of	8	11,7	11,7	89,865	0	16,921
STAT3	P40763	Signal transducer and activator of	11	22,3	22,3	88,067	0	25,012
STAT1	J3KPM9	Signal transducer and activator of	1	1,8	1,8	83,359	0,0099291	1,57

ST6GAL1	P15907	Beta-galactoside alpha-2,6-	3	9,9	9,9	46,604	0,001586	2,5906
ST3GAL6	C9IZL6	Type 2 lactosamine alpha-2,3-	2	7,3	7,3	25,538	0	3,0448
ST13	H7C3I1	Hsc70-interacting protein	7	50,7	50,7	16,219	0	20,786
SSR4	P51571	Translocon-associated protein	3	14,5	14,5	18,998	0	6,7941
SRI	C9J0K6	Sorcin	6	40	40	17,605	0	13,274
SRGN	P10124	Serglycin	5	32,9	32,9	17,652	0	13,762
SRC	P12931	Proto-oncogene tyrosine-protein	28	56,5	45,3	59,834	0	193,46
SQRDL	Q9Y6N5	Sulfide:quinone oxidoreductase,	6	15,3	15,3	49,96	0	9,3068
SPTBN1	A0A087WUZ3	Spectrin beta chain, non-	18	9,9	8,3	274,83	0	29,11
SPTB	H0YJE6		9	12,2	1,7	117,49	0	3,0894
SPTB	P11277	Spectrin beta chain, erythrocytic	31	19,7	12,9	246,47	0	75,046
SPTAN1	A0A0D9SF54	Spectrin alpha chain, non-	1	0,8	0,8	282,83	0,0073475	1,8193
SPN	C9JUK7	Leukosialin	2	5,1	5,1	35,962	0	5,5062
SPCS3	P61009	Signal peptidase complex subunit	3	17,8	17,8	20,313	0	5,6105
SPCS2	E9PL01	Signal peptidase complex subunit	3	15,9	15,9	17,027	0	2,8877
SPARC	P09486	SPARC	10	50,8	50,8	34,632	0	122,09
SOD2	P04179	Superoxide dismutase [Mn],	14	67,1	67,1	24,75	0	70,881
SOD1	P00441	Superoxide dismutase [Cu-Zn]	5	66,2	66,2	15,936	0	158,79
SNX3	O60493	Sorting nexin-3	3	14,8	14,8	18,762	0,0023474	2,4513
SNX1	H0YK42	Sorting nexin-2	2	6,9	6,9	35,592	0	3,3171
SNTB1	Q13884	Beta-1-syntrophin	6	13,2	13,2	58,06	0	12,649
SND1	Q7KZF4	Staphylococcal nuclease domain-containing protein 1	10	16,7	16,7	102	0	40,918
SNCA	P37840	Alpha-synuclein	8	77,9	77,9	14,46	0	117,56
SNAP91	E5RFU0	Phosphatidylinositol-binding clathrin assembly protein	1	10,3	10,3	12,865	0,0086331	1,6688
SNAP29	O95721	Synaptosomal-associated protein	2	12	12	28,97	0	7,7131
SNAP23	O00161	Synaptosomal-associated protein	13	64,9	64,9	23,354	0	157,73
SMS	P52788	Spermine synthase	2	5,2	5,2	41,268	0,0030984	2,3652
SMIM5	Q71RC9	Small integral membrane protein	1	13	13	8,5403	0	3,2769
SMIM1	H3BS66	Small integral membrane protein	3	91,9	91,9	4,1915	0	10,46
SLFN14	P0C7P3	Schlafen family member 14	3	3,8	3,8	103,91	0	4,2799
SLC9A9	Q8IVB4	Sodium/hydrogen exchanger 9	3	9,8	9,8	72,564	0	36,188

SLC9A3R1	O14745	Na(+)/H(+) exchange regulatory	9	33,8	33,8	38,868	0	34,95
SLC9A1	P19634	Sodium/hydrogen exchanger 1	4	6,4	6,4	90,762	0	6,0938
SLC6A6	A0A087WYN0	Transporter	2	5,7	5,7	52,025	0	4,6498
SLC6A4	P31645	Sodium-dependent serotonin	4	8,1	8,1	70,324	0	12,326
SLC4A1	A0A0A0MS98	Band 3 anion transport protein	4	11	11	60,763	0	7,5829
SLC44A2	Q8IWA5	Choline transporter-like protein 2	11	18,6	18,6	80,123	0	30,259
SLC44A1	Q8WWI5	Choline transporter-like protein 1	10	17,7	17,7	73,301	0	42,874
SLC43A3	Q8NBI5	Solute carrier family 43 member	3	6,3	6,3	54,528	0	12,946
SLC3A2	F5GZS6	4F2 cell-surface antigen heavy	5	10,7	10,7	64,872	0	7,7314
SLC2A3	P11169	Solute carrier family 2, facilitated glucose transporter member 3	12	18,8	18,8	53,924	0	167,17
SLC2A1	P11166	Solute carrier family 2, facilitated glucose transporter member 1	1	2	2	54,083	0,0052553	2,0755
SLC27A4	Q6P1M0	Long-chain fatty acid transport	1	1,4	1,4	72,063	0,0073421	1,816
SLC25A6	P12236	ADP/ATP translocase 3	10	31,5	8,1	32,866	0	3,6301
SLC25A5	P05141	ADP/ATP translocase 2	12	36,6	13,1	32,852	0	33,629
SLC25A3	Q00325	Phosphate carrier protein,	11	31,8	31,8	40,094	0	26,667
SLC25A15	Q9Y619	Mitochondrial ornithine	1	5,6	5,6	32,736	0	2,7532
SLC25A11	I3L1P8	Mitochondrial 2-oxoglutarate/malate carrier	5	19,9	19,9	32,182	0	14,372
SLC25A1	P53007	Tricarboxylate transport protein,	2	7,4	7,4	34,012	0,0067164	1,9405
SLC16A7	O60669	Monocarboxylate transporter 2	3	5,2	5,2	52,2	0,009915	1,5607
SLC16A3	O15427	Monocarboxylate transporter 4	4	10,1	10,1	49,469	0	12,12
SLA2	Q9H6Q3	Src-like-adapter 2	4	23	23	28,585	0	18,602
SKAP2	O75563	Src kinase-associated	14	45,1	45,1	41,216	0	56,264
SIDT1	Q9NXL6	SID1 transmembrane family	1	1,2	1,2	93,838	0,009901	1,5547
SHMT2	P34897	Serine hydroxymethyltransferase,	6	17,1	17,1	55,992	0	8,1129
SH3GLB1	A0A087WW40	Endophilin-B1	2	7,1	7,1	44,26	0,0031153	2,4208
SH3BGRL3	Q5T123	SH3 domain-binding glutamic acid	5	51,1	51,1	9,3804	0	88,911
SH3BGRL2	Q9UJC5	SH3 domain-binding glutamic acid	3	36,4	36,4	12,326	0	5,9978
SH3BGRL	O75368	SH3 domain-binding glutamic acid	6	61,4	61,4	12,774	0	51,436
SFXN3	A0A0A0MS41	Sideroflexin	4	17,1	17,1	35,503	0	9,272
SFN	P31947	14-3-3 protein sigma	4	13,3	3,2	27,774	0,007391	1,8533

SETDB1	E9PAP1	Histone-lysine N-	1	10,8	10,8	12,422	0,0073964	1,8589
SERPINH1	P50454	Serpin H1	6	20,1	20,1	46,44	0	15,267
SERPINE1	P05121	Plasminogen activator inhibitor 1	6	16,9	16,9	45,059	0	17,477
SERPINB9	P50453	Serpin B9	9	29,5	23,7	42,403	0	22,185
SERPINB6	A0A024QZX5	Serpin B6	15	52,4	50,3	43,024	0	70,449
SERPINB1	P30740	Leukocyte elastase inhibitor	21	60,9	58,3	42,741	0	107,42
SERPINA1	A0A0G2JRN3	Alpha-1-antitrypsin	7	23,7	23,7	40,234	0	14,28
SERINC3	Q13530	Serine incorporator 3	2	4,9	4,9	52,58	0	3,3171
SELP	Q5R349	P-selectin	30	41,2	41,2	86,472	0	268,58
SEC62	F8WF48	Translocation protein SEC62	1	15,3	15,3	9,8703	0	3,3772
SEC23A	F5H365	Protein transport protein Sec23A	2	4,1	4,1	82,968	0	4,0945
SEC22B	O75396	Vesicle-trafficking protein SEC22b	4	20,5	20,5	24,593	0	14,317
SEC11A	H0YNG3	Signal peptidase complex	3	17,8	17,8	18,651	0	7,5415
SDPR	O95810	Serum deprivation-response	22	51,1	51,1	47,173	0	181,43
SDHB	P21912	Succinate dehydrogenase [ubiquinone] iron-sulfur subunit,	2	8,2	8,2	31,629	0	3,9686
SDHA	P31040	Succinate dehydrogenase [ubiquinone] flavoprotein	7	13	13	72,691	0	16,443
SCYL2	F8VSC5	SCY1-like protein 2	3	6	6	77,053	0,0030912	2,3486
SCPEP1	Q9HB40	Retinoid-inducible serine	2	4,4	4,4	50,83	0	4,1879
SCFD1	Q8WVM8	Sec1 family domain-containing	2	4,5	4,5	72,379	0,00080192	2,6726
SCCPDH	Q8NBX0	Saccharopine dehydrogenase-like	2	6,8	6,8	47,151	0	3,5514
SCAMP2	O15127	Secretory carrier-associated	2	9,7	9,7	36,648	0	30,076
SARS	Q5T5C7	Serine--tRNA ligase, cytoplasmic	5	10,8	10,8	61,312	0	8,2132
SAR1A	Q9NR31	GTP-binding protein SAR1a	11	63,6	63,6	22,367	0	37,973
SACM1L	Q9NTJ5	Phosphatidylinositide	19	34,1	34,1	66,966	0	43,518
S100A7	P31151	Protein S100-A7	2	22,8	22,8	11,471	0,0099432	1,574
S100A6	R4GN98	Protein S100	5	56,5	56,5	9,681	0	12,46
S100A4	P26447	Protein S100-A4	6	55,4	55,4	11,728	0	17
RUVBL1	E7ETR0	RuvB-like 1	3	11,1	11,1	34,782	0	5,1279
RTN4	Q9NQC3	Reticulon-4	8	11	11	129,93	0	69,099
RTN3	O95197	Reticulon-3	1	1,1	1,1	112,61	0,0023659	2,5254
RTN2	K7EMR7	Reticulon	3	9,2	9,2	29,519	0	3,0668

RTN1	A8MT72	Reticulon	2	8,8	8,8	21,862	0,0073638	1,84
RTCB	Q9Y3I0	tRNA-splicing ligase RtcB	2	5,9	5,9	55,21	0	3,989
RSU1	Q15404	Ras suppressor protein 1	18	81,9	81,9	31,54	0	213,08
RRAS	P10301	Ras-related protein R-Ras	7	44,5	44,5	23,48	0	35,693
RPSA	C9J9K3	40S ribosomal protein SA	3	14,8	14,8	29,404	0	3,6349
RPS6KA1	E9PMM7	Ribosomal protein S6 kinase	1	14,3	14,3	13,098	0,0008	2,6567
RPN2	P04844	Dolichyl-diphosphooligosaccharide--	8	21,6	21,6	69,283	0	74,362
RPN1	P04843	Dolichyl-diphosphooligosaccharide--	22	41,7	41,7	68,569	0	68,318
RPLP2	P05387	60S acidic ribosomal protein P2	2	27	27	11,665	0	5,4347
RPIA	P49247	Ribose-5-phosphate isomerase	1	5,1	5,1	33,269	0,0092857	1,6189
RP2	O75695	Protein XRP2	5	11,7	11,7	39,641	0	9,0309
ROCK2	O75116	Rho-associated protein kinase 2	37	32,6	27,5	160,9	0	118,41
ROCK1	A0A0U1RQV4	Rho-associated protein kinase 1	10	9,4	3,3	133,07	0	3,0265
RNH1	P13489	Ribonuclease inhibitor	11	28	28	49,973	0	34,479
RNF11	Q9Y3C5	RING finger protein 11	1	5,8	5,8	17,444	0,005279	2,1111
RIT1	Q92963	GTP-binding protein Rit1	1	5,5	5,5	25,145	0,0093123	1,649
RHOG	P84095	Rho-related GTP-binding protein	6	40,8	35,1	21,308	0	24,103
RHOF	Q9HBH0	Rho-related GTP-binding protein	8	47,4	47,4	23,625	0	20,438
RHOC	P08134	Rho-related GTP-binding protein	10	51,8	17,6	22,006	0	6,6517
RHOA	P61586	Transforming protein RhoA	12	59,6	25,4	21,768	0	92,75
RHEB	Q15382	GTP-binding protein Rheb	7	28,8	28,8	20,497	0	14,426
RGS6	A0A0A0MRA9	Regulator of G-protein signaling 6	2	5	5	48,509	0	4,1475
RGS19	P49795	Regulator of G-protein signaling	2	9,7	9,7	24,635	0	4,0364
RGS18	Q9NS28	Regulator of G-protein signaling	7	39,1	39,1	27,582	0	11,327
RGS10	O43665	Regulator of G-protein signaling	6	45,7	45,7	20,236	0	23,919
RER1	O15258	Protein RER1	2	13,8	13,8	22,958	0	5,0389
RENBP	P51606	N-acylglucosamine 2-epimerase	4	9,6	9,6	48,83	0	6,9745
REEP5	E2QRG8	Receptor expression-enhancing	2	10,9	10,9	18,121	0	4,6373
RDX	A0A2R8Y7M3	Radixin	16	22,5	3,7	62,93	1	-2
RDH11	Q8TC12	Retinol dehydrogenase 11	8	34,6	34,6	35,386	0	42,418
RASGRP2	Q7LDG7	RAS guanyl-releasing protein 2	19	41,9	41,9	69,248	0	183,8

RASA3	Q14644	Ras GTPase-activating protein 3	36	56,6	56,6	95,698	0	177,54
RASA1	E9PGC0	Ras GTPase-activating protein 1	7	10,6	10,6	101,79	0	13,95
RARS	P54136	Arginine--tRNA ligase,	3	5,8	5,8	75,378	0	5,4404
RARRES2	C9J8S2	Retinoic acid receptor responder	1	7,5	7,5	17,813	0,0031226	2,4365
RAP2B	P61225	Ras-related protein Rap-2b	8	54,6	28,4	20,504	0	87,058
RAP2A	P10114	Ras-related protein Rap-2a	6	37,7	11,5	20,615	0,0086207	1,664
RAP1GDS1	P52306	Rap1 GTPase-GDP dissociation	8	15,2	15,2	66,316	0	14,471
RAP1GAP2	A0A1B0GV05	Rap1 GTPase-activating protein 2	7	12,6	12,6	85,181	0	13,371
RAP1B	P61224	Ras-related protein Rap-1b	18	80,4	29,9	20,825	0	249,91
RAP1A	P62834	Ras-related protein Rap-1A	15	59,2	8,2	20,987	0	10,674
RANBP1	C9JJ34	Ran-specific GTPase-activating	3	27,6	27,6	18,762	0	14,907
RAN	B5MDF5	GTP-binding nuclear protein Ran	7	36,5	36,5	26,224	0	14,837
RALB	P11234	Ras-related protein Ral-B	9	44,7	23,3	23,408	0	47,332
RALA	P11233	Ras-related protein Ral-A	6	33	11,7	23,567	0	4,8903
RAD23B	P54727	UV excision repair protein RAD23	2	4,6	4,6	43,171	0,003108	2,4031
RAC2	P15153	Ras-related C3 botulinum toxin	9	49,5	19,3	21,429	0	16,29
RAC1	P63000	Ras-related C3 botulinum toxin	11	50,5	20,3	21,45	0	43,892
RAB8B	Q92930	Ras-related protein Rab-8B	7	39,1	19,3	23,584	0	25,564
RAB8A	P61006	Ras-related protein Rab-8A	10	44	27,5	23,668	0	62,163
RAB7A	P51149	Ras-related protein Rab-7a	14	77,3	77,3	23,489	0	91,46
RAB6C	Q9H0N0	Ras-related protein Rab-6C	4	16,9	0	28,355	0	6,5726
RAB6B	Q9NRW1	Ras-related protein Rab-6B	12	55,8	22,1	23,461	0	44,38
RAB6A	P20340	Ras-related protein Rab-6A	10	49,5	16,8	23,593	0	11,418
RAB5C	P51148	Ras-related protein Rab-5C	8	52,8	38	23,482	0	49,702
RAB5B	P61020	Ras-related protein Rab-5B	5	27	12,1	23,707	0	5,4445
RAB5A	P20339	Ras-related protein Rab-5A	5	27	12,1	23,658	0	8,9308
RAB4A	P20338	Ras-related protein Rab-4A	4	22,9	16,5	24,389	0	16,459
RAB3GAP2	Q9H2M9	Rab3 GTPase-activating protein non-catalytic subunit	2	1,6	1,6	155,98	0,005997	2,0285
RAB37	B7Z3L0	Ras-related protein Rab-37	9	46,4	46,4	22,149	0	11,564
RAB35	Q15286	Ras-related protein Rab-35	3	17,9	17,9	23,025	0	3,9165
RAB32	Q13637	Ras-related protein Rab-32	10	60,4	55,6	24,997	0	38,542
RAB31	J3QR51	Ras-related protein Rab-31	3	58,1	58,1	8,0392	0	4,0574

RAB2A	P61019	Ras-related protein Rab-2A	9	43,4	43,4	23,545	0	42,829
RAB27B	O00194	Ras-related protein Rab-27B	17	78,9	78,9	24,608	0	202,51
RAB21	Q9UL25	Ras-related protein Rab-21	5	27,1	27,1	24,347	0	32,044
RAB1B	Q9H0U4	Ras-related protein Rab-1B	15	71,1	44,8	22,171	0	107,33
RAB1A	P62820	Ras-related protein Rab-1A	14	72,7	39	22,677	0	26,326
RAB18	Q5W0J0	Ras-related protein Rab-18	4	31,7	31,7	18,023	0	9,8263
RAB14	P61106	Ras-related protein Rab-14	15	74,9	74,9	23,897	0	80,572
RAB13	P51153	Ras-related protein Rab-13	7	36	23,2	22,774	0	12,676
RAB11FIP3	X6RFI8	Rab11 family-interacting protein	1	1,6	1,6	75,915	0,0045767	2,2308
RAB11B	Q15907	Ras-related protein Rab-11B	16	67,4	67,4	24,488	0	192,9
RAB10	P61026	Ras-related protein Rab-10	16	69,5	60,5	22,541	0	141,87
QARS	H7C0R3	Glutamine--tRNA ligase	3	15	15	28,565	0	4,9433
PYGL	P06737	Glycogen phosphorylase, liver	19	28,5	21,8	97,147	0	33,901
PYGB	P11216	Glycogen phosphorylase, brain	24	33,8	27,4	96,695	0	63,754
PVRL2	Q92692	Nectin-2	7	15,8	15,8	57,741	0	20,327
PTTG1IP	P53801	Pituitary tumor-transforming gene 1 protein-interacting	5	21,7	21,7	20,324	0	14,489
PTRHD1	Q6GMV3	Putative peptidyl-tRNA hydrolase	2	39,3	39,3	15,805	0	11,714
PTPRJ	Q12913	Receptor-type tyrosine-protein	35	29	29	145,94	0	275,26
PTPRC	P08575	Receptor-type tyrosine-protein	12	11,4	11,4	147,48	0	15,291
PTPRB	F8VU56	Protein-tyrosine-phosphatase	1	0,4	0,4	239,56	0,003084	2,336
PTPRA	P18433	Receptor-type tyrosine-protein	9	14,8	14,8	90,718	0	23,878
PTPN6	P29350	Tyrosine-protein phosphatase	21	44,2	44,2	67,56	0	85,983
PTPN12	Q05209	Tyrosine-protein phosphatase	6	11	11	88,105	0	23,888
PTPN11	Q06124	Tyrosine-protein phosphatase	8	19,1	19,1	68,436	0	34,989
PTP4A2	E9PMY3	Protein tyrosine phosphatase	4	79,4	79,4	7,2082	0	18,462
PTK2	H0YBP1	Focal adhesion kinase 1	3	3,1	3,1	115,45	0	3,8998
PTGS1	P23219	Prostaglandin G/H synthase 1	19	40,4	40,4	68,686	0	110,77
PTGIR	M0QZW0	Prostacyclin receptor	3	10,9	10,9	18,774	0	5,6566
PTGES3	Q15185	Prostaglandin E synthase 3	5	31,2	31,2	18,697	0	14,709
PTCD2	E5RHU2		1	11,8	11,8	10,173	0	2,8943
PTBP1	A6NLN1	Polypyrimidine tract-binding	3	6,8	6,8	56,51	0	4,0974

PSTPIP2	Q9H939	Proline-serine-threonine phosphatase-interacting protein	11	26,9	26,9	38,858	0	19,861
PSME1	Q06323	Proteasome activator complex	9	36,5	36,5	28,723	0	11,034
PSMD7	P51665	26S proteasome non-ATPase	2	9	9	37,025	0	7,5892
PSMD6	Q15008	26S proteasome non-ATPase	3	10,5	10,5	45,531	0	7,4022
PSMD5	Q16401	26S proteasome non-ATPase	3	7,1	7,1	56,195	0	5,6178
PSMD3	O43242	26S proteasome non-ATPase	2	4,9	4,9	60,977	0	3,764
PSMD2	Q13200	26S proteasome non-ATPase	4	5,8	5,8	100,2	0	9,0954
PSMD14	O00487	26S proteasome non-ATPase	2	12,6	12,6	34,577	0	4,5164
PSMD13	J3KNQ3	26S proteasome non-ATPase	2	9,7	9,7	39,829	0	2,9175
PSMD12	O00232	26S proteasome non-ATPase	2	5	5	52,904	0	4,8905
PSMD11	O00231	26S proteasome non-ATPase	4	10,2	10,2	47,463	0	7,7317
PSMC6	A0A087X2I1	26S protease regulatory subunit	3	8,4	8,4	45,796	0,0052711	2,1055
PSMC3	E9PM69	26S protease regulatory subunit	4	15,9	15,9	44,323	0	9,2437
PSMC2	C9JLS9	26S protease regulatory subunit 7	1	8,5	8,5	14,636	0	2,7487
PSMC1	P62191	26S protease regulatory subunit 4	2	5,7	5,7	49,184	0	3,6699
PSMB9	A2ACR1	Proteasome subunit beta type	2	11,2	11,2	20,96	0	5,4501
PSMB6	P28072	Proteasome subunit beta type-6	1	4,6	4,6	25,357	0,0086393	1,6708
PSMB4	P28070	Proteasome subunit beta type-4	1	3	3	29,204	0,0038285	2,2624
PSMB3	A0A087WUL2	Proteasome subunit beta type-3	3	28,3	28,3	16,161	0	8,4575
PSMB2	P49721	Proteasome subunit beta type-2	4	26,4	26,4	22,836	0	7,3796
PSMB10	J3QL48	Proteasome subunit beta type-10	1	26	26	8,2133	0	3,2571
PSMB1	P20618	Proteasome subunit beta type-1	2	10	10	26,489	0	4,3772
PSMA7	O14818	Proteasome subunit alpha type-7	8	42,3	42,3	27,887	0	22,517
PSMA6	G3V5Z7	Proteasome subunit alpha type	6	29,4	29,4	28,147	0	23,332
PSMA5	P28066	Proteasome subunit alpha type-5	7	29,9	29,9	26,411	0	17
PSMA4	H0YMZ1	Proteasome subunit alpha type	6	34,1	34,1	24,526	0	10,147
PSMA3	P25788	Proteasome subunit alpha type-3	5	25,9	25,9	28,433	0	15,641
PSMA2	A0A024RA52	Proteasome subunit alpha type	4	25,6	25,6	25,898	0	8,7068
PSMA1	P25786	Proteasome subunit alpha type-1	10	43,3	43,3	29,555	0	20,552
PSM8	X5D2R7	Proteasome subunit beta type	4	18,5	18,5	30,354	0	8,4859
PRUNE	Q86TP1	Protein prune homolog	3	9,3	9,3	50,199	0	21,465

PRTFDC1	Q9NRG1	Phosphoribosyltransferase domain-containing protein 1	2	8,9	5,8	25,673	0,0015962	2,6194
PRSS3	B1AN99	Trypsin-3	2	13,6	13,6	19,288	0	7,2205
PRSS1	H0Y8D1	Putative trypsin-6	1	14,1	14,1	15,421	0	3,7787
PRR4	A0A0J9YXF8	Proline-rich protein 4	1	10,2	10,2	16,89	0	20,775
PRPS1	B1ALA9	Ribose-phosphate	2	10,2	10,2	31,392	0	9,5917
PROS1	P07225	Vitamin K-dependent protein S	16	32,8	32,8	75,122	0	60,565
PRNP	P04156	Major prion protein	2	9,5	9,5	27,661	0	6,9198
PRKG1	B1ALS0		7	35,3	14,7	35,486	0	8,8938
PRKCSH	K7ELL7	Glucosidase 2 subunit beta	9	20,6	20,6	60,192	0	21,618
PRKCQ	A0A087X0I9	Protein kinase C	6	8,7	8,7	77,969	0	9,4969
PRKCD	Q05655	Protein kinase C delta type	15	25,9	25,9	77,504	0	38,822
PRKCB	P05771	Protein kinase C beta type	19	41,7	30,8	76,868	0	62,364
PRKCA	P17252	Protein kinase C alpha type	10	21,9	11	76,749	0	12,291
PRKAR2B	P31323	cAMP-dependent protein kinase type II-beta regulatory subunit	9	29,4	29,4	46,302	0	27,028
PRKAR1B	H7BYW5	cAMP-dependent protein kinase type I-beta regulatory subunit	6	21,1	7	26,937	0	7,0686
PRKAR1A	P10644	cAMP-dependent protein kinase type I-alpha regulatory subunit	24	63,3	54,3	42,981	0	125,19
PRKACB	A0A0A0MS54	cAMP-dependent protein kinase catalytic subunit beta	17	39,5	15,1	41,308	0	46,321
PRKACA	P17612	cAMP-dependent protein kinase catalytic subunit alpha	14	32,8	8	40,589	0	6,915
PRKAA1	Q13131	5-AMP-activated protein kinase catalytic subunit alpha-1	2	4,5	4,5	64,009	0	6,0466
PREP	P48147	Prolyl endopeptidase	2	4,5	4,5	80,699	0	7,5884
PRDX6	P30041	Peroxiredoxin-6	24	83,9	83,9	25,035	0	217,1
PRDX5	P30044	Peroxiredoxin-5, mitochondrial	9	45,8	45,8	22,086	0	39,112
PRDX4	Q13162	Peroxiredoxin-4	7	25,1	18,1	30,54	0	9,4391
PRDX3	P30048	Thioredoxin-dependent peroxide reductase, mitochondrial	9	43,8	43,8	27,692	0	59,816
PRDX2	P32119	Peroxiredoxin-2	3	17,7	17,7	21,892	0	5,491

PRDX1	Q06830	Peroxiredoxin-1	13	61,3	51,8	22,11	0	48,914
PPP6C	O00743	Serine/threonine-protein phosphatase 6 catalytic subunit	3	11,5	11,5	35,144	0	6,6526
PPP3R1	F6U1T9	Calcineurin subunit B type 1	1	7,5	7,5	18,208	0,0023511	2,4741
PPP3CA	Q08209	Serine/threonine-protein phosphatase 2B catalytic subunit	6	13,2	13,2	58,687	0	9,6421
PPP2R4	A6PVN5	Serine/threonine-protein	5	17,3	17,3	37,375	0	12,782
PPP2R2A	P63151	Serine/threonine-protein phosphatase 2A 55 kDa	1	2,5	2,5	51,691	0,0066964	1,9106
PPP2R1A	P30153	Serine/threonine-protein phosphatase 2A 65 kDa	15	34	34	65,308	0	55,114
PPP2CA	P67775	Serine/threonine-protein phosphatase 2A catalytic subunit	4	19,7	19,7	35,594	0	8,7715
PPP1R7	H7C003	Protein phosphatase 1 regulatory	3	9	9	38,633	0	9,0735
PPP1R14A	K7EJB8	Protein phosphatase 1 regulatory	1	9,1	9,1	13,886	0	2,908
PPP1R12A	F8VZN8	Protein phosphatase 1 regulatory	3	5,9	5,9	76,532	0	22,101
PPP1CC	F8VYE8	Serine/threonine-protein	11	45,7	7,9	34,95	0	10,258
PPP1CB	P62140	Serine/threonine-protein phosphatase PP1-beta catalytic	11	42,2	11,3	37,186	0	6,0999
PPP1CA	P62136	Serine/threonine-protein phosphatase PP1-alpha catalytic	14	50,9	16,1	37,512	0	99,915
PPM1F	B5MCT7	Protein phosphatase 1F	6	35,3	35,3	31,263	0	13,152
PPM1A	P35813	Protein phosphatase 1A	11	38,2	38,2	42,447	0	19,195
PPIF	P30405	Peptidyl-prolyl cis-trans	10	64,7	64,7	22,04	0	107,91
PPIB	P23284	Peptidyl-prolyl cis-trans	13	56,9	56,9	23,742	0	72,713
PPIA	P62937	Peptidyl-prolyl cis-trans	14	78,8	78,8	18,012	0	187,63
PPBP	P02775	Platelet basic protein	9	53,9	53,9	13,894	0	228,52
PPA2	Q9H2U2	Inorganic pyrophosphatase 2,	3	11,1	11,1	37,92	0	2,8356
PNP	P00491	Purine nucleoside phosphorylase	18	77,5	77,5	32,118	0	179,61
PNKD	Q8N490	Probable hydrolase PNKD	3	10,4	10,4	42,875	0	12,099
PMVK	Q15126	Phosphomevalonate kinase	2	14,1	14,1	21,995	0,0031104	2,4056
PLXNB3	Q9ULL4	Plexin-B3	5	2,9	2,9	206,84	0	5,0093
PLXNB2	O15031	Plexin-B2	11	8,2	8,2	205,12	0	14,439

PLXNA4	Q9HCM2	Plexin-A4	8	5,6	5,6	212,45	0	22,46
PLXDC2	Q6UX71	Plexin domain-containing protein	2	5,5	5,5	59,582	0,0072464	1,7243
PLS3	P13797	Plastin-3	16	34,4	34,4	70,81	0	50,133
PLP2	Q04941	Proteolipid protein 2	1	8,6	8,6	16,691	0	6,2233
PLIN3	K7ERZ3	Perilipin-3	1	6,2	6,2	31,872	0	3,1538
PLG	P00747	Plasminogen	6	8,6	8,6	90,568	0	12,605
PLEKHO2	Q8TD55	Pleckstrin homology domain-containing family O member 2	6	18	18	53,349	0	22,778
PLEKHF2	Q9H8W4	Pleckstrin homology domain-containing family F member 2	1	4,8	4,8	27,797	0,0072516	1,7308
PLEK	P08567	Pleckstrin	27	64,6	64,6	40,124	0	323,31
PLEC	Q15149	Plectin	12	3	3	531,78	0	11,75
PLCG2	A0A0A0MRF9	Phosphoinositide phospholipase	5	5	5	146,06	0	6,7706
PLCB4	Q15147	1-phosphatidylinositol 4,5-bisphosphate phosphodiesterase	1	1	1	134,46	0,001581	2,533
PLCB2	Q00722	1-phosphatidylinositol 4,5-bisphosphate phosphodiesterase	5	6	6	134,02	0	9,4275
PLA2G4A	P47712	Cytosolic phospholipase A2	3	6,1	6,1	85,238	0	4,7252
PKM	H3BTN5	Pyruvate kinase	39	72,8	0	53,045	0	2,7801
PKM	P14618	Pyruvate kinase PKM	46	82,9	9,4	57,936	0	323,31
PKHD1L1	Q86WI1	Fibrocystin-L	29	9,3	9,3	465,73	0	83,391
PITPNB	B3KYB6	Phosphatidylinositol transfer	2	28,3	28,3	13,781	0	3,722
PIP4K2A	P48426	Phosphatidylinositol 5-phosphate 4-kinase type-2 alpha	17	39,2	32,5	46,224	0	118,8
PI4KA	P42356	Phosphatidylinositol 4-kinase	6	3,2	3,2	236,83	0	6,3165
PHB2	J3KPX7	Prohibitin-2	10	42,6	42,6	33,239	0	53,715
PHB	P35232	Prohibitin	11	43,4	43,4	29,804	0	30,843
PGRMC2	O15173	Membrane-associated progesterone receptor	4	22,9	18,8	23,818	0	8,1613
PGRMC1	O00264	Membrane-associated progesterone receptor	6	24,6	20	21,671	0	13,695
PGM2	Q96G03	Phosphoglucomutase-2	9	21,4	21,4	68,283	0	24,275
PGM1	P36871	Phosphoglucomutase-1	20	48,2	48,2	61,448	0	49,041

PGLS	O95336	6-phosphogluconolactonase	6	34,1	34,1	27,547	0	12,422
PGK1	P00558	Phosphoglycerate kinase 1	29	74,3	74,3	44,614	0	279,82
PGD	P52209	6-phosphogluconate dehydrogenase, decarboxylating	18	46,4	46,4	53,139	0	78,168
PGAM1	P18669	Phosphoglycerate mutase 1	11	56,7	56,7	28,804	0	51,452
PFN1	P07737	Profilin-1	16	95,7	95,7	15,054	0	171,35
PFKP	Q01813	ATP-dependent 6-phosphofructokinase, platelet	25	36,2	32,7	85,595	0	185,49
PFKL	P17858	ATP-dependent 6-	17	27,8	24,2	85,018	0	31,748
PFDN2	Q9UHV9	Prefoldin subunit 2	1	7,8	7,8	16,648	0	2,9807
PF4V1	P10720	Platelet factor 4 variant	8	67,3	33,7	11,553	0	51,541
PF4	P02776	Platelet factor 4	8	47,5	12,9	10,845	0	63,802
PEF1	Q9UBV8	Peflin	2	8,5	8,5	30,381	0	5,0076
PECAM1	P16284	Platelet endothelial cell adhesion	37	55	55	82,521	0	323,31
PEBP1	P30086	Phosphatidylethanolamine-	7	58,8	58,8	21,057	0	63,916
PEAR1	Q5VY43	Platelet endothelial aggregation	4	5	5	110,67	0	14,718
PDLIM7	Q9NR12	PDZ and LIM domain protein 7	9	27,1	27,1	49,844	0	32,363
PDLIM5	Q96HC4	PDZ and LIM domain protein 5	9	18,8	18,8	63,944	0	31,981
PDLIM1	O00151	PDZ and LIM domain protein 1	21	83,3	83,3	36,071	0	274,48
PDIA6	Q15084	Protein disulfide-isomerase A6	15	44,8	44,8	48,121	0	88,012
PDIA5	Q14554	Protein disulfide-isomerase A5	8	19,5	19,5	59,594	0	20,043
PDIA4	P13667	Protein disulfide-isomerase A4	13	24,5	24,5	72,932	0	19,631
PDIA3	H7BZJ3		7	58,5	11,4	13,519	0	14,282
PDIA3	P30101	Protein disulfide-isomerase A3	31	63,2	51,7	56,782	0	219,36
PDHB	P11177	Pyruvate dehydrogenase E1 component subunit beta,	5	18,7	18,7	39,233	0	14,322
PDE5A	O76074	cGMP-specific 3,5-cyclic	26	36	36	99,984	0	108,15
PDE3A	Q14432	cGMP-inhibited 3,5-cyclic	2	2,4	2,4	124,98	0	3,4697
PDCD6IP	Q8WUM4	Programmed cell death 6-	22	31,2	31,2	96,022	0	48,077
PDCD6	O75340	Programmed cell death protein 6	6	31,9	31,9	21,868	0	17,282
PDCD10	Q9BUL8	Programmed cell death protein	10	52,4	52,4	24,701	0	53,445
PCYOX1L	Q8NBM8	Prenylcysteine oxidase-like	5	14,8	14,8	54,646	0	19,315
PCMT1	A0A0A0MRJ6	Protein-L-isoaspartate O-	9	48,8	48,8	30,315	0	50,114

PCBP2	Q15366	Poly(rC)-binding protein 2	6	22,5	11,8	38,58	0	7,4257
PCBP1	Q15365	Poly(rC)-binding protein 1	7	26,1	15,2	37,497	0	28,995
PARVB	Q9HBI1	Beta-parvin	23	59,1	59,1	41,714	0	323,31
PARK7	Q99497	Protein deglycase DJ-1	12	72	72	19,891	0	92,769
PANX1	Q96RD7	Pannexin-1	5	19,5	19,5	48,05	0	10,96
PAK2	Q13177	Serine/threonine-protein kinase	7	13,2	13,2	58,042	0	7,5911
PAICS	P22234	Multifunctional protein ADE2	9	26,4	26,4	47,079	0	20,511
PAFAH1B2	P68402	Platelet-activating factor acetylhydrolase IB subunit beta	3	28,8	28,8	25,569	0	18,838
PAFAH1B1	I3L3N5	Platelet-activating factor acetylhydrolase IB subunit alpha	3	17,3	17,3	23,691	0	3,5297
PACSIN2	A0A0U1RR22	Protein kinase C and casein kinase substrate in neurons	9	21,7	21,7	51,828	0	19,639
PACSIN1	F6U236		1	2,7	2,7	46,146	0,0072939	1,7711
PACS1	Q6VY07	Phosphofurin acidic cluster	3	3,8	3,8	104,9	0	5,7739
PABPC1	P11940	Polyadenylate-binding protein 1	6	11	11	70,67	0	8,1641
PA2G4	Q9UQ80	Proliferation-associated protein	12	33,5	33,5	43,786	0	20,548
P4HB	P07237	Protein disulfide-isomerase	25	46,7	46,7	57,116	0	107,59
P2RX1	P51575	P2X purinoceptor 1	7	23,3	23,3	44,98	0	45,7
OXSR1	C9JIG9	Serine/threonine-protein kinase	1	1,7	1,7	51,856	0,0092659	1,5906
OXCT1	P55809	Succinyl-CoA:3-ketoacid coenzyme A transferase 1,	3	6,5	6,5	56,157	0	5,4093
OTUB1	F5GYN4	Ubiquitin thioesterase OTUB1	5	25,3	25,3	28,05	0	11,741
OSTF1	Q92882	Osteoclast-stimulating factor 1	1	11,7	11,7	23,787	0	6,808
OSTC	A0A087WUD3	Oligosaccharyltransferase	1	14,5	14,5	9,369	0	3,1191
ORM2	P19652	Alpha-1-acid glycoprotein 2	2	9,5	9,5	23,602	0	3,254
OPTC	Q9UBM4	Opticin	1	4,8	4,8	37,26	0,0073368	1,8077
OPA1	A0A2R8Y4G4	Dynamin-like 120 kDa protein,	2	6,6	6,6	42,638	0	2,9353
OLA1	J3KQ32	Obg-like ATPase 1	8	24,8	24,8	46,937	0	15,812
OGDH	E9PFG7	2-oxoglutarate dehydrogenase,	3	4,2	4,2	99,015	0,0015924	2,6097
OCIAD1	D6R918	OCIA domain-containing protein	1	24,7	24,7	8,2873	0,0073746	1,8495
NUTF2	P61970	Nuclear transport factor 2	5	56,7	56,7	14,478	0	23,155
NT5C3A	X6RM59	5-nucleotidase	2	8,5	8,5	37,405	0	3,0703

NSF	I3L0N3	Vesicle-fusing ATPase	9	12,6	12,6	82,091	0	9,2838
NRGN	Q92686	Neurogranin	2	19,2	19,2	7,6184	0	3,9169
NRBP1	Q9UHY1	Nuclear receptor-binding protein	3	7,3	7,3	59,844	0	6,3528
NRAS	P01111	GTPase NRas	4	23,8	4,8	21,229	0	6,217
NPLOC4	Q8TAT6	Nuclear protein localization	1	1,5	1,5	68,119	0,0023529	2,4769
NPEPPS	E9PLK3	Puromycin-sensitive	14	18,4	18,4	102,99	0	24,402
NNT	Q13423	NAD(P) transhydrogenase,	23	25,7	25,7	113,89	0	63,812
NME1-NME2	Q32Q12	Nucleoside diphosphate kinase	16	73,3	73,3	32,642	0	63,864
NLRX1	Q86UT6	NLR family member X1	4	3,7	3,7	107,61	0	5,3986
NIT2	F8WF70	Omega-amidase NIT2	2	29,3	29,3	10,199	0	4,1495
NIPSNAP3A	Q9UFN0	Protein NipSnap homolog 3A	5	21,1	21,1	28,466	0	7,3073
NID1	P14543	Nidogen-1	11	10	10	136,38	0	59,11
NHLRC2	Q8NBF2	NHL repeat-containing protein 2	6	10,7	10,7	79,443	0	13,249
NEXN	Q0ZGT2	Nexilin	11	17	17	80,657	0	15,799
NEK6	Q5TBH0	Serine/threonine-protein kinase	1	8	8	22,655	0,0060015	2,0437
NEDD8	Q15843	NEDD8	2	34,6	34,6	9,0714	0	5,6694
NDUFV2	E7EPT4	NADH dehydrogenase [ubiquinone] flavoprotein 2,	1	5,2	5,2	27,907	0,009908	1,5593
NDUFS5	O43920	NADH dehydrogenase [ubiquinone] iron-sulfur protein 5	1	11,3	11,3	12,517	0	3,308
NDUFS3	O75489	NADH dehydrogenase [ubiquinone] iron-sulfur protein	2	11,7	11,7	30,241	0	4,9124
NDUFS1	B4DJ81	NADH-ubiquinone oxidoreductase 75 kDa subunit,	1	3,6	3,6	66,921	0,0015987	2,6287
NDUFB10	H3BV16	NADH dehydrogenase [ubiquinone] 1 beta subcomplex	1	9,9	9,9	17,385	0	4,2514
NDUFAB1	H3BNK3	Acyl carrier protein	1	12,6	12,6	12,099	0,0038373	2,2775
NDUFA9	Q16795	NADH dehydrogenase [ubiquinone] 1 alpha subcomplex	3	11,1	11,1	42,509	0	4,8744
NDUFA8	P51970	NADH dehydrogenase [ubiquinone] 1 alpha subcomplex	2	18	18	20,105	0,0067014	1,911
NDUFA4	O00483	Cytochrome c oxidase subunit	2	27,2	27,2	9,3697	0	4,5466

NDUFA13	U3KQP3	NADH dehydrogenase [ubiquinone] 1 alpha subcomplex	2	30,7	30,7	8,5979	0	6,0491
NCKIPSD	Q9NZQ3	NCK-interacting protein with SH3	8	12	12	78,959	0	12,431
NCKAP1L	P55160	Nck-associated protein 1-like	30	32,6	31	128,15	0	88,291
NCKAP1	Q9Y2A7	Nck-associated protein 1	49	46,5	44,9	128,79	0	268,05
NCK2	O43639	Cytoplasmic protein NCK2	9	27,6	25,5	42,915	0	21,716
NBEAL2	Q6ZNJ1	Neurobeachin-like protein 2	23	10,3	10,3	302,51	0	46,862
NAV3	H0YI14	Neuron navigator 3	1	1,1	1,1	87,176	1	-2
NARS	O43776	Asparagine--tRNA ligase,	3	8,2	8,2	62,942	0	4,7981
NAPRT	C9J8U2	Nicotinate	5	16,1	16,1	52,161	0	8,503
NAPG	Q99747	Gamma-soluble NSF attachment	6	19,6	19,6	34,746	0	20,189
NAPA	P54920	Alpha-soluble NSF attachment	20	71,5	71,5	33,232	0	121,57
NAP1L4	A8MXH2	Nucleosome assembly protein 1-	5	32,1	25	18,152	0	7,2028
NAP1L1	F8W118		8	38,8	6,2	24,694	0	56,313
NAP1L1	F8W020	Nucleosome assembly protein 1-	8	48,8	15,9	24,366	0	8,695
NAP1L1	F8W543		7	18	2,4	38,2	1	-2
NACA	F8VZ58	Putative nascent polypeptide-associated complex subunit alpha-	2	20,6	20,6	13,597	0	3,7788
MYO1C	F5H6E2	Unconventional myosin-Ic	13	15,2	15,2	118,99	0	23,498
MYLK	Q15746	Myosin light chain kinase, smooth	18	9,3	9,3	210,71	0	43,633
MYL9	P24844	Myosin regulatory light	10	62,8	29,7	19,827	0	61,63
MYL6	F8W1R7	Myosin light polypeptide 6	8	57,9	8,3	16,29	0	3,9731
MYL6	G8JLA2		8	65,1	17,8	17,089	0	204,72
MYL12A	J3QRS3	Myosin regulatory light chain 12A	11	61	28,8	20,457	0	138,78
MYL1	P05976	Myosin light chain 1/3, skeletal	1	8,2	8,2	21,145	0	17,685
MYH9	P35579	Myosin-9	137	61,2	54	226,53	0	323,31
MYH11	P35749	Myosin-11	14	8,2	0,8	227,34	1	-2
MYH10	P35580	Myosin-10	18	9,5	1,1	229	1	-2
MYDGF	M0QXF7	Myeloid-derived growth factor	2	28,6	28,6	9,5594	0	5,478
MYCT1	H0YDV5	Myc target protein 1	4	23,1	23,1	24,246	0	12,196
MYCBP	A0A087WV05	C-Myc-binding protein	1	15,5	15,5	12,755	0	3,1161
MYADM	Q96S97	Myeloid-associated	5	19,6	19,6	35,273	0	25,86
MTSS1	O43312	Metastasis suppressor protein 1	3	4,8	4,8	82,25	0	4,0911

MTPN	P58546	Myotrophin	8	83,1	83,1	12,895	0	64,594
MTMR12	Q9C0I1	Myotubularin-related protein 12	4	7,5	7,5	86,147	0	6,1191
MTM1	Q13496	Myotubularin	3	5,5	5,5	69,931	0	3,3932
MTHFD1	P11586	C-1-tetrahydrofolate synthase,	7	9	9	101,56	0	11,609
MTDH	Q86UE4	Protein LYRIC	3	7,2	7,2	63,836	0	3,458
MT-CO2	P00403	Cytochrome c oxidase subunit 2	5	26,4	26,4	25,565	0	22,63
MT-CO1	P00395	Cytochrome c oxidase subunit 1	1	1,6	1,6	57,041	0,0092461	1,5803
MTCH2	Q9Y6C9	Mitochondrial carrier homolog 2	5	21,8	21,8	33,331	0	3,9242
MT-ATP6	P00846	ATP synthase subunit a	1	4,4	4,4	24,817	0	5,0892
MTAP	B4DUC8	S-methyl-5-thioadenosine	4	24,3	24,3	33,186	0	15,419
MSRA	E5RJK1	Mitochondrial peptide methionine sulfoxide reductase	1	18,3	18,3	9,3986	0,0072411	1,7155
MSN	P26038	Moesin	53	70,2	52,9	67,819	0	323,31
MRVI1	E9PJ61	Protein MRVI1	2	5,8	5,8	46,633	0	3,8184
MRPS36	P82909	28S ribosomal protein S36,	1	15,5	15,5	11,466	0	4,5864
MPST	P25325	3-mercaptopyruvate	3	13,1	13,1	33,178	0	3,2615
MPP1	Q00013	55 kDa erythrocyte membrane	12	35,6	35,6	52,296	0	57,06
MPO	P05164	Myeloperoxidase	3	4,7	4,7	83,868	0	6,8465
MPL	Q5JUY5	Thrombopoietin receptor	2	4,3	4,3	70,558	0	5,0248
MPDU1	J3QS48	Mannose-P-dolichol utilization	2	23,8	23,8	10,978	0,0066914	1,9103
MOB3C	X6R3L3	MOB kinase activator 3C	3	11,2	11,2	31,725	0	6,0074
MOB2	A0A087WVS8	MOB kinase activator 2	2	7,7	7,7	26,575	0,0045386	2,1633
MOB1B	Q7L9L4	MOB kinase activator 1B	2	10,6	10,6	25,091	0	5,5762
MMRN1	Q13201	Multimerin-1	37	35,3	35,3	138,11	0	280,56
MLEC	Q14165	Malectin	11	45,9	45,9	32,233	0	27,626
MLC1	Q15049	Membrane protein MLC1	3	11,4	11,4	41,141	0	3,3792
MIF	P14174	Macrophage migration inhibitory	2	17,4	17,4	12,476	0	4,9935
MICAL1	Q8TDZ2	Protein-methionine sulfoxide	2	2,3	2,3	117,87	0	2,8913
MGST2	Q99735	Microsomal glutathione S-	1	9,5	9,5	16,62	0	2,9261
MGLL	A0A0C4DFN3	Monoglyceride lipase	15	65,2	65,2	34,292	0	96,549
MFSD1	H7C5U3	Major facilitator superfamily domain-containing protein 1	1	18,8	18,8	6,8219	0,0045558	2,2103
METTL7A	F8VQX6	Methyltransferase-like protein 7A	4	23,9	23,9	20,662	0	13,259

METAP1	P53582	Methionine aminopeptidase 1	4	19,4	19,4	43,215	0	17,872
MEMO1	Q9Y316	Protein MEMO1	1	5,1	5,1	33,733	0,0030936	2,3523
ME2	A0A1W2PPH1	NAD-dependent malic enzyme,	14	30,2	30,2	61,221	0	74,228
ME1	P48163	NADP-dependent malic enzyme	3	6,5	6,5	64,149	0	5,7434
MDH2	P40926	Malate dehydrogenase,	16	61,5	61,5	35,503	0	77,363
MDH1	P40925	Malate dehydrogenase,	11	42,5	42,5	36,426	0	68,138
MCU	Q8NE86	Calcium uniporter protein,	6	24,2	24,2	39,866	0	24,137
MCTS1	Q9ULC4	Malignant T-cell-amplified	2	20,4	20,4	20,555	0	5,4706
MCEMP1	M0QYG6	Mast cell-expressed membrane	2	13,9	13,9	14,453	0	4,7793
MBD2	Q9UBB5	Methyl-CpG-binding domain	1	2,9	2,9	43,254	1	-2
MAPRE2	Q15555	Microtubule-associated protein RP/EB family member 2	15	52,6	50,8	37,031	0	104,77
MAPRE1	Q15691	Microtubule-associated protein RP/EB family member 1	12	47	47	29,999	0	53,105
MAPKAPK3	C9J8E1	MAP kinase-activated protein	3	11,1	11,1	37,389	0	9,0627
MAPK14	B4E0K5	Mitogen-activated protein kinase	7	29,7	29,7	32,357	0	22,13
MAPK1	P28482	Mitogen-activated protein kinase	14	46,4	46,4	41,389	0	32,848
MAP2K3	E9PRZ0	Dual specificity mitogen-activated protein kinase kinase 3	3	16,9	16,9	18,212	0	3,0636
MAP2K2	P36507	Dual specificity mitogen-activated protein kinase kinase 2	5	15,2	15,2	44,424	0	13,483
MAOB	P27338	Amine oxidase [flavin-containing]	17	39,4	39,4	58,762	0	68,181
MANF	P55145	Mesencephalic astrocyte-derived neurotrophic factor	2	18,7	18,7	20,7	0	7,8679
MAGED2	Q9UNF1	Melanoma-associated antigen D2	2	2,5	2,5	64,953	0,0038226	2,2571
M6PR	P20645	Cation-dependent mannose-6-	3	15,9	15,9	30,993	0	13,73
LYZ	F8VV32	Lysozyme	1	11,5	11,5	11,488	0	3,1563
LYPLA2	O95372	Acyl-protein thioesterase 2	3	22,5	22,5	24,737	0	8,2575
LYPLA1	A0A087X1K9	Acyl-protein thioesterase 1	3	28,9	28,9	17,981	0	8,619
LYN	P07948	Tyrosine-protein kinase Lyn	26	55,1	53,5	58,573	0	137,9
LY75	O60449	Lymphocyte antigen 75	2	2	2	198,31	0,0099361	1,5721
LY6G6F	Q5SQ64	Lymphocyte antigen 6 complex	10	32,7	32,7	32,464	0	45,166

LTBP1	Q14766	Latent-transforming growth factor beta-binding protein 1	41	33,6	33,6	186,79	0	323,31
LTA4H	P09960	Leukotriene A-4 hydrolase	4	9,7	9,7	69,284	0	5,0382
LST1	F2Z2M1	Leukocyte-specific transcript 1	1	18,2	18,2	7,3877	0	3,4389
LSM3	P62310	U6 snRNA-associated Sm-like	1	11,8	11,8	11,845	0,0031128	2,4163
LSM1	O15116	U6 snRNA-associated Sm-like	2	17,3	17,3	15,179	0	4,0036
LRRC8D	Q7L1W4	Volume-regulated anion channel	1	2	2	98,2	0	2,9762
LRRC59	Q96AG4	Leucine-rich repeat-containing	6	24,8	24,8	34,93	0	13,425
LRRC57	H3BSW0	Leucine-rich repeat-containing	4	31,1	31,1	16,324	0	5,8313
LRRC47	Q8N1G4	Leucine-rich repeat-containing	4	8,7	8,7	63,472	0	6,4852
LRRC32	Q14392	Leucine-rich repeat-containing	10	20,1	20,1	71,978	0	50,298
LRBA	E9PEM5		29	14,4	0	286,97	0	3,1027
LRBA	P50851	Lipopolysaccharide-responsive and beige-like anchor protein	31	14,5	0	319,1	0	79,2
LPP	A0A087WZF1	Lipoma-preferred partner	1	3,9	3,9	63,382	0	5,0133
LOX	P28300	Protein-lysine 6-oxidase	1	4,8	4,8	46,944	0	3,1157
LMAN2	Q12907	Vesicular integral-membrane	8	30,1	30,1	40,228	0	18,874
LMAN1	P49257	Protein ERGIC-53	4	14,3	14,3	57,548	0	11,412
LIMS2	Q7Z4I7	LIM and senescent cell antigen-like-containing domain protein 2	10	27	4,4	38,916	1	-2
LIMS1	P48059	LIM and senescent cell antigen-like-containing domain protein 1	28	77,2	60	37,251	0	149,55
LHFPL2	Q6ZUX7	Lipoma HMGIC fusion partner-	4	19,7	19,7	24,486	0	23,281
LGALSL	Q3ZCW2	Galectin-related protein	8	66,3	66,3	18,986	0	29,012
LGALS12	Q96DT0	Galectin-12	1	3,6	3,6	37,541	0,0038256	2,2583
LGALS1	P09382	Galectin-1	5	45,2	45,2	14,716	0	10,251
LDHB	P07195	L-lactate dehydrogenase B chain	20	55,4	55,4	36,638	0	233,52
LDHA	P00338	L-lactate dehydrogenase A chain	21	60,2	56,6	36,688	0	145,62
LCP2	A0A0D9SGG1	Lymphocyte cytosolic protein 2	11	23,5	23,5	60,143	0	25,093
LBP	P18428	Lipopolysaccharide-binding	1	2,3	2,3	53,383	0,0045697	2,2223
LAT	O43561	Linker for activation of T-cells	2	9,9	9,9	27,929	0	7,2222
LASP1	Q14847	LIM and SH3 domain protein 1	9	35,2	35,2	29,717	0	31,809
LAP3	P28838	Cytosol aminopeptidase	13	31,4	31,4	56,166	0	25,015

LANCL1	F8WDS9	LanC-like protein 1	2	26,8	26,8	9,2824	0	3,0407
LAMTOR5	R4GMU8	Ragulator complex protein	2	55,7	55,7	8,2153	0	13,328
LAMTOR3	Q9UHA4	Ragulator complex protein	4	54,8	54,8	13,623	0	65,274
LAMTOR2	Q9Y2Q5	Ragulator complex protein	4	33,6	33,6	13,507	0	4,9698
LAMTOR1	Q6IAA8	Ragulator complex protein	5	49,7	49,7	17,745	0	21,504
LAMP2	P13473	Lysosome-associated membrane	2	4,1	4,1	44,96	0	4,1463
LAMP1	P11279	Lysosome-associated membrane	6	13,4	13,4	44,882	0	24,885
KRT9	P35527	Keratin, type I cytoskeletal 9	34	67,3	67,3	62,064	0	323,31
KRT73	CON__Q32MB	Keratin, type II cytoskeletal 73	6	7,8	2,2	58,923	0	20,123
KRT6C	P48668	Keratin, type II cytoskeletal 6C	24	36,2	0	60,024	0	53,983
KRT5	CON__P13647	Keratin, type II cytoskeletal 5	29	39	7,8	62,378	0	35,361
KRT4	CON__P19013	Keratin, type II cytoskeletal 4	4	6,7	2	63,91	0	3,0284
KRT2	CON__P35908v	Keratin, type II cytoskeletal 2	51	76,4	4,7	65,432	0	323,31
KRT19	CON__P08727	Keratin, type I cytoskeletal 19	8	16,8	3,2	44,091	1	-2
KRT19	K7EMS3		3	11,6	6,1	20,186	0,00080064	2,6585
KRT17	CON__Q04695	Keratin, type I cytoskeletal 17	16	28,7	2,8	48,105	0	5,6601
KRT16	CON__P08779	Keratin, type I cytoskeletal 16	25	52,4	35,9	51,267	0	104,25
KRT14	P02533	Keratin, type I cytoskeletal 14	24	47	17,8	51,561	0	61,577
KRT10	CON__P13645	Keratin, type I cytoskeletal 10	37	61,7	48,9	59,51	0	323,31
KRT1	P04264	Keratin, type II cytoskeletal 1	46	58,1	42,5	66,038	0	323,31
KRAS	P01116	GTPase KRas	5	28	7,9	21,656	0	9,9178
KPNB1	Q14974	Importin subunit beta-1	18	26	26	97,169	0	57,031
KPNA6	O60684	Importin subunit alpha-7	2	4,7	4,7	60,029	0	3,5885
KPNA4	O00629	Importin subunit alpha-3	2	4	4	57,886	0,0072622	1,7552
KLC1	G5E9S8	Kinesin light chain 1	2	4,9	4,9	62,506	0	3,5973
KIRREL	Q5W0G0	Kin of IRRE-like protein 1	1	1,6	1,6	63,099	1	-2
KIF5B	P33176	Kinesin-1 heavy chain	4	5,6	5,6	109,68	0	6,1621
KIF2A	O00139	Kinesin-like protein KIF2A	19	26,9	26,9	79,954	0	69,001
KIF27	Q86VH2	Kinesin-like protein KIF27	1	0,6	0,6	160,28	0,0074074	1,87
KIF20B	Q96Q89	Kinesin-like protein KIF20B	1	0,5	0,5	210,63	0,0079422	1,6955
KIAA0513	O60268	Uncharacterized protein	4	12,9	12,9	46,638	0	6,4799
KCNK6	Q9Y257	Potassium channel subfamily K	2	7,7	7,7	33,747	0	3,6721

KCNA2	P16389	Potassium voltage-gated channel subfamily A member 2	2	4,8	4,8	56,716	0,0031056	2,3999
KATNAL2	K7EM02	Katanin p60 ATPase-containing	1	9,4	9,4	13,999	0,0015848	2,5626
KALRN	C9J1B4		5	52,1	14,9	21,064	0	7,5889
KALRN	H7BXZ5	Kalirin	17	7,7	5,2	336,17	0	47,04
JAM3	Q9BX67	Junctional adhesion molecule C	9	37,7	37,7	35,02	0	54,301
ITGB6	A0A087WXP3	Integrin beta	1	1,3	1,3	75,849	0,0092395	1,5774
ITGB5	H7C4W1	Integrin beta	1	5,3	5,3	28,96	0,0023493	2,4596
ITGB3	P05106	Integrin beta-3	51	66	66	87,057	0	323,31
ITGB1	P05556	Integrin beta-1	28	39,8	39,8	88,414	0	239,9
ITGAV	P06756	Integrin alpha-V	2	1,9	1,9	116,04	0	4,349
ITGA6	P23229	Integrin alpha-6	51	52,7	52,7	126,6	0	323,31
ITGA5	P08648	Integrin alpha-5	11	14,9	14,9	114,54	0	48,667
ITGA2B	P08514	Integrin alpha-IIb	53	50,2	50,2	113,38	0	323,31
ITGA2	E7ESP4		26	34,3	2,1	102,83	0	42,063
ITGA2	P17301	Integrin alpha-2	31	34,6	9	129,29	0	211,31
ITFG3	H7C2U8	Protein ITFG3	1	6,2	6,2	20,934	0,0072886	1,7682
IST1	H3BMU1	IST1 homolog	4	18	18	23,375	0,0045524	2,2002
ISOC1	Q96CN7	Isochorismatase domain-	6	28,5	28,5	32,236	0	13,844
IQGAP2	Q13576	Ras GTPase-activating-like	29	22,7	22,7	180,58	0	64,857
IPO7	O95373	Importin-7	5	7,4	7,4	119,52	0	14,381
IPO5	H0Y8C6	Importin-5	5	5	5	123,75	0	9,2841
INPP5A	Q14642	Type I inositol 1,4,5-	4	14,8	14,8	47,819	0	9,4269
INPP4B	E9PHC0	Type II inositol 3,4-bisphosphate	2	3,4	3,4	77,516	0	3,8778
INF2	Q27J81	Inverted formin-2	31	33,9	33,9	135,62	0	200,21
IMPA1	H0YBL1	Inositol monophosphatase 1	5	25,7	25,7	28,529	0	11,242
IMMT	B9A067	MICOS complex subunit MIC60	10	16,3	16,3	78,973	0	15,516
ILK	A0A0A0MTH3	Integrin-linked protein kinase	34	68,1	68,1	54,611	0	323,31
IL1RL1	E9PC41	Interleukin-1 receptor-like 1	1	4,5	4,5	35,597	0,0072993	1,7751
IGLL5	P0DOY3	Ig lambda-1 chain C regions	3	37,7	37,7	11,265	0	15,813
IGKC	P01834	Ig kappa chain C region	4	48,6	48,6	11,765	0	25,403
IGHM	A0A1B0GUU9	Ig mu chain C region	1	3,2	3,2	51,923	0,0023456	2,4506
IGHG3	A0A286YES1	Ig gamma-3 chain C region	7	17,7	2,7	49,021	0	3,1301

IGHG2	A0A286YEY4	Ig gamma-2 chain C region	8	27,1	10,4	43,805	0	20,209
IGHG1	P01857	Ig gamma-1 chain C region	11	50,3	26,7	36,105	0	47,347
IGHA1	A0A286YEY1	Ig alpha-1 chain C region	6	22,6	22,6	42,848	0	15,222
IDH3A	H0YKD0	Isocitrate dehydrogenase [NAD] subunit alpha, mitochondrial	2	23,9	23,9	12,135	0,003096	2,3578
IDH2	P48735	Isocitrate dehydrogenase [NADP],	24	52,4	52,4	50,909	0	134,48
IDH1	O75874	Isocitrate dehydrogenase [NADP]	13	38,2	36	46,659	0	26,703
ICAM2	J3QRT5	Intercellular adhesion molecule 2	7	28,7	28,7	28,181	0	16,444
HYOU1	A0A087X054	Hypoxia up-regulated protein 1	8	13,1	13,1	104,78	0	27,342
HUWE1	Q7Z6Z7	E3 ubiquitin-protein ligase	3	0,8	0,8	481,89	0	3,2346
HSPE1	P61604	10 kDa heat shock protein,	8	74,5	74,5	10,932	0	15,674
HSPD1	P10809	60 kDa heat shock protein,	23	48,7	48,7	61,054	0	133,07
HSPB1	P04792	Heat shock protein beta-1	16	63,9	63,9	22,782	0	91,389
HSPA9	P38646	Stress-70 protein, mitochondrial	15	29,9	28,9	73,68	0	50,813
HSPA8	P11142	Heat shock cognate 71 kDa	39	60,7	52,8	70,897	0	323,31
HSPA6	P17066	Heat shock 70 kDa protein 6	12	16,3	3	71,027	0	13,626
HSPA5	P11021	78 kDa glucose-regulated protein	37	58,6	55	72,332	0	164,99
HSPA4	P34932	Heat shock 70 kDa protein 4	11	16	16	94,33	0	32,111
HSPA1B	A0A0G2JIW1	Heat shock 70 kDa protein 1B	28	48,4	27,9	70,108	0	124,61
HSP90B1	P14625	Endoplasmin	35	44,5	42,7	92,468	0	133,39
HSP90AB2P	Q58FF8	Putative heat shock protein HSP	9	19,2	4,5	44,348	0	6,5596
HSP90AB1	P08238	Heat shock protein HSP 90-beta	28	35,4	18,2	83,263	0	73,165
HSP90AA1	P07900	Heat shock protein HSP 90-alpha	36	37,8	25,1	84,659	0	177,74
HSF2BP	C9JSF2	Heat shock factor 2-binding	1	3,5	3,5	25,387	0,0072833	1,763
HSD17B4	A0A2R8YD50	Peroxisomal multifunctional	20	36,3	36,3	76,837	0	95,605
HSD17B12	Q53GQ0	Very-long-chain 3-oxoacyl-CoA	7	28,2	28,2	34,324	0	15,379
HSD17B10	Q99714	3-hydroxyacyl-CoA	5	30,7	30,7	26,923	0	14,748
HRNR	CON__Q86YZ3	Hornerin	3	6,5	6,5	282,39	0	18,31
HPX	P02790	Hemopexin	2	9,7	9,7	51,676	0	4,4191
HPSE	Q9Y251	Heparanase	14	27,3	27,3	61,148	0	41,6
HPRT1	P00492	Hypoxanthine-guanine	6	29,4	26,1	24,579	0	11,289
HPCAL1	P37235	Hippocalcin-like protein 1	8	48,7	48,7	22,313	0	20,058
HP	A0A087WU08	Haptoglobin	2	7,8	7,8	31,408	0	2,7587

HNRNPK	P61978	Heterogeneous nuclear	9	24	24	50,976	0	19,147
HNRNPF	A0A1B0GW42	Heterogeneous nuclear	1	13,3	13,3	11,079	0,007948	1,702
HMHA1	K7ES98	Minor histocompatibility protein	13	14,9	14,9	125,05	0	33,697
HMGCL	P35914	Hydroxymethylglutaryl-CoA lyase,	3	10,8	10,8	34,36	0	3,3278
HLA-G	Q8MH48	HLA class I histocompatibility	2	6,8	3	38,254	0,0045351	2,1369
HLA-E	Q6DU44	HLA class I histocompatibility	6	20,7	8,9	40,057	0	4,6453
HLA-C	A2AEA2	HLA class I histocompatibility antigen, Cw-7 alpha chain	13	35,5	0	41,301	0	48,946
HLA-C	P30508	HLA class I histocompatibility antigen, Cw-12 alpha chain	17	47,8	0	40,885	0	20,631
HLA-B	P01889	HLA class I histocompatibility antigen, B-7 alpha chain	18	40,1	3,6	40,46	0,0073692	1,8491
HLA-B	P03989	HLA class I histocompatibility antigen, B-27 alpha chain	17	41,4	0	40,428	0	41,137
HLA-B	P10319	HLA class I histocompatibility antigen, B-58 alpha chain	18	59,4	1,9	40,337	1	-2
HLA-B	P18463	HLA class I histocompatibility antigen, B-37 alpha chain	19	54,1	3	40,456	0	10,366
HLA-B	P30460	HLA class I histocompatibility antigen, B-8 alpha chain	19	48,9	3	40,33	0,0031032	2,3782
HLA-B	P30462	HLA class I histocompatibility antigen, B-14 alpha chain	20	51,7	0	40,358	0	9,9466
HLA-B	P30466	HLA class I histocompatibility antigen, B-18 alpha chain	19	55,2	3,9	40,274	0	5,6854
HLA-B	P30480	HLA class I histocompatibility antigen, B-42 alpha chain	20	48,3	0	40,332	0	28,126
HLA-B	P30481	HLA class I histocompatibility antigen, B-44 alpha chain	18	48,6	4,4	40,481	0	42,699
HLA-B	P30483	HLA class I histocompatibility antigen, B-45 alpha chain	18	51,9	0	40,414	0	5,198
HLA-B	P30490	HLA class I histocompatibility antigen, B-52 alpha chain	19	58,3	0	40,521	0	12,228

HLA-B	P30491	HLA class I histocompatibility antigen, B-53 alpha chain	20	61,3	0	40,495	0	216,21
HLA-B	Q04826	HLA class I histocompatibility antigen, B-40 alpha chain	18	43,9	0	40,505	0	5,7105
HLA-B	Q29940	HLA class I histocompatibility antigen, B-59 alpha chain	20	52,2	0	40,584	0	4,6076
HLA-B	Q95365	HLA class I histocompatibility antigen, B-38 alpha chain	20	51,4	0	40,416	0	5,3792
HLA-A	A0A0G2JIF2	HLA class I histocompatibility antigen, A-36 alpha chain	20	57,1	5,1	41,373	0	4,1655
HLA-A	A0A140T9I0	HLA class I histocompatibility antigen, A-2 alpha chain	26	64,9	4,9	40,79	0	323,31
HLA-A	A0A140T975	HLA class I histocompatibility antigen, A-32 alpha chain	19	64,9	0	34,321	0,0072359	1,7117
HLA-A	Q5SRN5	HLA class I histocompatibility antigen, A-3 alpha chain	23	62,5	6,5	41,368	0	40,834
HLA-A	P05534	HLA class I histocompatibility antigen, A-24 alpha chain	24	63,6	3	40,688	0	97,324
HLA-A	P10316	HLA class I histocompatibility antigen, A-69 alpha chain	26	65,8	0	40,976	0	42,583
HLA-A	P13746	HLA class I histocompatibility antigen, A-11 alpha chain	21	60,3	3	40,936	1	-2
HLA-A	P16190	HLA class I histocompatibility antigen, A-33 alpha chain	18	52,1	0	40,891	0	4,4363
HLA-A	P30447	HLA class I histocompatibility antigen, A-23 alpha chain	23	60,8	0	40,732	0	12,28
HLA-A	P30453	HLA class I histocompatibility antigen, A-34 alpha chain	21	56,2	0	41,054	0	25,21
HK1	P19367	Hexokinase-1	28	34	34	102,48	0	75,716
HIST1H4A	P62805	Histone H4	3	33	33	11,367	0	6,0817
HINT2	Q9BX68	Histidine triad nucleotide-binding protein 2, mitochondrial	2	21,5	21,5	17,162	0	3,0259
HEPHL1	Q6MZM0	Hephaestin-like protein 1	1	1,4	1,4	131,6	0,0074019	1,8662

HDHD2	Q9H0R4	Haloacid dehalogenase-like hydrolase domain-containing	2	8,1	8,1	28,536	0,0052671	2,1029
HCLS1	E7EVW7	Hematopoietic lineage cell-	4	9,6	9,6	49,704	0	6,2567
hCG_1994888	A0A024R161	Guanine nucleotide-binding	1	11,8	11,8	16,499	0,0086269	1,6655
HBD	P02042	Hemoglobin subunit delta	8	48,3	8,8	16,055	0	2,7197
HBB	P68871	Hemoglobin subunit beta	14	95,2	61,9	15,998	0	108,44
HBA1	P69905	Hemoglobin subunit alpha	12	91,5	67,6	15,257	0	131,08
HARS	P12081	Histidine--tRNA ligase,	6	11,4	11,4	57,41	0	4,1474
HADHB	P55084	Trifunctional enzyme subunit	13	32,9	32,9	51,294	0	31,174
HADHA	H0YFD6	Trifunctional enzyme subunit	22	37,9	37,9	86,371	0	63,983
HADH	A0A0A0MSE2	Hydroxyacyl-coenzyme A dehydrogenase, mitochondrial	6	24,6	24,6	34,594	0	10,516
HACD4	Q5VWC8	Very-long-chain (3R)-3-hydroxyacyl-CoA dehydratase 4	2	9,1	9,1	27,52	0,0023585	2,511
GUCY1B3	B7Z685	Guanylate cyclase soluble subunit	4	9,8	9,8	62,761	0	7,9504
GUCY1A3	Q02108	Guanylate cyclase soluble subunit	4	6,2	6,2	77,452	0	6,8615
GTPBP2	Q9BX10	GTP-binding protein 2	11	21,1	21,1	65,768	0	56,575
GTPBP1	O00178	GTP-binding protein 1	1	2,4	2,4	72,453	0,0030817	2,3347
GSTP1	P09211	Glutathione S-transferase P	9	66,2	66,2	23,356	0	138,83
GSTO1	P78417	Glutathione S-transferase omega-	16	64,3	64,3	27,566	0	82,907
GSTM3	P21266	Glutathione S-transferase Mu 3	3	15,1	11,1	26,559	0	5,1707
GSTM2	E9PHN7	Glutathione S-transferase Mu 2	4	26,5	9,4	21,362	0	4,6293
GSTM1	B9ZVX7	Glutathione S-transferase Mu 1	3	26,2	6	17,39	0	5,4436
GSTK1	Q9Y2Q3	Glutathione S-transferase kappa	8	44,2	44,2	25,497	0	19,71
GSS	A0A2R8Y430	Glutathione synthetase	5	12,4	12,4	49,976	0	10,209
GSR	P00390	Glutathione reductase,	11	30,8	30,8	56,256	0	36,925
GSPT1	H3BR35	Eukaryotic peptide chain release factor GTP-binding subunit ERF3A	4	9,5	9,5	52,832	0	7,3555
GSN	A0A0A0MS51	Gelsolin	48	60,6	40	82,525	0	323,31
GSN	Q5T0I0		16	62,7	3,5	28,953	0	3,3049
GRK6	D6RHX8	G protein-coupled receptor	3	9,3	9,3	54,483	0	3,4062
GRHPR	Q9UBQ7	Glyoxylate	7	29,3	29,3	35,668	0	39,653
GRB2	P62993	Growth factor receptor-bound	13	59,9	59,9	25,206	0	38,163

GRAP2	O75791	GRB2-related adapter protein 2	8	24,8	24,8	37,909	0	8,8093
GPX4	K7ERP4	Glutathione peroxidase	5	41,3	41,3	17,629	0	13,26
GPX1	P07203	Glutathione peroxidase 1	10	57,6	57,6	22,088	0	61,961
GPS1	C9JFE4	COP9 signalosome complex	3	8,1	8,1	53,371	0	5,2209
GPI	P06744	Glucose-6-phosphate isomerase	19	44,1	44,1	63,146	0	213,99
GPD2	P43304	Glycerol-3-phosphate dehydrogenase, mitochondrial	22	40,2	40,2	80,852	0	75,381
GP9	P14770	Platelet glycoprotein IX	6	35,6	35,6	19,046	0	107,71
GP6	Q9HCN6	Platelet glycoprotein VI	5	22,4	22,4	36,866	0	13,161
GP5	P40197	Platelet glycoprotein V	16	38	38	60,958	0	143,87
GP1BB	P13224	Platelet glycoprotein Ib beta	6	24,8	24,8	21,717	0	30,407
GP1BA	A0A0C4DGZ8	Platelet glycoprotein Ib alpha	21	28,4	28,4	68,954	0	175,32
GOT2	P00505	Aspartate aminotransferase,	7	21,6	21,6	47,517	0	23,191
GOT1	P17174	Aspartate aminotransferase,	1	3,6	3,6	46,247	0,0072727	1,7617
GOLT1B	G3V1U5	Vesicle transport protein GOT1B	1	20,3	20,3	8,2139	0	6,3336
GOLPH3L	Q5T5I6	Golgi phosphoprotein 3-like	1	5,6	5,6	31,128	0,0067214	1,9444
GOLPH3	Q9H4A6	Golgi phosphoprotein 3	1	5	5	33,81	0	2,753
GNG5	P63218	Guanine nucleotide-binding protein G(I)/G(S)/G(O) subunit	2	23,5	23,5	7,3184	0,0015911	2,6084
GNG11	P61952	Guanine nucleotide-binding protein G(I)/G(S)/G(O) subunit	5	56,2	56,2	8,4807	0	11,954
GNB5	H0YNW7		2	27	15	11,429	0	12,099
GNB5	O14775	Guanine nucleotide-binding	2	7,8	4,8	43,566	0,0079537	1,706
GNB4	Q9HAV0	Guanine nucleotide-binding	14	60,6	34,4	37,567	0	99,08
GNB2	P62879	Guanine nucleotide-binding protein G(I)/G(S)/G(T) subunit	12	50,6	21,5	37,331	0	55,912
GNB1	P62873	Guanine nucleotide-binding protein G(I)/G(S)/G(T) subunit	14	60,9	30,6	37,377	0	323,31
GNAZ	P19086	Guanine nucleotide-binding protein G(z) subunit alpha	17	50,4	48,2	40,923	0	77,497
GNAS	P63092	Guanine nucleotide-binding protein G(s) subunit alpha	6	16,5	13,7	45,664	0	7,364

GNAQ	P50148	Guanine nucleotide-binding protein G(q) subunit alpha	23	64,6	55,2	42,142	0	162,24
GNAI3	P08754	Guanine nucleotide-binding protein G(k) subunit alpha	10	28,8	6,5	40,532	0	3,3684
GNAI2	P04899	Guanine nucleotide-binding protein G(i) subunit alpha-2	23	65,9	37,2	40,45	0	291,24
GNAI1	P63096	Guanine nucleotide-binding protein G(i) subunit alpha-1	11	33,1	4,2	40,361	0,009922	1,5608
GNA14	O95837	Guanine nucleotide-binding protein subunit alpha-14	4	13,2	3,7	41,57	0,0023622	2,5191
GNA13	Q14344	Guanine nucleotide-binding protein subunit alpha-13	19	50,7	48,5	44,049	0	78,817
GMPR	P36959	GMP reductase 1	11	42,6	42,6	37,418	0	30,226
GMPPB	Q9Y5P6	Mannose-1-phosphate	2	8,9	8,9	39,834	0,0015949	2,6193
GMFG	O60234	Glia maturation factor gamma	6	57,7	32,4	16,801	0	44,17
GLUD1	P00367	Glutamate dehydrogenase 1,	8	20,4	20,4	61,397	0	17,467
GLTP	F5GZ49	Glycolipid transfer protein	1	22,4	22,4	7,2826	0	3,179
GLS	H7BZD1	Glutaminase kidney isoform,	1	13,2	13,2	12,622	0	3,0883
GLRX3	O76003	Glutaredoxin-3	3	15,2	15,2	37,432	0	9,715
GLOD4	F6TLX2	Glyoxalase domain-containing	5	11,4	11,4	54,719	0	10,581
GLO1	Q04760	Lactoylglutathione lyase	5	27,7	27,7	20,777	0	8,7636
GLIPR2	Q9H4G4	Golgi-associated plant pathogenesis-related protein 1	3	27,9	27,9	17,218	0	5,3175
GLB1	P16278	Beta-galactosidase	5	12,7	12,7	76,074	0	11,627
GIT1	J3QLH1	ARF GTPase-activating protein	2	11	11	25,736	0,0045455	2,183
GIPC3	A0A2R8Y651	PDZ domain-containing protein	2	2,7	2,7	143,19	0	7,9808
GDI2	P50395	Rab GDP dissociation inhibitor	29	66,5	44,5	50,663	0	154,7
GDI1	P31150	Rab GDP dissociation inhibitor	25	60,6	38,7	50,582	0	91,898
GCLM	P48507	Glutamate--cysteine ligase	4	21,9	21,9	30,727	0	12,493
GCA	H7C2Z6	Grancalcin	2	15,8	15,8	16,712	0	3,429
GBE1	Q04446	1,4-alpha-glucan-branching	5	11,7	11,7	80,473	0	14,231
GARS	P41250	Glycine--tRNA ligase	9	13,4	13,4	83,165	0	50,47

GAPVD1	B0QZ65	GTPase-activating protein and VPS9 domain-containing protein	2	3,8	3,8	56,461	0,009299	1,6285
GAPDH	P04406	Glyceraldehyde-3-phosphate	25	85,7	85,7	36,053	0	323,31
GANAB	Q14697	Neutral alpha-glucosidase AB	24	35,9	35,9	106,87	0	94,414
G6PD	P11413	Glucose-6-phosphate 1-	26	55,3	55,3	59,256	0	71,379
G6B	O95866	Protein G6b	11	46,1	5	26,163	0	10,85
FYN	P06241	Tyrosine-protein kinase Fyn	11	27,9	20,5	60,761	0	27,797
FYB	O15117	FYN-binding protein	19	31	31	85,386	0	75,906
FN3K	Q9H479	Fructosamine-3-kinase	7	26,9	26,9	35,171	0	11,996
FN1	P02751	Fibronectin	2	1,3	1,3	262,62	0	6,1184
FLOT2	E7EMK3	Flotillin-2	5	14,9	14,9	53,137	0	11,463
FLOT1	O75955	Flotillin-1	5	15,9	15,9	47,355	0	8,8203
FLNC	Q14315	Filamin-C	16	5,1	0,3	291,02	0	8,6815
FLNB	O75369	Filamin-B	16	5,5	1,5	278,16	0	5,4494
FLNA	F8WE98		40	65,4	1,7	66,591	1	-2
FLNA	P21333	Filamin-A	175	75,3	0,6	280,74	0	323,31
FLNA	Q60FE5		173	75,3	0,6	278,22	0	15,728
FLII	Q13045	Protein flightless-1 homolog	8	8,8	8,8	144,75	0	19,121
FLG2	CON__Q5D862	Filaggrin-2	1	0,5	0,5	248,07	0,0073314	1,8058
FLG	CON__P20930	Filaggrin	1	1,7	1,7	435,16	0,0052751	2,1066
FKBP3	Q00688	Peptidyl-prolyl cis-trans	3	17,4	17,4	25,177	0	4,8977
FKBP2	P26885	Peptidyl-prolyl cis-trans	3	23,9	23,9	15,649	0	2,7641
FKBP1A	P62942	Peptidyl-prolyl cis-trans	5	73,1	73,1	11,951	0	21,112
FIS1	Q9Y3D6	Mitochondrial fission 1 protein	1	10,5	10,5	16,937	0	42,143
FHOD1	Q9Y613	FH1/FH2 domain-containing	33	30,1	30,1	126,55	0	117,04
FHL1	Q5JXI8	Four and a half LIM domains	14	63,8	63,8	29,16	0	63,636
FH	P07954	Fumarate hydratase,	8	20,8	20,8	54,636	0	18,488
FGG	P02679	Fibrinogen gamma chain	23	62,5	62,5	51,511	0	296,52
FGB	P02675	Fibrinogen beta chain	30	72,1	72,1	55,928	0	323,31
FGA	P02671	Fibrinogen alpha chain	31	38,5	38,5	94,972	0	309,24
FERMT3	F5H3I6		7	71,6	39,5	9,3926	0	10,609
FERMT3	Q86UX7	Fermitin family homolog 3	52	78	74,1	75,952	0	323,31
FDPS	P14324	Farnesyl pyrophosphate synthase	3	9,8	9,8	48,275	0	7,1538

FCGR2A	P12318	Low affinity immunoglobulin gamma Fc region receptor II-a	6	24	24	35	0	59,295
FCER1G	P30273	High affinity immunoglobulin epsilon receptor subunit gamma	4	33,7	33,7	9,6674	0	7,4916
FASN	A0A0U1RQF0	Fatty acid synthase	20	12,2	12,2	273,2	0	48,928
FAM65C	Q96MK2	Protein FAM65C	1	1,3	1,3	105,29	0,004542	2,1639
FAM63A	Q8N5J2	Protein FAM63A	9	28,8	26,9	51,777	0	30,651
FAM49B	Q9NUQ9	Protein FAM49B	7	28,4	28,4	36,748	0	15,006
FAM26E	Q8N5C1	Protein FAM26E	4	13,9	13,9	35,17	0	7,2795
FAM213B	A0A2P0CTB1	Prostamide/prostaglandin F	2	11,8	11,8	24,278	0	4,4382
FAM177A1	Q8N128	Protein FAM177A1	2	12,7	12,7	23,757	0,0038197	2,2559
FAM162A	Q96A26	Protein FAM162A	3	23,4	23,4	17,342	0	33,835
FAM129A	Q9BZQ8	Protein Niban	1	1,5	1,5	103,13	0	3,3981
FAH	P16930	Fumarylacetoacetase	5	15,8	15,8	46,374	0	16,728
FABP5	Q01469	Fatty acid-binding protein,	2	17	17	15,164	0	8,0993
F5	A0A0A0MRJ7	Coagulation factor V	55	30,1	26,5	252,23	0	272,59
F2RL3	Q96RI0	Proteinase-activated receptor 4	4	9,6	9,6	41,133	0	5,015
F2	P00734	Prothrombin	14	26,5	26,5	70,036	0	62,851
F13A1	P00488	Coagulation factor XIII A chain	45	61,2	61,2	83,266	0	323,31
F11R	Q9Y624	Junctional adhesion molecule A	15	50,8	50,8	32,583	0	144,28
EXOC4	Q96A65	Exocyst complex component 4	4	5,5	5,5	110,5	0	6,4678
EXOC3L4	Q17RC7	Exocyst complex component 3-	3	5,7	5,7	79,895	0	5,504
ETFB	P38117	Electron transfer flavoprotein	4	18	18	27,843	0	8,0293
ETFA	P13804	Electron transfer flavoprotein subunit alpha, mitochondrial	10	42	42	35,079	0	21,154
ETF1	P62495	Eukaryotic peptide chain release	4	12,1	12,1	49,03	0	11,958
ESYT2	H7BXI1	Extended synaptotagmin-2	2	3,1	3,1	98,008	0	3,3386
ESYT1	Q9BSJ8	Extended synaptotagmin-1	17	21,4	21,4	122,85	0	32,495
ESD	P10768	S-formylglutathione hydrolase	7	35,5	35,5	31,462	0	15,565
ESAM	Q96AP7	Endothelial cell-selective	8	33,8	33,8	41,176	0	64,874
ERP44	Q9BS26	Endoplasmic reticulum resident	7	20,4	20,4	46,971	0	17,604
ERP29	P30040	Endoplasmic reticulum resident	5	24,9	24,9	28,993	0	10,241
ERBB2IP	Q96RT1	Protein LAP2	9	7,7	5,9	158,3	0	42,575

ERAP1	Q9NZ08	Endoplasmic reticulum	6	8,2	8,2	107,23	0	19,665
EPS15L1	M0R2S2	Epidermal growth factor receptor substrate 15-like 1	2	3,6	3,6	49,856	0,0038314	2,2627
EPS15	P42566	Epidermal growth factor receptor	4	7,3	7,3	98,655	0	9,6289
ENTHD2	I3L3R3	AP-4 complex accessory subunit	1	4,4	4,4	19,288	1	-2
ENPP4	Q9Y6X5	Bis(5-adenosyl)-triphosphatase	2	4,6	4,6	51,641	0	8,8608
ENOPH1	A0A0C4DGY8	Enolase-phosphatase E1	1	8,1	8,1	16,533	0,0092593	1,5854
ENO2	P09104	Gamma-enolase	11	40,3	30,9	47,268	0	41,891
ENO1	P06733	Alpha-enolase	29	72,1	68,7	47,168	0	323,31
ENDOD1	O94919	Endonuclease domain-containing	9	21,6	21,6	55,016	0	57,478
EML2	K7EII6	Echinoderm microtubule-	3	29,3	29,3	18,888	0	12,814
EMILIN1	Q9Y6C2	EMILIN-1	3	4,5	4,5	106,69	0,0067114	1,9266
ELOVL7	D6RE10	Elongation of very long chain	1	14,1	14,1	8,2937	0,007326	1,8028
ELMO1	Q92556	Engulfment and cell motility	4	7	7	83,829	0	7,5117
EIF5A	I3L397	Eukaryotic translation initiation	9	71,9	71,9	16,019	0	65,828
EIF4G2	H0Y3P2	Eukaryotic translation initiation	5	5,4	5,4	98,117	0	4,3577
EIF4G1	E7EX73	Eukaryotic translation initiation	4	4,2	4,2	158,64	0	5,3753
EIF4E	P06730	Eukaryotic translation initiation	2	8,3	8,3	25,097	0,0023548	2,4772
EIF4A1	P60842	Eukaryotic initiation factor 4A-I	15	37,4	37,4	46,153	0	49,029
EIF3L	B0QY90	Eukaryotic translation initiation	4	10,5	10,5	55,161	0	5,0202
EIF3J	H0YGJ7	Eukaryotic translation initiation	1	11,3	11,3	11,959	0	2,7991
EIF3F	O00303	Eukaryotic translation initiation	2	6,2	6,2	37,563	0,0092791	1,6171
EIF3E	P60228	Eukaryotic translation initiation	2	4,7	4,7	52,22	0,0092725	1,6168
EIF3B	P55884	Eukaryotic translation initiation	2	2,8	2,8	92,48	0	3,6377
EIF2S3	P41091	Eukaryotic translation initiation	7	24,2	24,2	51,109	0	11,725
EIF2S1	P05198	Eukaryotic translation initiation	5	23,5	23,5	36,112	0	15,007
EHD4	Q9H223	EH domain-containing protein 4	15	30,7	20,3	61,174	0	16,34
EHD3	Q9NZN3	EH domain-containing protein 3	39	76,3	48,4	60,886	0	323,31
EHD1	Q9H4M9	EH domain-containing protein 1	37	77,7	53,4	60,626	0	218,88
EFR3A	A0A1B0GUZ7	Protein EFR3 homolog A	3	4	4	96,13	0	3,7137
EFNB1	P98172	Ephrin-B1	3	12,7	12,7	38,006	0	8,1022
EEF2	P13639	Elongation factor 2	4	4,9	4,9	95,337	0	4,0588
EEF1G	P26641	Elongation factor 1-gamma	12	29,5	29,5	50,118	0	39,652

EEF1D	P29692	Elongation factor 1-delta	4	20,3	20,3	31,121	0	33,618
EEF1B2	P24534	Elongation factor 1-beta	3	18,2	18,2	24,763	0	11,476
EEF1A1P5	Q5VTE0	Putative elongation factor 1-	9	28,1	28,1	50,184	0	40,879
ECHS1	P30084	Enoyl-CoA hydratase,	1	4,1	4,1	31,387	0	2,8973
		Delta(3,5)-Delta(2,4)-dienoyl-CoA						
ECH1	Q13011	isomerase, mitochondrial	7	24,1	24,1	35,816	0	39,129
ECE1	B4DKB2	Endothelin-converting enzyme 1	22	37	37	83,573	0	127,66
EBP	Q15125	3-beta-hydroxysteroid-	2	12,2	12,2	26,352	0	4,5898
DYNLL2	Q96FJ2	Dynein light chain 2, cytoplasmic	5	64	38,2	10,35	0	6,4313
DYNLL1	P63167	Dynein light chain 1, cytoplasmic	5	64	38,2	10,366	0	33,071
DYNC1H1	Q14204	Cytoplasmic dynein 1 heavy chain	9	2,2	2,2	532,4	0	10,277
DUSP3	P51452	Dual specificity protein	8	55,1	55,1	20,478	0	31,551
DTD1	Q8TEA8	D-tyrosyl-tRNA(Tyr) deacylase 1	4	20,1	20,1	23,423	0	8,7564
DSTN	P60981	Destrin	9	49,1	44,8	18,506	0	52,249
DST	E9PHM6	Dystonin	1	0,3	0,3	629,78	1	-2
DST	Q5T0V7		1	1,6	1,6	79,167	1	-2
DSP	P15924	Desmoplakin	5	2,4	2,4	331,77	0	8,4754
DPYSL2	A0A1C7CYX9	Dihydropyrimidinase-related	16	35,3	35,3	73,502	0	92,094
DPP3	G3V1D3	Dipeptidyl peptidase 3	3	7,1	7,1	84,304	0	9,3647
DOK3	D6RAM3	Docking protein 3	10	30,2	30,2	47,601	0	46,571
DOK2	O60496	Docking protein 2	8	26	26	45,378	0	19,953
DOCK5	Q9H7D0	Dedicator of cytokinesis protein 5	3	1,7	1,7	215,31	0,005988	1,9992
DOCK10	Q96BY6	Dedicator of cytokinesis protein	3	1,3	1,3	249,53	0	4,4552
DNM2	P50570	Dynamin-2	19	24,4	11,1	98,063	0	43,563
DNM1L	G8JLD5	Dynamin-1-like protein	23	40,2	40,2	79,621	0	95,364
DNM1	A0A0U1RQP1	Dynamin-1	18	26,1	13,6	96,396	0	25,98
DNAJC5	Q9H3Z4	DnaJ homolog subfamily C	1	8,6	8,6	22,149	0,0038168	2,2458
DNAJB11	Q9UBS4	DnaJ homolog subfamily B	7	26,5	26,5	40,513	0	20,697
DNAJA2	O60884	DnaJ homolog subfamily A	3	7,5	7,5	45,745	0	3,9114
DNAH7	Q8WXX0	Dynein heavy chain 7, axonemal	2	0,6	0,6	461,15	0	2,9064
DMTN	Q08495	Dematin	6	16,3	16,3	45,514	0	15,666

DLST	P36957	Dihydrolipoyllysine-residue succinyltransferase component of 2-oxoglutarate dehydrogenase	5	13,5	13,5	48,755	0	19,509
DLD	P09622	Dihydrolipoyl dehydrogenase,	8	21,2	21,2	54,177	0	27,545
DKFZp686J137	Q5HYB6		31	78,9	0	27,175	0	178,59
DIAPH1	A0A2R8Y5N1	Protein diaphanous homolog 1	33	30,1	29,4	139,41	0	101,27
DHRS7	A0A087X0Z7	Dehydrogenase/reductase SDR	6	16,2	16,2	42,619	0	8,4813
DGKG	P49619	Diacylglycerol kinase gamma	2	3,9	3,9	89,123	0,0045662	2,2214
DERA	G3V158	Deoxyribose-phosphate aldolase	2	12,2	12,2	25,113	0	4,2963
DENND3	H0YAY3	DENN domain-containing protein	7	7,6	7,6	135,89	0	9,5289
DECR1	Q16698	2,4-dienoyl-CoA reductase,	8	34,9	34,9	36,067	0	33,373
DDX6	P26196	Probable ATP-dependent RNA	2	4,8	4,8	54,416	0,0045627	2,2156
DDX3X	O00571	ATP-dependent RNA helicase	2	3,6	3,6	73,243	0	4,532
DDX31	Q9H8H2	Probable ATP-dependent RNA	1	1,1	1,1	94,086	1	-2
DDX1	A0A087X2G1	ATP-dependent RNA helicase	2	5	5	73,975	0	2,9073
DDT	P30046	D-dopachrome decarboxylase	6	56,8	56,8	12,712	0	12,087
DDOST	P39656	Dolichyl-diphosphooligosaccharide--	9	21,9	21,9	50,8	0	17,228
DDB1	F5GY55	DNA damage-binding protein 1	4	3,8	3,8	121,71	0	5,5992
DCTN4	Q9UJW0	Dynactin subunit 4	1	2,8	2,8	52,337	0,00080321	2,7111
DCTN2	Q13561	Dynactin subunit 2	10	31,7	31,7	44,23	0	54,523
DCTN1	E7EX90	Dynactin subunit 1	9	9,7	9,7	139,09	0	21,338
DCN	F8VXZ8	Decorin	3	20,1	20,1	19,617	0,0015873	2,5982
DCD	P81605	Dermcidin	6	53,6	53,6	11,284	0	9,3972
DBNL	Q9UJU6	Drebrin-like protein	17	46,5	46,5	48,207	0	179,22
DBN1	Q16643	Drebrin	14	23,9	23,9	71,428	0	101,93
DARS	P14868	Aspartate--tRNA ligase,	14	33,5	33,5	57,136	0	44,616
DAPP1	J3KNB3	Dual adapter for phosphotyrosine and 3-phosphotyrosine and 3-	6	32,3	32,3	30,175	0	14,833
DAG1	Q14118	Dystroglycan	2	2,5	2,5	97,44	0	2,8186
DAD1	F5H895	Dolichyl-diphosphooligosaccharide--	1	18,5	18,5	7,2883	0	3,5489
DAB2	P98082	Disabled homolog 2	4	5,2	5,2	82,447	0	4,7941

DAAM1	Q9Y4D1	Disheveled-associated activator of morphogenesis 1	32	30	30	123,47	0	69,874
CYTH2	A0A0D9SFG6	Cytohesin-2	2	6,2	6,2	46,56	0	3,0756
CYFIP2	E7EVJ5	Cytoplasmic FMR1-interacting	23	18	1,1	142,58	0	3,9628
CYFIP1	Q7L576	Cytoplasmic FMR1-interacting	56	46,4	11,8	145,18	0	191,99
CYCS	C9JFR7	Cytochrome c	5	58,4	58,4	11,333	0	10,362
CYC1	P08574	Cytochrome c1, heme protein,	4	11,1	11,1	35,422	0	10,325
CYB5R3	P00387	NADH-cytochrome b5 reductase	19	80,1	80,1	34,234	0	232,09
CYB5R1	Q9UHQ9	NADH-cytochrome b5 reductase	9	33,8	33,8	34,094	0	15,913
CYB5B	D6RFH4	Cytochrome b5 type B	2	32,8	32,8	14,846	0	19,387
CXCR4	P61073	C-X-C chemokine receptor type 4	1	3,4	3,4	39,745	0	3,3807
CUTA	C9IZG4	Protein CutA	1	10,4	10,4	14,401	0,0015886	2,6051
CTTN	Q14247	Src substrate cortactin	24	45,5	45,5	61,585	0	148,04
CTSD	A0A1B0GW44	Cathepsin D	8	25,2	25,2	43,688	0	22,301
CTSC	H0YCY8	Dipeptidyl peptidase 1	2	9,4	9,4	27,725	0	5,3702
CTSA	X6R5C5	Carboxypeptidase	5	14,8	14,8	54,255	0	18,051
CTGF	P29279	Connective tissue growth factor	3	12,6	12,6	38,091	0	7,5754
CTDSPL	O15194	CTD small phosphatase-like	5	16,3	16,3	31,128	0	11,438
CSRP1	P21291	Cysteine and glycine-rich protein	10	59,6	59,6	20,567	0	130,27
CSNK2A1	A0A2R8Y5A0	Casein kinase II subunit alpha	4	16,4	16,4	40,717	0	4,3423
CSK	P41240	Tyrosine-protein kinase CSK	19	54,9	54,9	50,704	0	99,298
CSE1L	P55060	Exportin-2	4	4,6	4,6	110,42	0	4,0401
CS	O75390	Citrate synthase, mitochondrial	14	35,6	35,6	51,712	0	48,9
CRP	P02741	C-reactive protein	3	14,7	14,7	25,038	0	3,3277
CRLF3	Q8IUI8	Cytokine receptor-like factor 3	4	14,7	14,7	49,765	0	10,5
CRKL	P46109	Crk-like protein	13	51,5	51,5	33,777	0	52,74
CPT1A	P50416	Carnitine O-palmitoyltransferase	11	15,1	15,1	88,367	0	20,707
CPPED1	Q9BRF8	Serine/threonine-protein	2	4,5	4,5	35,548	0	3,589
CPNE3	O75131	Copine-3	6	14,3	12,7	60,13	0	16,813
CPNE2	Q96FN4	Copine-2	2	4,4	2,7	61,189	0,0079308	1,693
CPNE1	F2Z2V0	Copine-1	7	10,7	10,7	58,634	0	13,73
COX7A2	D6RIE3	Cytochrome c oxidase subunit	2	25,3	25,3	10,264	0	3,6841
COX6C	P09669	Cytochrome c oxidase subunit 6C	1	17,3	17,3	8,7813	0,0045593	2,2122

COX6B1	P14854	Cytochrome c oxidase subunit	1	12,8	12,8	10,192	0,003125	2,4391
COX6A1	P12074	Cytochrome c oxidase subunit	2	43,1	43,1	12,155	0	2,9562
COX5B	P10606	Cytochrome c oxidase subunit 5B,	4	29,5	29,5	13,696	0	8,5684
COX5A	P20674	Cytochrome c oxidase subunit 5A,	6	38	38	16,762	0	23,265
COX4I1	P13073	Cytochrome c oxidase subunit 4 isoform 1, mitochondrial	7	34,9	34,9	19,576	0	14,978
COTL1	Q14019	Coactosin-like protein	14	78,9	78,9	15,945	0	68,824
CORO1C	Q9ULV4	Coronin-1C	28	51,7	49,8	53,248	0	214,6
CORO1B	Q9BR76	Coronin-1B	11	19	17,2	54,234	0	35,812
CORO1A	P31146	Coronin-1A	21	43,2	43,2	51,026	0	104
COPZ1	F8VXB1	Coatomer subunit zeta-1	1	16,3	16,3	10,628	0	4,1032
COPS8	Q99627	COP9 signalosome complex	2	13,9	13,9	23,225	0	5,2307
COPS7A	F5H4U8	COP9 signalosome complex	2	20,8	20,8	13,663	0,0052632	2,0963
COPS6	E7EM64	COP9 signalosome complex	1	4,9	4,9	36,064	0,0059925	1,9994
COPS5	Q92905	COP9 signalosome complex	3	10,5	10,5	37,578	0	4,0384
COPS4	D6RAX7	COP9 signalosome complex	3	10	10	47,726	0	3,3459
COPS2	B4DIH5	COP9 signalosome complex	5	15,8	15,8	44,228	0	9,2236
COPG1	Q9Y678	Coatomer subunit gamma-1	3	3,9	3,9	97,717	0	5,3082
COPE	O14579	Coatomer subunit epsilon	2	7,8	7,8	34,482	0	6,037
COPB2	P35606	Coatomer subunit beta	13	17,3	17,3	102,49	0	20,419
COPB1	P53618	Coatomer subunit beta	5	10	10	107,14	0	15,182
COPA	P53621	Coatomer subunit alpha	16	16,7	16,7	138,34	0	33,46
COMT	P21964	Catechol O-methyltransferase	1	8,1	8,1	30,037	0	3,5457
COASY	Q13057	Bifunctional coenzyme A	2	4,3	4,3	62,328	0	3,4939
CNST	Q6PJW8	Consortin	4	9,2	9,2	79,596	0	6,2385
CNP	P09543	2,3-cyclic-nucleotide 3-	7	19,2	19,2	47,578	0	21,22
CNN2	B4DDF4	Calponin	11	40,9	40,9	32,616	0	73,472
CNDP2	Q96KP4	Cytosolic non-specific dipeptidase	13	43,2	43,2	52,878	0	32,903
CMTM6	Q9NX76	CKLF-like MARVEL transmembrane domain-	3	21,9	21,9	20,419	0	13,369
CMTM5	C9JAI6	CKLF-like MARVEL transmembrane domain-	2	27,1	27,1	13,238	0	20,437
CMPK1	Q5T0D2	UMP-CMP kinase	4	25,4	25,4	18,979	0	5,8968

CMIP	A0A087WU05	C-Maf-inducing protein	10	19,8	19,8	65,284	0	20,512
CLU	P10909	Clusterin	13	33,9	33,9	52,494	0	69,803
CLTC	A0A087WVQ6	Clathrin heavy chain	49	36	36	192,06	0	295,94
CLTA	P09496	Clathrin light chain A	5	14,1	14,1	27,076	0	6,0508
CLINT1	Q14677	Clathrin interactor 1	7	11,7	11,7	68,259	0	13,428
CLIC5	A0A2R8Y4M1	Chloride intracellular channel	1	8,5	8,5	22,284	0	2,7318
CLIC4	Q9Y696	Chloride intracellular channel	12	68	68	28,772	0	74,618
CLIC1	O00299	Chloride intracellular channel	20	88,4	88,4	26,922	0	146,21
CLEC2L	P0C7M8	C-type lectin domain family 2	2	12,6	12,6	23,926	0	6,1432
CLEC1B	Q9P126	C-type lectin domain family 1	7	27,9	27,9	26,595	0	15,729
CLDN5	D3DX19	Claudin	3	12,5	12,5	31,626	0	7,1813
CLDN3	O15551	Claudin-3	1	5,5	5,5	23,318	0,0072674	1,756
CLCN3	A0A087WU31	Chloride channel protein	3	2,8	2,8	87,947	0,0038432	2,3057
CKAP5	Q14008	Cytoskeleton-associated protein	21	13,4	13,4	225,49	0	45,725
CISD2	Q8N5K1	CDGSH iron-sulfur domain-	4	36,3	36,3	15,278	0	11,392
CIB1	Q99828	Calcium and integrin-binding	1	8,9	8,9	21,703	0,00080128	2,6663
CHP1	Q99653	Calcineurin B homologous protein	4	26,7	26,7	22,456	0	9,3171
CHMP6	Q96FZ7	Charged multivesicular body	5	29,4	29,4	23,485	0	18,384
CHMP4B	Q9H444	Charged multivesicular body	2	11,2	11,2	24,95	0	4,4987
CFL1	P23528	Cofilin-1	19	79,5	75,3	18,502	0	199,03
CFAP53	Q96M91	Cilia- and flagella-associated	1	2,9	2,9	61,834	0,0023697	2,5291
CFAP44	H0Y896	Cilia- and flagella-associated	1	2,9	2,9	49,55	0,0086145	1,6572
CERS2	Q5SZE1	Ceramide synthase 2	3	8,6	8,6	36,373	0	6,255
CDS2	O95674	Phosphatidate	4	15,1	15,1	51,417	0	16,925
CDK4	F8VXD2	Cyclin-dependent kinase 12	1	6,1	6,1	14,9	0	5,984
CDC42BPB	Q9Y5S2	Serine/threonine-protein kinase	6	4	4	194,31	0	7,7281
CDC42	P60953	Cell division control protein 42	8	42,4	36,6	21,258	0	35,076
CDC37	Q16543	Hsp90 co-chaperone Cdc37	4	13,2	13,2	44,468	0	7,2655
CD99	P14209	CD99 antigen	2	13	13	18,848	0	7,4812
CD9	A6NNI4	Tetraspanin	11	47,8	47,8	17,764	0	323,31
CD84	Q9UIB8	SLAM family member 5	8	33,3	33,3	38,782	0	26,696
CD82	P27701	CD82 antigen	3	16,5	16,5	29,625	0	7,1677
CD69	Q07108	Early activation antigen CD69	2	11,1	11,1	22,559	0	4,2506

CD68	J3KRX0	Macrosialin	1	26,4	26,4	13,18	0	14,715
CD63	F8VNT9	Tetraspanin	1	7,5	7,5	14,265	0	12,362
CD59	E9PNW4	CD59 glycoprotein	3	29,6	29,6	11,985	0	6,6678
CD55	H3BLV0	Complement decay-accelerating	7	22,4	22,4	35,695	0	17,644
CD47	A0A2R8Y484	Leukocyte surface antigen CD47	3	15,3	15,3	20,313	0	10,713
CD46	P15529	Membrane cofactor protein	1	3,8	3,8	43,747	0	5,8027
CD40	A0A087X1D0	Tumor necrosis factor receptor superfamily member 5	2	11,9	11,9	17,035	0,0074239	1,8826
CD36	E7EU05	Platelet glycoprotein 4	16	35	35	52,062	0	214,45
CD226	Q15762	CD226 antigen	5	20,8	20,8	38,614	0	59,419
CD151	K4DIA7	Tetraspanin	5	19,1	4,3	25,588	0	10,325
CD109	Q6YHK3	CD109 antigen	13	12,6	12,6	161,69	0	38,255
CCT8	P50990	T-complex protein 1 subunit	29	60	60	59,62	0	69,236
CCT7	Q99832	T-complex protein 1 subunit eta	13	24,3	24,3	59,366	0	42,119
CCT6A	P40227	T-complex protein 1 subunit zeta	16	36,2	36,2	58,024	0	55,276
CCT5	P48643	T-complex protein 1 subunit	19	38,1	38,1	59,67	0	47,058
CCT4	P50991	T-complex protein 1 subunit delta	22	43,2	43,2	57,924	0	66,206
CCT3	P49368	T-complex protein 1 subunit	21	43,3	43,3	60,533	0	94,032
CCT2	P78371	T-complex protein 1 subunit beta	24	54,4	54,4	57,488	0	125,52
CCS	J3KNF4	Copper chaperone for superoxide	6	28,6	28,6	27,086	0	18,387
CCNY	Q8ND76	Cyclin-Y	2	9,4	9,4	39,336	0	8,3904
CCL5	P13501	C-C motif chemokine 5	8	48,4	48,4	9,9896	0	10,1
CBR1	P16152	Carbonyl reductase [NADPH] 1	7	34,7	34,7	30,375	0	29,341
CAT	P04040	Catalase	24	49,5	49,5	59,755	0	130,58
CASS4	Q9NQ75	Cas scaffolding protein family	5	7,5	7,5	87,143	0	21,592
CASQ1	P31415	Calsequestrin-1	1	3,3	3,3	45,16	1	-2
CASP6	P55212	Caspase-6	3	13,3	13,3	33,31	0	5,2637
CASP3	P42574	Caspase-3	6	29,6	29,6	31,608	0	9,4465
CAPZB	B1AK87		19	71,5	6,2	29,295	0	188,2
CAPZB	P47756	F-actin-capping protein subunit	18	66,1	4,7	31,35	0	22,364
CAPZA2	P47755	F-actin-capping protein subunit	14	67,8	58,4	32,949	0	163,89
CAPZA1	P52907	F-actin-capping protein subunit	16	74,1	64,7	32,922	0	193,69
CAPNS1	A0A075B7C0	Calpain small subunit 1	11	73,6	73,6	21,911	0	76,872

CAPN1	P07384	Calpain-1 catalytic subunit	36	51	51	81,889	0	188,74
CAP1	Q01518	Adenylyl cyclase-associated	37	77,3	77,3	51,901	0	323,31
CANX	P27824	Calnexin	21	36,1	36,1	67,567	0	82,539
CAND1	Q86VP6	Cullin-associated NEDD8-	15	15,9	15,9	136,37	0	32,463
CAMSAP2	Q08AD1	Calmodulin-regulated spectrin-	1	0,5	0,5	168,09	1	-2
CAMK1	Q14012	Calcium/calmodulin-dependent protein kinase type 1	9	31,1	31,1	41,337	0	15,323
CALU	O43852	Calumenin	6	25,4	25,4	37,106	0	19,008
CALR	P27797	Calreticulin	17	51,8	51,8	48,141	0	159,35
CALML5	Q9NZT1	Calmodulin-like protein 5	4	30,8	30,8	15,892	0	5,8384
CALM2	P0DP25		11	85,9	85,9	16,837	0	213,15
CALD1	E7EX44	Caldesmon	23	41,1	3,1	64,168	0,009319	1,6509
CALD1	E9PGZ1		24	42	2,4	61,705	0	96,344
CACYBP	Q9HB71	Calcyclin-binding protein	4	23,7	23,7	26,21	0	6,6741
CAB39	A0A087X0K1	Calcium-binding protein 39	7	19,5	19,5	39,452	0	10,805
CA2	P00918	Carbonic anhydrase 2	15	62,7	62,7	29,246	0	63,969
CA13	Q8N1Q1	Carbonic anhydrase 13	3	17,9	17,9	29,443	0	19,173
CA1	E5RJF6	Carbonic anhydrase 1	2	24,1	24,1	9,4069	0,0073855	1,8529
C4BPA	P04003	C4b-binding protein alpha chain	4	8,7	8,7	67,033	0	6,7746
C3	P01024	Complement C3	6	5,5	5,5	187,15	0	18,159
C2orf88	Q9BSF0	Small membrane A-kinase anchor	2	32,6	32,6	10,97	0	3,1832
C21orf33	P0DPI2		5	34,3	34,3	28,17	0	29,916
C20orf27	Q9GZN8	UPF0687 protein C20orf27	1	8	8	19,291	0,0086455	1,6765
C1orf95	F8WD64	Protein stum homolog	1	9,2	9,2	14,968	0,0092924	1,6273
C1orf198	Q9H425	Uncharacterized protein	2	7,6	7,6	36,346	0,0045489	2,1987
C10orf54	Q9H7M9	Platelet receptor Gi24	6	23,8	23,8	33,908	0	12,327
BTN3A2	S4R3N0	Butyrophilin subfamily 3 member	1	10,4	10,4	12,932	0	3,4825
BTK	Q06187	Tyrosine-protein kinase BTK	21	37,2	37,2	76,28	0	64,026
BSG	P35613	Basigin	7	23,1	23,1	42,2	0	42,091
BRK1	Q8WUW1	Protein BRICK1	9	77,3	77,3	8,7448	0	25,391
BNIP2	H7C096	BCL2/adenovirus E1B 19 kDa protein-interacting protein 2	2	12,3	12,3	24,225	0	4,7257
BLVRB	M0R192	Flavin reductase (NADPH)	1	5,5	5,5	17,16	0	3,5051

BLVRA	P53004	Biliverdin reductase A	4	17,6	17,6	33,428	0	10,551
BLMH	K7ESE8	Bleomycin hydrolase	5	26	26	26,738	0	11,16
BIN2	A0A087X188	Bridging integrator 2	20	39,2	39,2	65,023	0	132,67
BID	P55957	BH3-interacting domain death	5	32,8	32,8	21,994	0	20,905
BGN	P21810	Biglycan	6	19,8	19,8	41,654	0	11,21
BDNF	P23560	Brain-derived neurotrophic factor	1	4,9	4,9	27,818	0	3,1702
BCAP31	P51572	B-cell receptor-associated protein	9	33,7	33,7	27,991	0	19,619
BAX	Q07812	Apoptosis regulator BAX	2	12,5	12,5	21,184	0	4,5589
BANF1	O75531	Barrier-to-autointegration factor	2	42,7	42,7	10,058	0	4,8218
BAG6	F6S6P2	Large proline-rich protein BAG6	2	4,5	4,5	54,67	0	4,0883
B2M	H0YLF3		3	46,5	18,3	8,4984	0	5,0771
B2M	P61769	Beta-2-microglobulin	3	35,3	18,5	13,714	0	147,67
ATPIF1	Q9UII2	ATPase inhibitor, mitochondrial	2	10,4	10,4	12,249	0	3,5574
ATP8A1	Q9Y2Q0	Phospholipid-transporting ATPase	15	15,5	15,5	131,37	0	32,129
ATP6V1G1	O75348	V-type proton ATPase subunit G 1	3	32,2	32,2	13,757	0	33,062
ATP6V1E1	P36543	V-type proton ATPase subunit E 1	2	11,9	11,9	26,145	0	12,261
ATP6V1B2	P21281	V-type proton ATPase subunit B,	8	22,3	22,3	56,5	0	15,573
ATP6V1A	P38606	V-type proton ATPase catalytic	16	33,5	33,5	68,303	0	56,105
ATP6V0D1	J3QL14	V-type proton ATPase subunit d 1	2	10,3	10,3	26,705	0	3,6933
ATP6V0A1	Q93050	V-type proton ATPase 116 kDa	6	7,6	7,6	96,412	0	12,588
ATP5O	P48047	ATP synthase subunit O,	9	55,4	55,4	23,277	0	36,077
ATP5L	E9PN17	ATP synthase subunit g,	4	63,2	63,2	8,4518	0	11,265
ATP5J2-PTCD1	C9JJT5		3	61,1	16,7	5,9149	0	9,4643
ATP5H	O75947	ATP synthase subunit d,	5	36	36	18,491	0	8,5996
ATP5F1	Q5QNZ2	ATP synthase F(0) complex subunit B1, mitochondrial	6	28,7	28,7	22,275	0	39,643
ATP5D	P30049	ATP synthase subunit delta,	2	13,7	13,7	17,49	0	9,8222
ATP5C1	P36542	ATP synthase subunit gamma,	7	27,2	27,2	32,996	0	17,694
ATP5B	P06576	ATP synthase subunit beta,	23	65,4	65,4	56,559	0	256,81
ATP5A1	P25705	ATP synthase subunit alpha,	29	56,2	56,2	59,75	0	205,81
ATP2C1	B4E2Q0	Calcium-transporting ATPase	2	3,3	3,3	104,71	0,0073153	1,7901
ATP2A3	Q93084	Sarcoplasmic/endoplasmic reticulum calcium ATPase 3	35	39,9	32,4	113,98	0	311,64

ATP2A2	P16615	Sarcoplasmic/endoplasmic reticulum calcium ATPase 2	30	31,2	23,6	114,76	0	68,699
ATP1B3	P54709	Sodium/potassium-transporting ATPase subunit beta-3	5	24,7	24,7	31,512	0	11,324
ATP1A1	P05023	Sodium/potassium-transporting ATPase subunit alpha-1	22	24,4	20,9	112,89	0	90,237
ATL3	Q6DD88	Atlastin-3	4	9,6	9,6	60,541	0	13,365
ATIC	P31939	Bifunctional purine biosynthesis	9	21,3	21,3	64,615	0	29,344
ATG4A	G5E979		1	3,5	3,5	26,515	1	-2
ATE1	B4E107	Arginyl-tRNA--protein transferase	2	6,9	6,9	47,834	0	4,6823
ATAD3A	H0Y2W2	ATPase family AAA domain-	1	1,4	1,4	64,243	0,0093257	1,6533
ASNA1	O43681	ATPase ASNA1	4	15,2	15,2	38,792	0	7,2611
ASAP2	O43150	Arf-GAP with SH3 domain, ANK repeat and PH domain-containing	5	7,1	5,2	111,65	0	30,327
ASAP1	A0A0A0MRE5	Arf-GAP with SH3 domain, ANK repeat and PH domain-containing	13	16	14,3	124,77	0	25,1
ART4	H7C2G2	NAD(P)(+)--arginine ADP-	1	2,6	2,6	31,511	1	-2
ARRB1	H0YET9		5	27,2	9,2	21,805	0,0015974	2,6219
ARRB1	P49407	Beta-arrestin-1	9	24,6	16,3	47,065	0	43,042
ARPC5L	Q9BPX5	Actin-related protein 2/3 complex subunit 5-like protein	3	21,6	16,3	16,941	0	5,0679
ARPC5	O15511	Actin-related protein 2/3	9	72,2	72,2	16,32	0	73,167
ARPC4-TTLL3	F8WCF6	Actin-related protein 2/3	10	66,9	66,9	21,058	0	85,135
ARPC3	O15145	Actin-related protein 2/3	10	66,9	66,9	20,546	0	84,311
ARPC2	O15144	Actin-related protein 2/3	29	81	81	34,333	0	323,31
ARPC1B	O15143	Actin-related protein 2/3	26	69,4	16,4	40,949	0	300,48
ARPC1A	Q92747	Actin-related protein 2/3	5	21,1	7	41,569	0	4,3593
ARL8B	Q9NVJ2	ADP-ribosylation factor-like	6	43	29	21,539	0	14,589
ARL8A	Q96BM9	ADP-ribosylation factor-like	4	34,4	8,1	21,416	0	2,7756
ARL6IP5	O75915	PRA1 family protein 3	5	28,7	28,7	21,614	0	27,966
ARL15	A0A0B4J222	ADP-ribosylation factor-like	1	7,6	7,6	17,444	0	3,0947
ARL1	F8VYN9	ADP-ribosylation factor-like	2	12,9	12,9	21,778	0	5,4477
ARHGEF7	E7EUY6	Rho guanine nucleotide exchange	1	4,5	4,5	50,493	0	3,6047

ARHGDIB	P52566	Rho GDP-dissociation inhibitor 2	16	78,1	78,1	22,988	0	201,29
ARHGDIA	P52565	Rho GDP-dissociation inhibitor 1	8	53,4	53,4	23,207	0	72,181
ARHGAP6	H7BYE6	Rho GTPase-activating protein 6	10	24	24	67,311	0	18,911
ARHGAP18	Q8N392	Rho GTPase-activating protein 18	23	32,9	32,9	74,976	0	56,189
ARHGAP1	Q07960	Rho GTPase-activating protein 1	18	56,5	56,5	50,435	0	98,797
ARF6	P62330	ADP-ribosylation factor 6	2	12,6	12,6	20,082	0	3,7533
ARF5	C9J1Z8	ADP-ribosylation factor 5	6	57,3	16	17,107	0	8,876
ARF4	P18085	ADP-ribosylation factor 4	8	56,1	27,2	20,511	0	29,921
ARF1	P84077	ADP-ribosylation factor 1	10	64,1	35,4	20,697	0	91,91
ARCN1	P48444	Coatomer subunit delta	5	10,8	10,8	57,21	0	6,1773
ARAP1	Q96P48	Arf-GAP with Rho-GAP domain, ANK repeat and PH domain-	12	12	12	162,19	0	24,784
APRT	P07741	Adenine	7	52,2	52,2	19,608	0	42,167
APPL2	Q8NEU8	DCC-interacting protein 13-beta	8	16,3	16,3	74,493	0	24,355
APP	E9PG40	Amyloid beta A4 protein	19	30,4	29,3	80,832	0	106,3
APOE	P02649	Apolipoprotein E	12	48,6	48,6	36,154	0	82,018
APOD	P05090	Apolipoprotein D	2	9,5	9,5	21,275	0,0038402	2,2998
APOC3	B0YIW2	Apolipoprotein C-III	2	23,1	23,1	12,815	0	19,902
APOB	P04114	Apolipoprotein B-100	129	32,8	32,8	515,6	0	323,31
APOA2	V9GYE3	Apolipoprotein A-II	2	40,4	40,4	5,8767	0	3,0653
APOA1BP	Q8NCW5	NAD(P)H-hydrate epimerase	2	10,4	10,4	31,674	0	3,0697
APOA1	P02647	Apolipoprotein A-I	16	54,7	54,7	30,777	0	53,812
APMAP	H0Y512	Adipocyte plasma membrane-	2	5,6	5,6	45,294	0	5,5304
APLP2	Q06481	Amyloid-like protein 2	9	13,8	12,7	86,955	0	19,355
APEH	C9JIF9	Acylamino-acid-releasing enzyme	4	6,5	6,5	81,673	0	8,0324
AP3D1	O14617	AP-3 complex subunit delta-1	3	3	3	130,16	0	5,1053
AP3B1	O00203	AP-3 complex subunit beta-1	3	2,8	2,8	121,32	0	5,2586
AP2M1	A0A087WY71	AP-2 complex subunit mu	1	4,6	4,6	49,526	0	3,9015
AP2B1	P63010	AP-2 complex subunit beta	11	11,8	2,7	104,55	0	13,064
AP2A1	O95782	AP-2 complex subunit alpha-1	4	5,4	5,4	107,54	0	4,4053
AP1S1	A0A2R8YGH5	AP-1 complex subunit sigma-1A	3	15,3	10,2	18,602	0,0074184	1,8736
AP1M1	Q9BXS5	AP-1 complex subunit mu-1	12	40,4	40,4	48,586	0	25,857
AP1G1	O43747	AP-1 complex subunit gamma-1	11	14,1	14,1	91,35	0	27,202

AP1B1	Q10567	AP-1 complex subunit beta-1	10	11,8	2,7	104,64	0	5,0507
ANXA7	P20073	Annexin A7	14	33,6	32,2	52,739	0	50,46
ANXA5	P08758	Annexin A5	11	30,9	30,9	35,936	0	28,777
ANXA4	P09525	Annexin A4	9	35,7	35,7	35,882	0	25,542
ANXA3	P12429	Annexin A3	9	34,4	34,4	36,375	0	18,64
ANXA11	P50995	Annexin A11	13	31,3	29,9	54,389	0	31,504
ANO6	Q4KMQ2	Anoctamin-6	24	31,3	31,3	106,16	0	95,682
ANKS1A	Q92625	Ankyrin repeat and SAM domain-containing protein 1A	1	1,1	1,1	123,11	0	3,587
ANK1	P16157	Ankyrin-1	8	6,3	6,3	206,26	0	10,292
ANGPT1	Q15389	Angiopoietin-1	3	7	7	57,512	0	12,729
AMPD2	H0Y360	AMP deaminase 2	12	14,8	14,8	98,401	0	17,784
ALOX12	P18054	Arachidonate 12-lipoxygenase,	30	51,4	51,4	75,693	0	150,25
ALDOC	P09972	Fructose-bisphosphate aldolase C	7	20,9	7,1	39,455	0	57,886
ALDOA	P04075	Fructose-bisphosphate aldolase A	29	90,9	77,2	39,42	0	323,31
ALDH9A1	P49189	4-trimethylaminobutyraldehyde	2	4,9	4,9	53,801	0	3,6196
ALDH1A1	P00352	Retinal dehydrogenase 1	2	5	5	54,861	0	3,672
ALDH16A1	F5H4B6	Aldehyde dehydrogenase family	3	8	8	67,152	0	5,6098
ALB	CON__P02768-	Serum albumin	51	77,8	72,7	69,366	0	323,31
ALAD	P13716	Delta-aminolevulinic acid	11	41,5	41,5	36,294	0	38,568
AKT2	P31751	RAC-beta serine/threonine-	6	13,9	9,6	55,768	0	7,2151
AKR7A2	H3BLU7	Aflatoxin B1 aldehyde reductase	9	34,7	34,7	34,684	0	38,003
AKR1A1	P14550	Alcohol dehydrogenase	4	14,2	14,2	36,573	0	9,1067
AK3	Q9UIJ7	GTP:AMP phosphotransferase	5	29,1	29,1	25,565	0	13,369
AK2	F8W1A4	Adenylate kinase 2,	6	37,1	37,1	25,63	0	8,2694
AK1	Q5T9B7	Adenylate kinase isoenzyme 1	5	33,3	33,3	23,411	0	7,9777
AIFM1	O95831	Apoptosis-inducing factor 1,	3	7,2	7,2	66,9	0	4,5061
AHSG	C9JV77	Alpha-2-HS-glycoprotein	1	3,8	3,8	39,411	0	4,3256
AHCYL2	H0Y8B3	Adenosylhomocysteinase	7	15,3	15,3	58,004	0	10,046
AHCY	P23526	Adenosylhomocysteinase	5	13,2	13,2	47,716	0	7,5689
ADSL	A0A096LNY6	Adenylosuccinate lyase	2	8,3	8,3	40,826	0,0030888	2,3437
ADRBK1	P25098	Beta-adrenergic receptor kinase 1	2	3,6	3,6	79,573	0	3,3533
ADH5	P11766	Alcohol dehydrogenase class-3	9	31,8	31,8	39,724	0	29,388

ADD3	Q9UEY8	Gamma-adducin	3	5	5	79,154	0	8,8982
ADD1	A0A0A0MSR2	Alpha-adducin	3	11,2	11,2	43,99	0	10,437
ADCY6	O43306	Adenylate cyclase type 6	13	14	14	130,61	0	45,801
ADAM10	O14672	Disintegrin and metalloproteinase domain-	18	30,7	30,7	84,141	0	188,86
ACTR3C	C9IZN3		4	18	3,6	24,32	1	-2
ACTR3	P61158	Actin-related protein 3	26	76,8	72,5	47,371	0	323,31
ACTR2	P61160	Actin-related protein 2	21	62,2	62,2	44,76	0	279,03
ACTR1B	P42025	Beta-centractin	8	28,5	4	42,293	0	3,3078
ACTR1A	P61163	Alpha-centractin	10	39,1	14,6	42,613	0	52,161
ACTR10	V9GYX7	Actin-related protein 10	1	3	3	33,201	0,0015823	2,5475
ACTN4	O43707	Alpha-actinin-4	60	72,3	50,4	104,85	0	278,87
ACTN1	H9KV75		68	82	2,1	94,825	0	3,48
ACTN1	P12814	Alpha-actinin-1	78	85,5	10,2	103,06	0	323,31
ACTG1	K7EM38		14	91,7	7,5	14,522	0	2,7591
ACTG1	P63261	Actin, cytoplasmic 2	38	90,4	0	41,792	0	323,31
ACTBL2	Q562R1	Beta-actin-like protein 2	11	25,8	10,4	42,003	0,0015936	2,6164
ACTB	P60709	Actin, cytoplasmic 1	38	90,4	0	41,736	0	127,5
ACTA1	P68133	Actin, alpha skeletal muscle	25	43,2	15,1	42,051	0	106,1
ACSL4	O60488	Long-chain-fatty-acid--CoA ligase	3	5,6	5,6	79,187	0	4,9736
ACP1	P24666	Low molecular weight phosphotyrosine protein	5	28,5	28,5	18,042	0	12,627
ACOX1	Q15067	Peroxisomal acyl-coenzyme A	4	8,6	8,6	74,423	0	8,6751
ACOT9	Q9Y305	Acyl-coenzyme A thioesterase 9,	2	3,6	3,6	49,901	0,0038491	2,3305
ACOT7	K7EKP8	Cytosolic acyl coenzyme A	2	7,9	7,9	30,792	0,0074129	1,8725
ACO2	A2A274	Aconitate hydratase,	8	13,9	13,9	87,819	0	18,955
ACO1	P21399	Cytoplasmic aconitate hydratase	9	12,1	12,1	98,398	0	19,471
ACLY	P53396	ATP-citrate synthase	19	24,1	24,1	120,84	0	49,329
ACAT2	Q9BWD1	Acetyl-CoA acetyltransferase,	4	19,9	19,9	41,35	0	14,386
ACAP2	H7C3K3	Arf-GAP with coiled-coil, ANK repeat and PH domain-containing	1	5,7	5,7	25,607	0,0030864	2,3398
ACADVL	P49748	Very long-chain specific acyl-CoA dehydrogenase, mitochondrial	12	23,5	23,5	70,389	0	27,951

ACADM	B7Z9I1	Medium-chain specific acyl-CoA dehydrogenase, mitochondrial	4	12,2	12,2	42,426	0	8,2979
ABRACL	Q9P1F3	Costars family protein ABRACL	2	30,9	30,9	9,0564	0	22,714
ABI2	E9PEZ7	Abl interactor 2	10	16,8	8,5	49,25	0	4,0985
ABI1	B6VEX4	Abl interactor 1	16	40,5	30,8	43,294	0	87,641
ABHD16A	A0A0G2JJD3	Abhydrolase domain-containing	12	18,3	18,3	67,518	0	24,041
ABHD14B	Q96IU4	Alpha/beta hydrolase domain-containing protein 14B	4	33,8	33,8	22,345	0	7,8338
ABHD11	Q8NFV4	Alpha/beta hydrolase domain-	3	16,5	16,5	34,69	0	9,4716
ABHD10	Q9NUJ1	Mycophenolic acid acyl-glucuronide esterase,	2	9,5	9,5	33,932	0,0031201	2,436
ABCC4	O15439	Multidrug resistance-associated	9	7,4	7,4	149,52	0	14,515
ABCC1	A0A0A0MS99	Multidrug resistance-associated	2	1,6	1,6	134,99	0,0023641	2,5225
ABCB6	Q9NP58	ATP-binding cassette sub-family B member 6, mitochondrial	6	9	9	93,884	0	10,493
ABCB11	O95342	Bile salt export pump	1	1,1	1,1	146,41	1	-2
AARS	P49588	Alanine--tRNA ligase, cytoplasmic	6	8,6	8,6	106,81	0	21,953
A2M	P01023	Alpha-2-macroglobulin	2	2,2	2,2	163,29	0	3,7707
SEPT11	D6RGI3	Septin-11	13	35,1	15,8	49,005	0	10,855
SEPT7	E7ES33	Septin-7	17	48,9	48,9	48,715	0	93,982
SEPT6	B1AMS2	Septin-6	15	44,1	25,1	49,303	0	98,66
SEPT5	C9JM82	Septin-5	5	18,9	18,9	34,672	0	23,142
SEPT2	B5MCX3	Septin-2	11	46,7	46,7	36,94	0	48,416
	A0A0G2JR96		39	45,7	1,4	100,3	0	3,5765
	A0A140T9L8		12	59,9	21,1	24,903	0	95,048
	A0A1B0GUL3		1	1	1	95,079	1	-2
	A0A1W2PNV4		22	36,4	0	75,872	0	9,6428
synthase	A0A2R8Y793		29	91,3	3,2	34,141	0,0072569	1,7446

Table S3. List of modulated proteins in mEVs of CRC patients vs. HS

REDUCED

ABCC4	Multidrug resistance-associated protein 4
ACO1	Cytoplasmic aconitate hydratase
ACTB	Actin, cytoplasmic 1
ACTN4	Alpha-actinin-4
ADD1	Alpha-adducin
AK1	Adenylate kinase isoenzyme 1
ALDOC	Fructose-bisphosphate aldolase C
AP1B1	AP-1 complex subunit beta-1
APOA1	Apolipoprotein A-I;Proapolipoprotein A-I;Truncated apolipoprotein A-I
APOD	Apolipoprotein D
APP	Amyloid beta A4 protein;N-APP;Soluble APP-alpha;Soluble APP-beta;C99;Beta-amyloid protein 42;Beta-amyloid protein 40;C83;P3(42);P3(40);C80;Gamma-secretase C-terminal fragment 59;Gamma-secretase C-terminal fragment 57;Gamma-secretase C-terminal fragment 50;C31
APPL2	DCC-interacting protein 13-beta
ARCN1	Coatomer subunit delta
ARF1	ADP-ribosylation factor 1
ARHGAP1	Rho GTPase-activating protein 1
ATL3	Atlastin-3
BIN2	Bridging integrator 2
CA13	Carbonic anhydrase 13
CA2	Carbonic anhydrase 2
CALR	Calreticulin
CALU	Calumenin
CAT	Catalase
CBR1	Carbonyl reductase [NADPH] 1
CD47	Leukocyte surface antigen CD47
CDC42	Cell division control protein 42 homolog
CISD2	CDGSH iron-sulfur domain-containing protein 2
CKAP5	Cytoskeleton-associated protein 5

CLIC1	Chloride intracellular channel protein 1
COPB1	Coatomer subunit beta
COTL1	Coactosin-like protein
CSK	Tyrosine-protein kinase CSK
CTTN	Src substrate cortactin
CYFIP1	Cytoplasmic FMR1-interacting protein 1
DENND3	DENN domain-containing protein 3
DHRS7	Dehydrogenase/reductase SDR family member 7
DNAJA2	DnaJ homolog subfamily A member 2
DNM1	Dynamin-1
EHD1	EH domain-containing protein 1
ENO1	Alpha-enolase;Enolase
ERAP1	Endoplasmic reticulum aminopeptidase 1
F5	Coagulation factor V;Coagulation factor V heavy chain;Coagulation factor V light chain
FAM26E	Protein FAM26E
FERMT3	Fermitin family homolog 3
FGA	Fibrinogen alpha chain;Fibrinopeptide A;Fibrinogen alpha chain
FGB	Fibrinogen beta chain;Fibrinopeptide B;Fibrinogen beta chain
FGG	Fibrinogen gamma chain
FHL1	Four and a half LIM domains protein 1
FHOD1	FH1/FH2 domain-containing protein 1
FLNC	Filamin-C
GANAB	Neutral alpha-glucosidase AB
GP1BB	Platelet glycoprotein Ib beta chain
GP5	Platelet glycoprotein V
GPI	Glucose-6-phosphate isomerase
GRB2	Growth factor receptor-bound protein 2
GRHPR	Glyoxylate reductase/hydroxypyruvate reductase
HBA1	Hemoglobin subunit alpha
HBB	Hemoglobin subunit beta;LVV-hemorphin-7;Spinorphin
HSPA5	78 kDa glucose-regulated protein
IGHG1	Ig gamma-1 chain C region
INF2	Inverted formin-2
LASP1	LIM and SH3 domain protein 1
LGALSL	Galectin-related protein;Galectin
LMAN1	Protein ERGIC-53

LTBP1	Latent-transforming growth factor beta-binding protein 1
LYPLA1	Acyl-protein thioesterase 1
MGLL	Monoglyceride lipase
MMRN1	Multimerin-1;Platelet glycoprotein Ia*;155 kDa platelet multimerin
MOB1B	MOB kinase activator 1B
MTMR12	Myotubularin-related protein 12
MTPN	Myotrophin
MYL6	Myosin light polypeptide 6
MYLK	Myosin light chain kinase, smooth muscle;Myosin light chain kinase, smooth muscle, deglutamylated form
NAP1L4	Nucleosome assembly protein 1-like 4
NCKAP1	Nck-associated protein 1
NCKAP1L	Nck-associated protein 1-like
OTUB1	Ubiquitin thioesterase OTUB1
P4HB	Protein disulfide-isomerase
PACS1	Phosphofurin acidic cluster sorting protein 1
PARK7	Protein deglycase DJ-1
PDE5A	cGMP-specific 3,5-cyclic phosphodiesterase
PDIA3	Protein disulfide-isomerase A3
PDIA6	Protein disulfide-isomerase A6
PDLIM1	PDZ and LIM domain protein 1
PLEC	Plectin
PLEK	Pleckstrin
PPBP	Platelet basic protein;Connective tissue-activating peptide III;TC-2;Connective tissue-activating peptide III(1-81);Beta-thromboglobulin;Neutrophil-activating peptide 2(74);Neutrophil-activating peptide 2(73);Neutrophil-activating peptide 2;TC-1;Neutrophil-activating peptide 2(1-66);Neutrophil-activating peptide 2(1-63)
PPIA	Peptidyl-prolyl cis-trans isomerase A;Peptidyl-prolyl cis-trans isomerase A, N-terminally processed;Peptidyl-prolyl cis-trans isomerase
PPIB	Peptidyl-prolyl cis-trans isomerase B
PPP2CA	Serine/threonine-protein phosphatase 2A catalytic subunit alpha isoform;Serine/threonine-protein phosphatase 2A catalytic subunit beta isoform;Serine/threonine-protein phosphatase

PRKAR1A	cAMP-dependent protein kinase type I-alpha regulatory subunit;cAMP-dependent protein kinase type I-alpha regulatory subunit, N-terminally processed
PSMD11	26S proteasome non-ATPase regulatory subunit 11
PTGES3	Prostaglandin E synthase 3
RAB11B	Ras-related protein Rab-11B
RAC1	Ras-related C3 botulinum toxin substrate 1;Ras-related C3 botulinum toxin substrate 3
RGS6	Regulator of G-protein signaling 6;Regulator of G-protein signaling 7
ROCK2	Rho-associated protein kinase 2
SEPT5	Septin-5
SERPINB1	Leukocyte elastase inhibitor
SNCA	Alpha-synuclein
SPTBN1	Spectrin beta chain, non-erythrocytic 1
STEAP3	Metalloreductase STEAP3
STK4	Serine/threonine-protein kinase 4;Serine/threonine-protein kinase 4 37kDa subunit;Serine/threonine-protein kinase 4 18kDa subunit
SYTL4	Synaptotagmin-like protein 4
TAGLN2	Transgelin-2
TAGLN3	Transgelin-3
TCEB2	Transcription elongation factor B polypeptide 2
TGFB1	Transforming growth factor beta-1;Latency-associated peptide
TKFC	Bifunctional ATP-dependent dihydroxyacetone kinase/FAD-AMP lyase (cyclizing);ATP-dependent dihydroxyacetone kinase;FAD-AMP lyase (cyclizing)
TMED8	Protein TMED8
TPM1	Tropomyosin alpha-1 chain
TSPAN15	Tetraspanin-15
TTYH3	Protein tweety homolog 3;Protein tweety homolog
TUBB4B	Tubulin beta-4B chain
TXNDC5	Thioredoxin domain-containing protein 5
TXNL1	Thioredoxin-like protein 1
UGGT1	UDP-glucose:glycoprotein glucosyltransferase 1
USP5	Ubiquitin carboxyl-terminal hydrolase 5
YWHAG	14-3-3 protein gamma;14-3-3 protein gamma, N-terminally processed
ZYX	Zyxin

ENHANCED

ACTR1A	Alpha-centractin
ACTR2	Actin-related protein 2
ALOX12	Arachidonate 12-lipoxygenase, 12S-type
ANXA7	Annexin A7
APOB	Apolipoprotein B-100;Apolipoprotein B-48
ARPC4-TTLL3	Actin-related protein 2/3 complex subunit 4
ATP2A3	Sarcoplasmic/endoplasmic reticulum calcium ATPase 3
ATP5A1	ATP synthase subunit alpha, mitochondrial
ATP5B	ATP synthase subunit beta, mitochondrial;ATP synthase subunit beta
BSG	Basigin
CANX	Calnexin
CAPN1	Calpain-1 catalytic subunit
CAPZA2	F-actin-capping protein subunit alpha-2
CCL5	C-C motif chemokine 5;RANTES(3-68);RANTES(4-68)
CCT2	T-complex protein 1 subunit beta
CCT3	T-complex protein 1 subunit gamma
CCT4	T-complex protein 1 subunit delta
CCT7	T-complex protein 1 subunit eta
CCT8	T-complex protein 1 subunit theta
CD36	Platelet glycoprotein 4
CD55	Complement decay-accelerating factor
CYB5R3	NADH-cytochrome b5 reductase 3;NADH-cytochrome b5 reductase 3 membrane-bound form;NADH-cytochrome b5 reductase 3 soluble form
DBN1	Drebrin
DCTN2	Dynactin subunit 2
DPYSL2	Dihydropyrimidinase-related protein 2
EEF1A1P5	Putative elongation factor 1-alpha-like 3;Elongation factor 1-alpha 1;Elongation factor 1-alpha;Elongation factor 1-alpha 2
EEF1D	Elongation factor 1-delta
F2	Prothrombin;Activation peptide fragment 1;Activation peptide fragment 2;Thrombin light chain;Thrombin heavy chain
FLNB	Filamin-B
FYN	Tyrosine-protein kinase Fyn
GMPR	Mannose-1-phosphate guanyltransferase beta

GNAQ	Guanine nucleotide-binding protein G(q) subunit alpha
GP9	Platelet glycoprotein IX
GPX1	Glutathione peroxidase 1;Glutathione peroxidase
GTPBP2	GTP-binding protein 2
HADHB	Trifunctional enzyme subunit beta, mitochondrial;3-ketoacyl-CoA thiolase
HLA-B	HLA class I histocompatibility antigen, B alpha chain
HLA-C	HLA class I histocompatibility antigen, C alpha chain
HSP90AA1	Heat shock protein HSP 90-alpha
HSP90AB1	Heat shock protein HSP 90-beta
IDH2	Isocitrate dehydrogenase [NADP], mitochondrial
ITGA2B	Integrin alpha-IIb;Integrin alpha-IIb heavy chain;Integrin alpha-IIb light chain, form 1;Integrin alpha-IIb light chain, form 2
JAM3	Junctional adhesion molecule C
MAOB	Amine oxidase [flavin-containing] B
MDH2	Malate dehydrogenase, mitochondrial;Malate dehydrogenase
NNT	NAD(P) transhydrogenase, mitochondrial
PACSIN2	Protein kinase C and casein kinase substrate in neurons protein 2
PDCD10	Programmed cell death protein 10
PF4	Platelet factor 4;Platelet factor 4, short form
PHB2	Prohibitin-2
PNP	Purine nucleoside phosphorylase
PPIF	Peptidyl-prolyl cis-trans isomerase F, mitochondrial
PRDX3	Thioredoxin-dependent peroxide reductase, mitochondrial
PSMA7	Proteasome subunit alpha type-7
PSMD2	26S proteasome non-ATPase regulatory subunit 2
PTGIR	Prostacyclin receptor
PTPRJ	Receptor-type tyrosine-protein phosphatase eta;Protein-tyrosine-phosphatase
PTTG1IP	Pituitary tumor-transforming gene 1 protein-interacting protein
RAB14	Ras-related protein Rab-14
RAB1A	Ras-related protein Rab-1A
RAB27B	Ras-related protein Rab-27B
RAB32	Ras-related protein Rab-32
RAB5C	Ras-related protein Rab-5C
RAB6B	Ras-related protein Rab-6B
RAB7A	Ras-related protein Rab-7a
RAB8B	Ras-related protein Rab-8B
RAC2	Ras-related C3 botulinum toxin substrate 2
RALB	Ras-related protein Ral-B
RDH11	Retinol dehydrogenase 11

RHOA	Transforming protein RhoA
SAR1A	GTP-binding protein SAR1a
SCAMP2	Secretory carrier-associated membrane protein 2
SLC16A3	Monocarboxylate transporter 4
SLC2A3	Solute carrier family 2, facilitated glucose transporter member 3
SLC44A1	Choline transporter-like protein 1
SMIM1	Small integral membrane protein 1
SRI	Sorcin
STIP1	Stress-induced-phosphoprotein 1
STOM	Erythrocyte band 7 integral membrane protein
STX11	Syntaxin-11
SUSD1	Sushi domain-containing protein 1
TCEB1	Transcription elongation factor B polypeptide 1
TPP2	Tripeptidyl-peptidase 2
TREML1	Trem-like transcript 1 protein
TUBB	Tubulin beta chain
UGP2	UTP--glucose-1-phosphate uridylyltransferase
VCP	Transitional endoplasmic reticulum ATPase
VDAC1	Voltage-dependent anion-selective channel protein 1
VDAC3	Voltage-dependent anion-selective channel protein 3
